# Chloramine chemistry as a missing link in atmospheric chlorine cycling

**DOI:** 10.1126/sciadv.adv4298

**Published:** 2025-10-29

**Authors:** Yijing Chen, Men Xia, Jinghui Zhang, Epameinondas Tsiligiannis, Cheng Wu, Chao Yan, Runlong Cai, Guangjie Zheng, Yuyang Li, Junchen Guo, Zhaojin An, Yiran Li, Xinyan Zhao, Qipeng Qu, Chenjie Hua, Zongcheng Wang, Shuxiao Wang, Yongchun Liu, Lina Cao, Kebin He, Markku Kulmala, Mattias Hallquist, Tao Wang, Douglas Worsnop, Jingkun Jiang

**Affiliations:** ^1^ State Key Laboratory of Regional Environment and Sustainability, School of Environment, Tsinghua University, Beijing 100084, China.; ^2^ Department of Chemistry and Molecular Biology, University of Gothenburg, Gothenburg 40530, Sweden.; ^3^ Institute for Atmospheric and Earth System Research/Physics, Faculty of Science, University of Helsinki, Helsinki 00014, Finland.; ^4^ Aerosol and Haze Laboratory, Beijing Advanced Innovation Center for Soft Matter Science and Engineering, Beijing University of Chemical Technology, Beijing 100029, China.; ^5^ Nanjing-Helsinki Institute in Atmospheric and Earth System Sciences, Nanjing University, Nanjing 210023, China.; ^6^ Department of Intelligent Edge Cloud, Chinatelecom Cloud Technology Co. Ltd., Beijing 100010, China.; ^7^ Shanghai Key Laboratory of Atmospheric Particle Pollution and Prevention (LAP3), Department of Environmental Science and Engineering, Fudan University, Shanghai 200438, China.; ^8^ School of Engineering and Applied Sciences, Harvard University. Cambridge, MA 02138, USA.; ^9^ Department of Civil and Environmental Engineering, The Hong Kong Polytechnic University, Hong Kong 999077, China.; ^10^ Aerodyne Research Inc., Billerica, MA 01821, USA.

## Abstract

Chlorine radicals (Cl**·**) profoundly affect atmospheric oxidation capacity. Chloramines, especially trichloramine, are emerging precursors of Cl**·**. However, their sources and roles in the atmosphere remain elusive. This study presents field evidence of primary emissions and explicit secondary production pathways of atmospheric trichloramine in Beijing, supplemented by observations from New Delhi and reanalysis of measurements in Toronto. We demonstrate that the sequential chlorination reactions initiated by molecular chlorine and ammonia in atmospheric aerosols are a major source of trichloramine. The trichloramine produced in aerosols is a source of gaseous trichloramine and serves as an intermediate during the conversion from molecular chlorine to Cl**·**, while direct trichloramine emissions constitute a previously overlooked source of Cl**·**. Overall, chloramine chemistry alters the Cl**·** production mechanism and represents a crucial missing pathway to Cl**·** worldwide.

## Teaser

Chloramines from emissions and secondary production represent an overlooked component in atmospheric chlorine chemistry.

## INTRODUCTION

Reactive halogen species, comprising chlorine, bromine, and iodine species, substantially affect global environmental issues, such as the Antarctic ozone (O_3_) hole (*1*), new particle formation (*2*), and climate change (*3*). Among these, chlorine radical (Cl**·**), as a strong atmospheric oxidizer (*4*), degrades volatile organic compounds (VOCs) and methane (CH_4_) (*5*), contributing to the formation of surface O_3_ (*6*) and secondary organic aerosols (SOAs) (*7*). Lacking direct measurements of Cl**·**, current evaluations of the impact of Cl**·** on air quality and climate typically rely on the observation of its photolabile precursors, such as nitryl chloride (ClNO_2_) (*8*), molecular chlorine (Cl_2_) (*9*), and bromine chloride (BrCl) (*10*). Recent studies in UK (*11*) and Canada (*12*) reveal an emerging group of reactive chlorines in the atmosphere, i.e., chloramines, including trichloramine (NCl_3_), dichloramine (NHCl_2_), and monochloramine (NH_2_Cl), with field observations in Canada identifying NCl_3_ as a considerable source of Cl**·** (*12*). However, current observations of chloramines are limited to the aforementioned clean urban sites (*11*, *12*), leaving uncertainty about the prevalence and role of chloramines in polluted urban areas, characterized by elevated aerosol loadings and higher chemical complexity.

In addition, the source of atmospheric NCl_3_ remains poorly understood. Chloramines (mainly NH_2_Cl and NHCl_2_) are primarily adopted for industrial chemical synthesis (*13*) and drinking water disinfection (*14*). NCl_3_ is a typical volatile and irritating disinfection by-product commonly observed in the indoor air of swimming pools (*15*) and food processing facilities (*16*) and during surface bleach cleaning processes (*17*, *18*). The chloramines produced indoors are likely transported to the atmosphere via ventilation or infiltration, representing primary emissions of atmospheric chloramines. However, it remains unclear why appreciable levels of NCl_3_ are observed even when direct emissions have been largely ruled out (*12*). This indicates pronounced secondary formation of NCl_3_ in the atmosphere, while the detailed chemical mechanism has not been investigated. This knowledge gap hinders current air quality models from examining the regional or global impact of chloramines.

In this study, we report appreciable levels of NCl_3_ in Beijing and New Delhi, uncovering its sources and roles in the atmosphere. NCl_3_ prevails in both cities, and we identify NCl_3_ from both direct emissions and secondary formation. Combining field observations, machine learning, and a newly developed multiphase box model, we further elucidate the secondary formation mechanism of NCl_3_. We find that aqueous reactions involving reactive chlorines [Cl_2_ and hypochlorous acid (HOCl)] and ammonia (NH_3_) occur spontaneously in acidic aerosols, which represents a major source of atmospheric chloramines. The NCl_3_ produced from these chemical processes functions as an intermediate in Cl**·** production. In addition, directly emitted NCl_3_ comprises an emerging source of Cl**·**. As for other chloramines, NH_2_Cl represents a photostable chlorine reservoir, while NHCl_2_ can contribute to Cl**·** production via photolysis. Overall, chloramine photolysis accounts for a substantial part in Cl**·** production rate [P(Cl**·**)], with higher contributions (>30%) occurring during periods with relatively low fine particulate matter (PM_2.5_) concentrations in both Beijing and New Delhi. Last, we predict chloramine mixing ratios in various atmospheric environments, highlighting the broad impact of chloramine chemistry. We conclude that the proposed chloramine chemistry is ubiquitous because of the widespread coexistence of the precursors and fills a missing link in atmospheric chlorine cycling.

## RESULTS

### Primary emission and secondary production of atmospheric NCl_3_

We observed persistent appearance of NCl_3_ in both Beijing and New Delhi. Measured mixing ratios of NCl_3_ reached up to 35 parts per trillion by volume (pptv) in Beijing and 124 pptv in New Delhi, with average values of 1.2 and 5.9 pptv, respectively (figs. S1 and S2). These levels are comparable to those reported during the summer in Toronto (average of 4.8 pptv) (*12*). Higher levels of NCl_3_ were observed in summer compared to winter in both Beijing (1.3 times higher) and Toronto (*12*) (4.0 times higher). The average diurnal patterns of NCl_3_ at all three sites closely track the solar radiation, with elevated NCl_3_ levels observed at night, consistent with the rapid photolysis of NCl_3_. The average photochemical lifetime of NCl_3_ is 6.6, 7.2, and 5.1 min, respectively, at noontime during the summer Beijing, winter Beijing, and New Delhi observations. Correspondingly, NHCl_2_ has longer photochemical lifetime, i.e., 46.5, 51.3, and 36.4 min, respectively. An obviously longer lifetime for NH_2_Cl (several hours) is expected because of its photostable nature. To gain further insights into the sources of atmospheric NCl_3_, we conducted detailed case studies based on the comprehensive field measurements in Beijing.

**Fig. 1.**
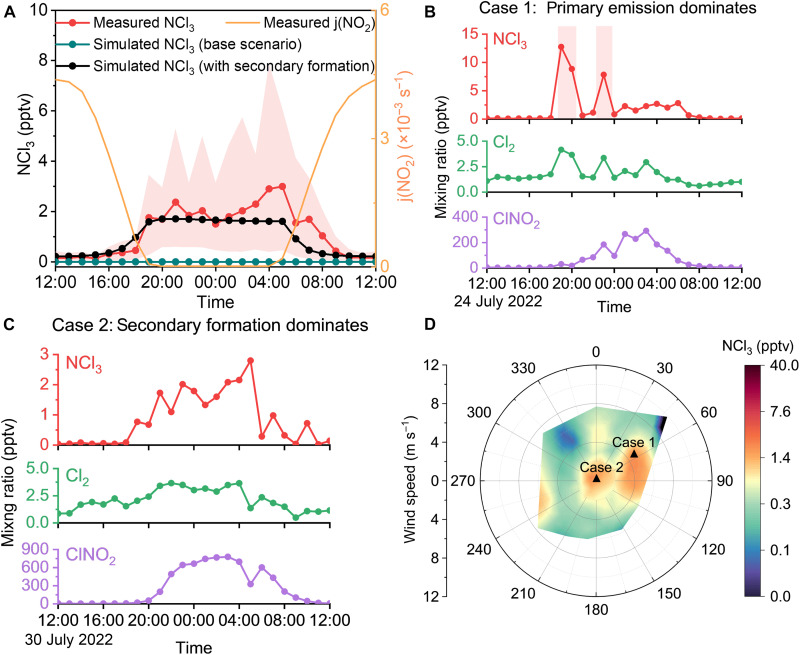
Field characterizations of primary and secondary productions of NCl_3_ in summer Beijing. (**A**) Comparison of measured and simulated daily averages of NCl_3_ in the base scenario and with the secondary formation mechanism considered. The shaded area represents the 10th and 90th percentiles of the measured NCl_3_. The orange line denotes the measured average diurnal variation in NO_2_ photolysis frequency [*j*(NO_2_)]. A case indicates (**B**) primary emission dominates and (**C**) secondary production dominates the occurrence of NCl_3_. (**D**) Wind rose plot color-coded by NCl_3_ mixing ratios. Cases shown in (B) and (C) were marked as triangles in (D) accordingly. A NCl_3_ spike (case 1) is identified when its deviation from the local median of hourly mixing ratios within an 8-hour moving window (centered on the spike, ±4 hours) exceeds three times the median of the absolute differences from the median of the observations, considering only positive deviations. The 8-hour moving window is applied to account for the background variability. The others are classified into case 2.

We identified two distinct cases of NCl_3_, named case 1 and case 2, characterized by different dominating patterns. In case 1, we found sporadic high spikes of NCl_3_ (Fig. 1B and figs. S3C and S4), which were attributable to primary emissions. The NCl_3_ in case 1 mainly comes from the northeast of our sampling site (Fig. 1D and fig. S3E), where an indoor swimming pool and a sports center are located (fig. S5), indicating the transport of NCl_3_-rich plumes emitted from these facilities. Within these plumes, only Cl_2_ shows sharp and synchronous increases together with NCl_3_, consistent with the behavior of Cl_2_ arising from direct emissions in previous field measurements (*19*). The synchronous spikes of NCl_3_ and Cl_2_ mirror those seen during our bleach spraying experiments designed to simulate direct emissions, where a mixture of NaOCl solution and trace amounts of NH_3_**·**H_2_O was atomized indoors while sampling the indoor air (fig. S6). Similar intermittent NCl_3_ spikes have also been observed in Toronto, originating from emissions of local sources (*12*). In addition, box modeling results show that secondary productions merely account for 15% on average of the observed spikes (see Materials and Methods, modeling scenario 2), which confirms that direct emissions are the main source of NCl_3_ in case 1. As chlorine agents are widely used as disinfectants (*20*) (table S1), chloramines originating from indoor-to-outdoor transport deserve more attention.

We identified another distinct pattern of NCl_3_, categorized as case 2. The occurrence frequency of case 2 (~91%) is substantially higher than that of case 1 (~9%) throughout the campaign. We observed persistent nocturnal increases in NCl_3_ in case 2 (Fig. 1C and figs. S3D and S7), mostly under calm wind conditions (Fig. 1D and fig. S3E). This pattern exhibits smooth variations in NCl_3_, contrary to the sporadic spikes shown in case 1. The average NCl_3_-to-NHCl_2_ and NCl_3_-to-Cl_2_ ratios (2.1 and 1.6, respectively) in case 2 are considerably higher than those in case 1 (1.0 and 0.6, respectively) (fig. S8), which further distinguishes these two cases.

Atmospheric secondary production is possibly the dominating source of NCl_3_ in case 2. The synchronous variations in NCl_3_, Cl_2_, and ClNO_2_ in case 2 indicate shared sources for these species. ClNO_2_ typically serves as an indicator for the secondary formation of reactive chlorines, as its predominating source is the heterogeneous uptake of dinitrogen pentoxide (N_2_O_5_) on chloride-containing aerosols (*21*). Previous studies have shown coproduction of Cl_2_ and ClNO_2_ (*22*), suggesting that NCl_3_ formation occurs through similar secondary processes. Box modeling results demonstrate that ~64% of the observed average NCl_3_ levels in case 2 were reproduced when including the secondary formation mechanism (see Materials and Methods, modeling scenario 2).

Residential, municipal, and commercial facilities in urban areas are regularly using chlorinating agents to disinfect surfaces (table S1), which could lead to large-scale emissions of chloramines that would likely exhibit similar smooth variations in the observation dataset, thereby complicating our identification of secondary chloramine production (case 2) happened outdoors. Given the limited simultaneous indoor and outdoor chloramine measurements (*17*) and the lack of chloramine emission inventories, it is challenging to conclude the importance of indoor diffuse sources to outdoor chloramine levels.

### Chemical mechanism of NCl_3_ production in aerosols

**Fig. 2.**
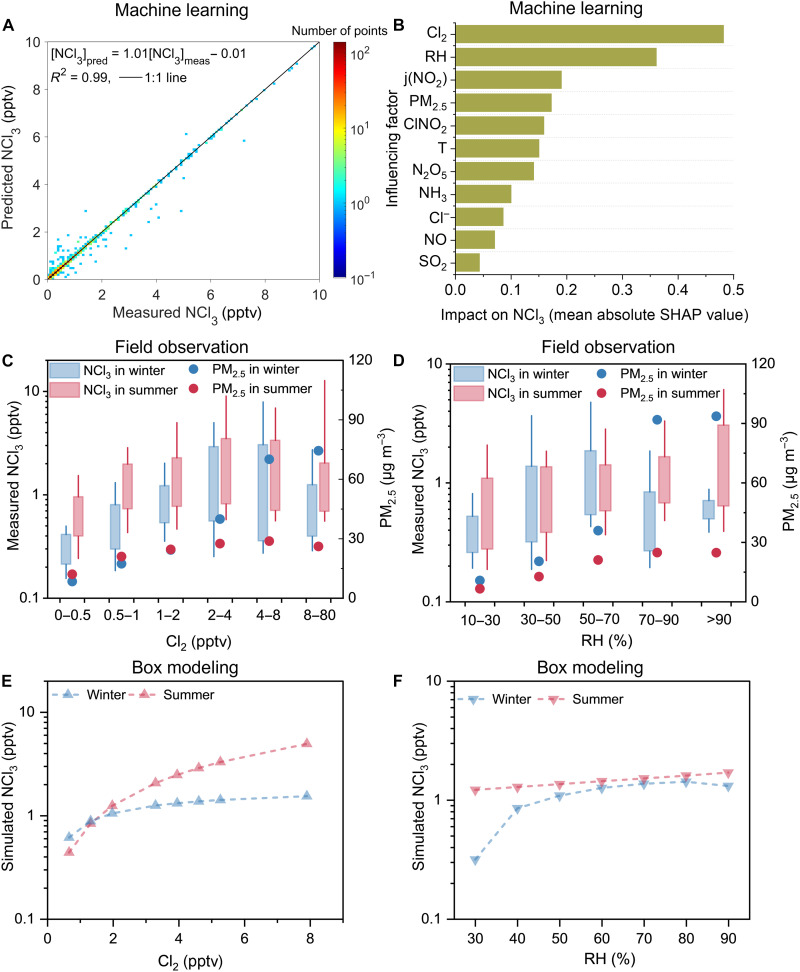
The critical role of Cl_2_ and RH in driving NCl_3_ formation. (**A**) Correlation between the predicted and measured NCl_3_ in Beijing based on machine learning. (**B**) Impacts of top 10 influencing factors on the NCl_3_ level, ranked by their overall importance (mean absolute SHAP value) in descending order. Dependency of measured nocturnal NCl_3_ on (**C**) Cl_2_ and (**D**) RH. Average PM_2.5_ concentrations in each interval are shown as dots in (C) and (D). Sensitivity tests of nocturnal (**E**) Cl_2_ and (**F**) RH on the output NCl_3_ in the box model. Winter nighttime is defined as 00:00 to 7:00 and 17:00 to 24:00, while summer nighttime is 00:00 to 5:00 and 19:00 to 24:00.

Cl_2_ and relative humidity (RH) were identified as the key driving factors for NCl_3_ formation through Shapley Additive exPlanations (SHAP) values from machine learning analysis. The machine learning model, which well predicted NCl_3_ concentrations (Fig. 2A), shows that Cl_2_ and RH are the most critical factors among 20 observational parameters (Fig. 2B). However, we could hardly guarantee that the discarded features (e.g., pH and aerosol liquid water content) were trivial on the basis of this data-driven approach. Cl_2_ is indicated as the dominating precursor of NCl_3_, while the dependence of NCl_3_ on RH emphasizes the aqueous chemistry nature of NCl_3_ production. Consistently, our observations in Beijing show an overall positive relationship between nocturnal NCl_3_ and Cl_2_ as well as RH (Fig. 2, C and D) during periods with lower PM_2.5_ concentrations. NCl_3_ mixing ratios are unexpectedly lower during the haze period despite the elevated Cl_2_ and RH. Specifically, this inverse relationship was observed during an episode that shifted between hazy and clean days in winter (fig. S9). The decreased NCl_3_ level in polluted periods was accompanied by lower aerosol acidity [pH 5 to 6, estimated using ISORROPIA II (*23*)], while higher levels of NCl_3_ were observed in cleaner periods with higher aerosol acidity (pH 4 to 5). This result is consistent with previous laboratory experiments in aqueous solutions that NH_2_Cl and NHCl_2_ with a *p*K**_*a*_ of 19.7 and 11.8, respectively (where *K*_*a*_ is the acid dissociation constant) (*24*), would get further protonated to NCl_3_ with the decrease of solution pH (*25*, *26*). Analogously, in the atmosphere, the decreased aerosol acidity probably favors the production and volatilization of NH_2_Cl and NHCl_2_ while reducing NCl_3_ formation. Consistently, we observed a positive correlation between nocturnal gaseous NHCl_2_ and NCl_3_ both in Beijing and New Delhi (fig. S10, A and B). The NCl_3_-to-NHCl_2_ ratio in Beijing decreases with increasing aerosol pH (fig. S10C), suggesting that NHCl_2_ and NCl_3_ share similar production pathways and their relative amounts are dependent on aerosol pH.

Combining our results from observations, machine learning, and box modeling, alongside the previous findings in the aqueous solutions, we propose that secondary production of NCl_3_ is initiated via the aqueous-phase reactions between Cl_2_ and NH_3_ in atmospheric acidic aerosols. Our machine learning results agree with previous laboratory generation of NCl_3_ in aqueous solutions where Cl_2_ reacted with excess NH_3_ (*25*, *27*) and the theoretical predictions that water assisted in favoring chloramine formation (*28*). The proposed mechanism was also qualitatively validated by our and the previous (*11*) laboratory experiments, where chloramines were observed following the absorption of Cl_2_ into an acidified (NH_4_)_2_SO_4_ slurry (text S1 and fig. S11). In addition, we applied a multiphase box model to quantitatively simulate NCl_3_ formation from the reaction between total ammonia and Cl_2_ in atmospheric aerosols. The model reproduces the observed average diurnal variations in NCl_3_ when incorporating the proposed secondary formation mechanism (Fig. 1A). In contrast, no secondary NCl_3_ is produced in the base scenario (Fig. 1A and fig. S3A).

It should be acknowledged that while the model-simulated NCl_3_ levels exhibit reasonable consistency with the observations, the simulation bears certain uncertainties due to our limited knowledge about atmospheric chloramine chemistry. The chloramine module in our model relies largely on a simplified aqueous-phase system where the reaction kinetics are derived from aqueous solutions. Chlorination of other potential organic nitrogen agents and related matrix components may be missing in the model, which may lead to an exaggeration of the self-reactions between chloramines and the radical intermediates. In addition, the possibility of gas-phase chloramine productions or indoor to outdoor emissions cannot be ruled out as the atmospheric relevant kinetic studies and emission inventories are currently lacking, which merits further investigation.

**Fig. 3.**
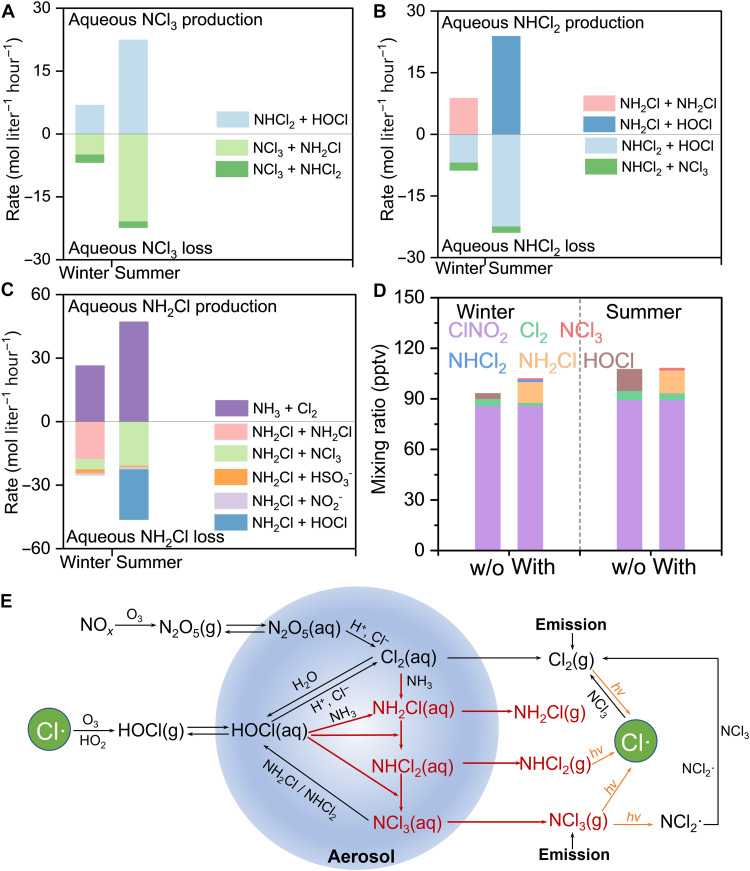
Sources, transformations, and impacts of atmospheric chloramines. Average aqueous production and loss rates of (**A**) NCl_3_, (**B**) NHCl_2_, and (**C**) NH_2_Cl from different reaction pathways in the box model. Reactions that contribute less than 5% to the total budgets, such as phase transfer, are not shown. (**D**) Comparison of the average total reactive chlorine levels with or without (w/o) incorporating the proposed secondary mechanism. (**E**) Schematic of atmospheric evolutions of chloramines. The proposed mechanism is illustrated in red.

The proposed secondary mechanism, i.e., aqueous-phase reactions between NH_3_ and Cl_2_ in atmospheric aerosols (Fig. 3), provides an explanation for the observed driving factors of NCl_3_ production. Sensitivity tests using the box model, where the inputs of Cl_2_ and RH are varied, show an increase in the simulated NCl_3_ in the scenarios under higher Cl_2_ or RH settings (Fig. 2, E and F), which agrees with the field-observed positive dependency of NCl_3_ on both Cl_2_ and RH. The influence of the other precursor (NH_3_) on NCl_3_ is found minimal, especially when NH_3_ mixing ratios exceed ~10 parts per billion (ppb) in the model (fig. S12A). With NH_3_ in great excess, Cl_2_, rather than NH_3_, becomes the limiting precursor for NCl_3_ formation. Meanwhile, the NH_3_ levels fall within a suitable range to ensure enough aerosol acidity, so that sufficient protonation of NH_2_Cl and NHCl_2_ retains them until they are reacted completely to produce NCl_3_.

The proposed mechanism also accounts for seasonal variations in the NCl_3_ level. The model-simulated NCl_3_ production rates in summer were ~2 times greater than those in winter (Fig. 3, A to C). This simulation result suggests that the enhanced secondary production of NCl_3_ from aqueous reactions of NHCl_2_ and HOCl outcompetes the effects of increased dilution and photolysis in summer, aligning with the relatively higher levels of NCl_3_ observed in summer compared to winter in both Beijing and Toronto. Model sensitivity tests show that the elevated secondary production rates of NCl_3_(aq, aqueous phase) in summer are partly driven by higher ambient temperature (fig. S12B). In addition, stronger aerosol acidity in summer (average pH of 3.0 in summer and 4.3 in winter Beijing), as discussed previously, also enhances secondary NCl_3_ production by keeping NH_2_Cl and NHCl_2_ protonated sufficiently, thereby promoting their nucleophilic substitution by HOCl to yield NCl_3_. Consistently, higher NCl_3_ levels found in summer Toronto were accompanied by higher summertime aerosol acidity (*29*). The same seasonal behavior of NCl_3_ may also exist in many urban environments beyond Beijing and Toronto. These findings in the seasonal behavior of NCl_3_, alongside its driving factors discussed previously, provide insights into the chemical mechanisms underlying atmospheric NCl_3_ production.

The NCl_3_ of secondary origin acts as a previously unrecognized intermediate in atmospheric chlorine cycling, fundamentally altering Cl**·** production pathways. The secondary production of NCl_3_ occurs via the stepwise Cl_2_(aq)-initiated chlorination of NH_3_(aq), sequentially producing NH_2_Cl(aq), NHCl_2_(aq), and NCl_3_(aq) (Fig. 3E). Cl_2_ and NCl_3_ transform into each other throughout a diurnal cycle. The NCl_3_ produced in the aqueous phase volatilizes into the gas phase, accumulating at night. During the following morning, the accumulated gaseous NCl_3_ contributes to Cl**·** formation either through direct photolysis or indirectly via producing Cl_2_. Furthermore, we evaluated the impact of the proposed mechanism on P(Cl**·**) using the box model (see Materials and Methods, modeling scenario 3). Results show that the total average mixing ratios of the simulated reactive chlorines increase by 9% in winter and 2% in summer, respectively, after incorporating the chloramine formation mechanism, mainly in the form of nonphotolabile NH_2_Cl (Fig. 3D and fig. S13). The simulated net P(Cl**·**) remained nearly unchanged because the increase in P(Cl**·**) from production of photolabile NHCl_2_ and NCl_3_ is offset by the additional consumption of Cl_2_ and HOCl, along with the production of nonphotolytic NH_2_Cl. However, NH_2_Cl could be eventually converted to NHCl_2_ and NCl_3_ through further chlorination, releasing the stored Cl**·** and affecting downstream areas.

## DISCUSSION

### Ubiquitous chloramines reshape atmospheric chlorine chemistry

The chloramine chemistry bridges a missing link in atmospheric chlorine cycling, altering the production pathways of Cl**·** and contributing substantially to P(Cl**·**). As is shown in fig. S14, 11% of P(Cl**·**) is attributable to chloramine chemistry during New Delhi observations, while this proportion increases to 16 and 19%, respectively, during winter and summer observations in Beijing. NCl_3_-related processes dominated the contribution of chloramines to P(Cl**·**) in Beijing, while NHCl_2_ photolysis became more important in New Delhi, contributing 9% to P(Cl**·**), and even more important in Toronto, with a contribution of 22 to 26%. The interconversion among Cl_2_, HOCl, and chloramines throughout a day adds an important route to the overall chlorine cycling. A portion of Cl_2_ and HOCl converts into chloramines before releasing Cl**·**, while NCl_3_ photolysis regenerates Cl_2_, contributing to 21 and 39% of Cl_2_ production on average in Beijing and Toronto, respectively. The chloramine-involved cycling creates more Cl**·** reservoirs and fundamentally reshapes Cl**·** formation pathways. The derivatization of Cl_2_ to chloramines prevents its consumption in competing reactions that do not yield Cl**·**. For instance, Cl_2_ can oxidize sulfur(IV) species, enhancing aerosol sulfate production and reducing Cl_2_ to chloride in this process (*30*). Chloramine formation competes with such Cl_2_-consuming reactions, thereby maintaining Cl**·** production. Although the simulated net P(Cl**·**) shows minimal change at the observation site after incorporating secondary chloramine production, the notable increase in the generated nonphotolytic NH_2_Cl likely serves as a relatively long-lived Cl**·** reservoir, exerting broader impacts of chlorine chemistry across expanded areas. In addition, primary chloramine emissions are new sources of Cl**·**.

**Fig. 4.**
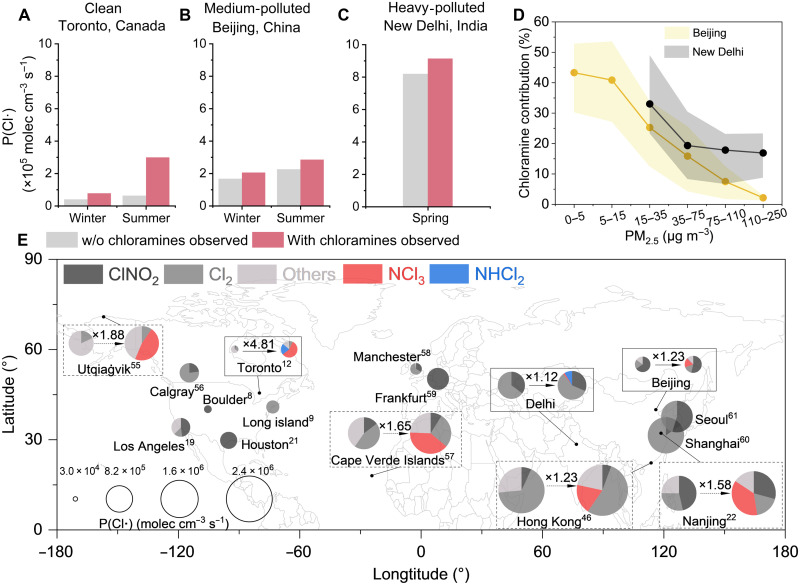
Substantial contributions of chloramines to Cl· production in multiple locations. Average contribution of chloramines to P(Cl·) in (**A**) Toronto, (**B**) Beijing, and (**C**) New Delhi. (**D**) PM_2.5_ dependency of chloramine contribution to P(Cl·) in Beijing and New Delhi. The solid lines indicate the average values, and the shaded areas indicate the 25th and 75th percentiles. Molec, molecules. (**E**) Worldwide enhancement of P(Cl·) with and without chloramines being observed. “Others” in (E) means the combination of all other Cl· production pathways except for ClNO_2_-, Cl_2_-, NCl_3_-, and NHCl_2_-related processes (e.g., ClO· + NO). In solid rectangles where chloramine measurement data are available, the average summertime P(Cl·) is shown for Beijing and Toronto, and the average wintertime P(Cl·) is shown for New Delhi. Simulated chloramine mixing ratios are used in the dashed rectangles. References (*8*, *9*, *12*, *19*, *21*, *22*, *46*, *55*–*61*) are presented as superscripts in (E).

The importance of chloramine chemistry in atmospheric chlorine cycling is dynamic, evolving with changing air quality. Elevated aerosol loadings in polluted locations and/or during hazy periods generally coincide with a higher abundance of ClNO_2_ (fig. S13B) and reduced aerosol acidity (*31*), both of which limit the impact of chloramine chemistry. The contribution of chloramines to P(Cl**·**) was higher when PM_2.5_ concentrations were at a relatively lower level in both Beijing and New Delhi, accounting for 42% of P(Cl**·**) during cleaner periods (PM_2.5_ ≤ 15 μg m^−3^) and 11% during polluted periods (PM_2.5_ ≥ 75 μg m^−3^) (Fig. 4D). Consistently, chloramine photolysis contributed to 64, 18, and 11% of the observed P(Cl**·**) in Toronto (campaign average PM_2.5_ = 10 μg m^−3^), Beijing (26 μg m^−3^), and New Delhi (70 μg m^−3^), respectively, demonstrating higher contributions in cleaner areas. The higher abundance of chloramines in cleaner areas or periods may pose health risks, e.g., deterioration of lung function and asthma (*32*), and affect the chemical evolution of aerosol-phase reduced organic nitrogen compounds through chloramination reactions, altering SOA composition (*33*).

The secondary chloramine production mechanism highlights its broad impacts, given the ubiquitous presence of the ingredients (NH_3_, Cl_2_, and atmospheric aerosols). To illustrate this, we applied the developed box model to predict chloramine mixing ratios in more places, i.e., Hong Kong, Nanjing, Cape Verde, and an Arctic site, based on availability of observational data. Results show that secondary chloramine production contributes to P(Cl**·**) by 19, 37, 39, and 47%, respectively, at these locations. These results likely represent a lower limit for the importance of chloramines, since primary emissions have not been considered because of a lack of emission profiles in these regions. Previous field studies on reactive chlorines overlooked the existence and the contributions of chloramines to P(Cl**·**) (Fig. 4). Consequently, field campaigns that do not account for chloramines would likely cause a biased understanding of Cl**·** production pathways (see Eq. 1 in Materials and Methods). The extrapolation of chloramine presence to other locations indicates their potential impacts; however, precise quantification of their contribution to P(Cl**·**) remains uncertain because of the limited observational data, spatial coverage, and inherent model uncertainties.

The findings of this study, particularly regarding the sources and impacts of chloramines, underscore the need for further investigation into atmospheric chloramine chemistry. First, chloramine observations remain scarce, with the field data mainly limited to this work and two previous studies (*11*, *12*). Considering the observed discrepancy in chloramine levels between clean and polluted periods or regions, comprehensive field measurements of chloramines, including vertically resolved campaigns, are essential to characterize Cl**·** formation routes in different environments. Second, the identified primary emissions and secondary production of chloramines highlight the need to develop chloramine emission inventories and further investigate the kinetics of chloramine chemistry involving Cl_2_ and ammonium-containing aerosols. Third, direct experimental measurements of the photolysis rate coefficients of chloramines (especially NHCl_2_) in the gas phase are warranted to better constrain P(Cl**·**), therefore enabling reevaluation of the impact of chlorine cycling on atmospheric oxidation. In this study, C_2_H_3_ClO_2_, presumably chloroacetic acid, was frequently observed in both Beijing and New Delhi (fig. S15), providing direct evidence of Cl**·** participating in VOC oxidation (*34*). Last, the role of chlorine chemistry in air quality and human health under a changing climate also merits further investigation.

## MATERIALS AND METHODS

### Field measurements of chloramines and related species

#### Beijing

Field observations were conducted in a typical urban residential area of Beijing from 22 January to 28 February and 1 June to 17 August 2022. The sampling site was situated on the fourth (top) floor of a building at Tsinghua University (THU) (fig. S5, THU site). No higher buildings or intense pollution sources were nearby, and the nearest major traffic road was ~1 km away. An indoor swimming pool was located ~0.6 km northeast of the sampling site. Details of the site were described in previous publications (*35*, *36*).

Reactive chlorines (including NCl_3_, NHCl_2_, Cl_2_, and ClNO_2_) and N_2_O_5_ were measured as iodide clusters by a Filter Inlet for Gases and AEROsols-Iodide-Long Time of Flight Mass Spectrometer (FIGAERO-I^−^-LToF-CIMS, Aerodyne Research and TOFWERK AG; referred to as CIMS) (*37*). This study focuses on the gas-phase data, since the reactive chlorine species mostly dispersed in the gas phase due to their low Henry’s law constants (table S2). The reactive chlorines were identified unambiguously via peak fitting (mass accuracy < 2.5 parts per million) and were further verified by the detected isotopic ratios compared to the natural isotopic abundance of Cl (fig. S16). Iodide (I^−^) and iodide water cluster (I·H_2_O^−^) were the dominant reagent ions. The sum of I^−^ and I·H_2_O^−^ was normalized to the approximate magnitude of 200,000 counts per second (cps) in both field measurements and calibrations to eliminate the influence of variations in reagent ions. Background signals were determined by overflowing the inlet with zero air under different RH and were found ignorable compared to respective ambient signals. Detection limits (1-hour interval) ranged from 0.1 to 7.4 pptv, depending on the season and the specific species (table S3). Ambient NHCl_2_ was reported only in winter since its signals were largely (>90%) below the detection limit in summer and were thus excluded from further analysis. Calibrations of the reported species (NHCl_2_ and NCl_3_) were described as follows, while calibrations of Cl_2_, N_2_O_5_, and ClNO_2_ were provided in the Supplementary Materials. Additional details of the field measurements in Beijing are shown in text S1.

The principle of chloramine calibrations was referred to previous studies (*38*, *39*), with the experiment setup shown in fig. S17. Fresh NHCl_2_ solutions were prepared by mixing 300 ml of 6 mM NaOCl solution and 300 ml of 1.5 mM (NH_4_)_2_SO_4_ solution, both of which were pH buffered to 4.80 before mixing. Fresh NCl_3_ solutions were first prepared by mixing 300 ml of 6 mM NaOCl solution (buffered at pH 3.82) and 300 ml of 1 mM (NH_4_)_2_SO_4_ solution (also buffered at pH 3.82), followed by dilution with ultrapure water to achieve lower gaseous NCl_3_ concentrations suitable for CIMS measurements. Fresh chloramine solutions were placed in a two-port gas-tight bottle, and the volatile NHCl_2_ or NCl_3_ were bubbled out by a N_2_ flow of ~171 standard cubic centimeters per minute (SCCM). A portion (~128 SCCM) of the main outflow was bypassed to two sequential gas absorption bottles that both contained 20 ml of 5 mM Na_2_SO_3_ (pH adjusted to 6.73 using H_2_SO_4_) to quantitatively reduce chloramines to chloride (*39*). We therefore determined the generated gaseous chloramine level based on the volume of the Na_2_SO_3_ solutions, the chloride concentrations within the Na_2_SO_3_ solutions, and the absorption time. Ion chromatography (Dionex Aquion RFIC, Thermo Fisher Scientific) with a limit of detection (LOD) of 0.007 mg liter^−1^ was used to determine the chloride concentration. The chloride level in the second Na_2_SO_3_ solution was always below the LOD, which indicated the complete trap of chloramines in the first Na_2_SO_3_ solution. The chloride concentration (2.3 to 12.1 mg liter^−1^) in the first Na_2_SO_3_ solution was thus used to determine the sensitivity of chloramines. The remaining chloramine-containing outflow (~43 SCCM) was diluted with zero air and delivered to the CIMS inlet. The observed NHCl_2_ signal remained stable throughout the bubbling process. However, because of the high volatility of NCl_3_ (with a $H_{v}^{\text{px}}$ value 551.7 atm, almost 290 times higher than that of NHCl_2_) (*40*), NCl_3_ degassed rapidly during the bubbling, which sharply reduced the NCl_3_ concentrations in the solution. The CIMS signal of gaseous NCl_3_ thus decreased accordingly (fig. S17). Integration of the NCl_3_ signal over the calibration period was therefore applied to match the total amounts of NCl_3_ introduced into CIMS. It was noted that Cl_2_ also accounted for ~12.5 and ~ 18.5% of the total measured chloride concentrations in the first Na_2_SO_3_ solution during NHCl_2_ and NCl_3_ calibrations, respectively, which were subtracted when calculating chloramines’ sensitivities. The sensitivity of NHCl_2_ and NCl_3_ was 0.12 and 2.22 cps pptv^−1^ at a H_2_O mixing ratio of 0.55%, respectively.

Dependence of the sensitivity of the above species on ambient water vapor mixing ratios and potential artifact tests was performed and described in detail in text S1. Apart from N_2_O_5_ and NHCl_2_, the sensitivities of Cl_2_, ClNO_2_, and NCl_3_ were not substantially dependent on ambient H_2_O. The relationship between the H_2_O mixing ratio and the analyte’s sensitivity was derived from least-squares fitting functions and was applied to the ambient data. The sensitivity of NHCl_2_ dropped sharply with the increase in the H_2_O mixing ratio (fig. S18), which partly explained why NHCl_2_ levels were below the instrument’s detection limit (7.4 pptv) in summer. Consistently, the model-simulated NHCl_2_ levels in summer range from 0.22 to 0.41 pptv, probably due to complete conversion to NCl_3_. We therefore think that omitting NHCl_2_ in summer in Beijing will not cause a substantial bias of P(Cl**·**). No noticeable increase in the chloramine signals was observed when sampling indoor air or injecting Cl_2_ or HOCl through a used inlet tube (figs. S19 and S20), thus excluding potential artifacts of chloramines.

Auxiliary measurements were obtained from the west campus of the Beijing University of Chemical Technology (the BUCT site) (*41*) and the nearest national monitoring station (the Wanliu Station) besides the THU site. The BUCT site and the Wanliu Station are also situated in urban areas, which are ~7 and ~5 km southwest of the THU site, respectively. Data collected at the BUCT site and the Wanliu Station were used to supplement any missing measurements from the THU site. All measurement data were cross-compared in different sites (if available) for quality control and were averaged to 1-hour intervals before analysis. Detailed supporting measurements in Beijing are summarized in text S1 and table S4.

#### New Delhi

We conducted field observations of reactive chlorines (NCl_3_, NHCl_2_, Cl_2_, and ClNO_2_) and N_2_O_5_ in New Delhi to supplement the analysis in Beijing and provide broader implications. These reactive chlorines were measured by a FIGAERO-I^−^-HR-ToF-CIMS (also referred to as CIMS) on the campus of Indian Institute of Technology Delhi (IITD site) from 23 February to 14 March 2023 (text S2). The ion-molecule reactor of the CIMS was humidified by a constant flow of ~10 SCCM of water vapor–saturated N_2_ to minimize the potential variation of sensitivities and background signals due to fluctuations in ambient RH. The background signals (hourly average ± SD) for NCl_3_, NHCl_2_, Cl_2_, ClNO_2_, and N_2_O_5_ were 0.05 ± 0.15, 0.31 ± 0.29, 0.31 ± 0.30, 1.43 ± 1.72, and 6.10 ± 2.15 cps, respectively. A steady flow of acetic acid (0.3 liters min^−1^) from a permeation source was injected into the CIMS on 6 days (27 and 28 February and 5, 10, 12, and 14 March 2023), with a sensitivity (0.27 ± 0.05 cps pptv^−1^) variance of 17.7%. The sensitivities of N_2_O_5_ and ClNO_2_ were directly calibrated, while the sensitivities for Cl_2_, NCl_3_, and NHCl_2_ were estimated using their relative sensitivity to ClNO_2_. Existing direct calibration results [this study and Wang *et al.* (*12*)] suggest consistent sensitivity ratios among the inorganic chlorinated species. An uncertainty of 11, 32, and 41% was estimated for the sensitivities of Cl_2_, NCl_3_, and NHCl_2_ based on the differences in their relative sensitivity ratios to ClNO_2_ in this study (Beijing campaign) and the Toronto campaign (*12*).

#### Measurement uncertainties

The uncertainties are mainly caused by calibrations (10 to 41%) and variation in instrument sensitivities (~17%), while uncertainties from peak fitting (<2%) of the mass spectrum, inlet artifacts (<5%), and flow rate measurements (<2%) are negligible (text S1). The measurement uncertainties for NCl_3_, NHCl_2_, Cl_2_, ClNO_2_, and N_2_O_5_ mixing ratios were 47, 47, 37, 20, and 30%, respectively, in Beijing and 50, 60, 40, 20, and 30%, respectively, in New Delhi, aligning with previous studies using CIMS that reported uncertainties of 30 to 50% (*42*, *43*).

### Multiphase chemical box model

A chemical box model was developed to investigate the secondary production and loss of chloramines in Beijing and evaluate their impacts on P(Cl**·**) in seven locations (Fig. 4E). Principles of the model are briefly introduced as follows, while detailed mechanisms, inputs, outputs, and sensitivity tests are documented in the Supplementary Materials (text S3). Overall, the chemical mechanisms concerning chloramines are based on current knowledge, which may not be complete considering limited research on atmospheric chloramines.

#### Model construction

The model was constructed under the framework for zero-dimensional atmospheric modeling (F0AM; version 4.0.2) (*44*) by incorporating newly developed modules to address chloramine chemistry. The master chemical mechanism v3.3.1 was adopted as the initial chemical mechanism (*45*), including explicit gas-phase reactions of trace gases, radicals, and VOCs. We incorporated our previously compiled halogen module to describe the gas-phase chlorine chemistry (*46*), including Cl**·** production via the photolysis of reactive chlorines (e.g., ClNO_2_ and Cl_2_ without chloramines) and Cl**·** loss by reactions with O_3_ and VOCs. Following the default treatment in F0AM, a simplified scheme was adopted to consider the heterogeneous reactions on aerosol surfaces (e.g., ClONO_2_ uptake). A fixed uptake coefficient was assigned to each relevant gaseous species (*47*), while the yield of gas-phase products was assumed as unity and the aqueous product was not considered. An additional gas-phase mechanism (table S5) was appended to simulate the gas-phase photochemistry of chloramines, i.e., the photolysis of NCl_3_ and NHCl_2_ and subsequent reactions involving Cl**·** and Cl_2_. The photolysis frequency of NCl_3_ and NHCl_2_ was calculated by integrating the corresponding absorption cross sections, quantum yields, and actinic photon fluxes (dependent on both the location and time). We labeled the Cl_2_ produced by chloramine photochemistry to evaluate its role as a Cl_2_ source.

The box model was constrained by the hourly observation data and calculated parameters in winter and summer campaigns in Beijing, respectively. The input data included meteorological factors [T, RH, boundary layer height, and *j*(NO_2_)], gas-phase compositions, and aerosol aqueous-phase components [e.g., NH_4_^+^(aq)] and properties (e.g., pH and liquid water content) (table S5). Gas-phase compositions were obtained by direct observations, including trace gases, VOCs, and reactive chlorines, while the aqueous-phase parameters were calculated by a thermodynamic model (ISORROPIA II) (*23*) before being used in our box model. To stabilize the reactive intermediates (mostly radicals) that were not constrained in the model, we performed three consecutive runs and adopted the result from the last run for further analysis.

#### Compilation of multiphase reactions

A multiphase module was introduced to quantify the production and loss of chloramines in Beijing, which consisted of aqueous-phase reactions and phase transfer processes. Four categories of aqueous reactions were compiled from the literature and were summarized in the Supplementary Materials:

1) Reversible acid dissociation reactions (Ad1 to Ad68) of 34 species, e.g., HOCl;

2) Irreversible aqueous reactions (Aq1 to Aq280) without involving chloramines;

3) Chloramine-related aqueous reactions (Ca1 to Ca38);

4) Aqueous photolysis reactions (Ph1 to Ph10).

We assume that chloramine-related reactions in aerosol aqueous phase follow the same rate constants as those occurring in water. All available chloramine reaction kinetics in aqueous solutions were compiled, with rate constants fixed according to the literature. In aqueous solutions, chloramine formation proceeds via the stepwise chlorination of NH_3_ or organic nitrogen (*48*). In our model, except for total ammonia (NH_3_ and NH_4_^+^), we did not consider the chlorination of other nitrogenous compounds, e.g., amino acids and urea, due to their unknown concentrations in ambient aerosols. We do not expect urea to be important in our study, as urea mainly comes from agricultural activities, while our observation sites are all located in urban areas. Nevertheless, we could not exclude amino acids or other nitration agents. The lack of other nitration agents in our model may bias the results by overweighting the self-reactions among chloramines, which merits future studies. The effect of aerosol organics on aqueous oxidant levels was considered, while their effects on the multiphase system (e.g., viscosity) or phase transfer of inorganic species could hardly be considered using current knowledge. For all reaction modules, temperature-dependent rate constants were adopted (if available) to account for the distinct temperature ranges in winter and summer. We performed sensitivity tests to evaluate the potential uncertainty in the rate constants of chloramine-related reactions, which showed no appreciable influence on major conclusions (text S3).

The phase transfer of chloramines and related species (except for ClONO_2_) was simulated using a kinetic approach following Soni *et al.* (*49*). Both gas-to-aerosol and aerosol-to-gas phase transfers were considered simultaneously. The rate of change of gaseous and aqueous species was separately simulated in two steps during one phase transfer because of the different units of species in the gas (in parts per billion) and aqueous phase (in moles per liter).

#### Modeling scenarios

We conducted the model simulations under four scenarios for respective purposes, as summarized in table S6. First (scenario 1), to evaluate the impact of chloramines on P(Cl**·**), all observed gas-phase reactive chlorines (i.e., NCl_3_, NHCl_2_, Cl_2_, and ClNO_2_) in Beijing, New Delhi, and Toronto (*12*) were constrained in the model. As P(Cl**·**) is determined by the mixing ratio of reactive chlorines and respective photolysis frequencies (Eq. 1), the supplementary data, such as NO*_x_* and VOCs, do not affect the relative importance of chloramines in contributing to P(Cl**·**). Notably, the term [NCl_3_] × *j*(NCl_3_) is a simplified representation of Cl**·** production from the first step of NCl_3_ photolysis, as subsequent photochemical reactions following NCl_3_ photolysis also contribute to Cl**·** production

$$\begin{array}{c} \begin{array}{c} P\left(\text{Cl}\cdot \right)=2\;\times \;\left[{\text{Cl}}_{2}\right]\;\times \;j\left({\text{Cl}}_{2}\right)\;+\;\left[{\text{ClNO}}_{2}\right]\;\times j\;\left({\text{ClNO}}_{2}\right)\;+\\ \left[{\text{NCl}}_{3}\right]\;\times \;j\left({\text{NCl}}_{3}\right)\;+\;\left[{\text{NHCl}}_{2}\right]\;\times \;j\left({\text{NHCl}}_{2}\right) \end{array} \end{array}$$ (1)

Second (scenario 2), we aimed to investigate the formation and loss mechanisms of chloramines, taking Beijing as an example due to the comprehensive dataset there. In this case, the modeled mixing ratios exclusively represent the contribution from secondary sources, since no direct emissions of chloramines were added to the model. In this scenario, we constrained other reactive chlorine species (i.e., Cl_2_ and ClNO_2_) and auxiliary measurements (e.g., meteorological factors, VOCs, etc.), while NHCl_2_ and NCl_3_ were not constrained but simulated in the model. The contribution of primary emissions to NCl_3_ spikes (case 2) was estimated as follows (Eqs. 2 to 4)

$${\text{[}{\text{NCl}}_{3}\text{]}}_{\text{secondary}}={\text{[}{\text{NCl}}_{3}\text{]}}_{\text{model simulated}}$$ (2)

$${\text{[}{\text{NCl}}_{3}\text{]}}_{\text{primary}}={\text{[}{\text{NCl}}_{3}\text{]}}_{\text{observed}}-{\text{[}{\text{NCl}}_{3}\text{]}}_{\text{secondary}}$$ (3)

$${\text{Contribution}}_{\text{primary emission}}=\frac{{\text{[}{\text{NCl}}_{3}\text{]}}_{\text{primary}}}{{\text{[}{\text{NCl}}_{3}\text{]}}_{\text{observed}}}$$ (4)

In addition, we performed sensitivity tests to examine the influencing factors of chloramines in Beijing and evaluate the model performance. We adjusted the potential influencing factors of NHCl_2_ and NCl_3_ (i.e., Cl_2_, RH, NH_3_, and temperature) in the model to observe how chloramines respond. Further technical details of the sensitivity tests are explained in text S3.

Third (scenario 3), we aimed to evaluate the impact of the proposed secondary mechanism in Cl**·** production. In this scenario, we merely constrained the supporting measurements but ceased to constrain any reactive chlorines, including chloramines, ClNO_2_, and Cl_2_. We then ran the model with (case 1) and without (case 2) considering the aqueous chemistry of chloramines (reaction Ca1 to Ca38; table S6). As the proposed mechanism depleted Cl_2_ and HOCl while producing chloramines, we evaluated its net effect on Cl**·** production by comparing P(Cl**·**) from all simulated reactive chlorines in case 1 and case 2.

Last (scenario 4), we simulated chloramines’ contributions to P(Cl**·**) in Hong Kong, Nanjing, Cape Verde, and at an Arctic site where relevant supporting data (e.g., observed Cl_2_, ClNO_2_, and NH_3_ levels) were available. The model inputs for these simulations are summarized in table S8. In this scenario, we simulated the average diurnal profile of chloramines and calculated their contribution to P(Cl**·**) along with all other reactive chlorines. For this part of simulations, we assume that our model is applicable beyond Beijing where we can reproduce the observed chloramine mixing ratios. Uncertainties may arise from the accuracy of the observation data in these places and the potential unknown competing chemistry and the lack of direct emissions of reactive chlorine species in the model.

### Machine learning

An Extreme Gradient Boosting Regression (XGBoost) (*50*) model coupled with a SHAP algorithm (*51*) was used to investigate the key factors influencing NCl_3_ (framework shown in fig. S21). XGBoost is a popular machine learning model widely used for predicting air pollutant levels (*52*, *53*), while SHAP is a powerful approach to interpreting model predictions and has recently been introduced to help explore the influencing factors of target species in atmospheric chemistry (*54*). Dataset preparation, model construction, and SHAP interpretation were briefly introduced as follows, while technical details were provided in text S4. Overall, the XGBoost model performed well in predicting NCl_3_ (*R*^2^ = 0.99 on the whole dataset, i.e., the training, validation, and test datasets; Fig. 2A; *R*^2^ = 0.829 on the test dataset; table S9) and was robust against various splits of the training and validation dataset [10-fold cross-validation (CV), *R*^2^ = 0.796]. In contrast, the *R*^2^ of the multivariate linear regression method on the test dataset was 0.34, which further confirmed that the machine learning model can better capture the nonlinear impacts of different factors on NCl_3_.

Twenty features, including hourly meteorological parameters, trace gases, aerosol physicochemical properties, *j*(NO_2_), and the season index (table S10), were used as input variables to predict NCl_3_. Short-period missing values (≤3 hours) were filled by linear interpolation, while long period (>3 hours) of data missing and outliers were discarded, which ultimately resulted in a total dataset of 1327 lines. Since the features showed large distributional differences in winter and summer (fig. S22), the observed data in both seasons were randomly sampled in equal proportions (stratified random sampling) to construct the training, validation, and test datasets. The test dataset accounted for 11 January and the remaining dataset were used for 10-fold CV, model selection, and optimization. In the CV process, the dataset was divided into 10 equally sized subsets, nine of which were used for training and one for validation. This process was repeated until each subset had been validated once. The XGBoost model was lastly chosen for its better performance over other models (tables S11 and S12). Given the intricate interdependencies among the 20 features, a Greedy algorithm was implemented to eliminate redundant or unimportant features, thereby refining the model input set. During the feature selection process, each of the 20 features was independently incorporated into the XGBoost model to assess their individual impact. The feature that mostly improved the model performance was selected (fig. S23). The selected features were then expanded iteratively until the model performance could not be further improved with the added feature. Eleven of the initial 20 features were ultimately selected, and the model performance was improved (table S9). Last, the XGBoost model was trained with the optimized hyperparameters and selected features on the combined training and validation dataset and evaluated on the test dataset (*R*^2^ = 0.829).

The SHAP method was adopted to interpret the XGBoost model predictions. Here, the SHAP value quantitatively described the impacts of the influencing factors on the predicted NCl_3_, expressed as follows

$$y_{i}=y_{\text{base}}+\sum g\left(x_{i,j}\right)$$ (5)

where *y_i_* was the predicted NCl_3_, *y*_base_ was the average of all predicted NCl_3_, and *g*(*x*_*i*,*j*_) was the SHAP value that suggested the contribution of influencing factor *j* in a certain sample *i* to the predicted NCl_3_. A positive or negative SHAP value denoted a positive or negative impact on NCl_3_, respectively, and a zero SHAP value represented the feature has no impact on the NCl_3_ level.

## Supplementary Materials

### This PDF file includes:

Supplementary Text Figs. S1 to S25 Tables S1 to S14 Legend for table S15 References
