# Supplementary material for: Chloramine chemistry as a missing link in atmospheric chlorine cycling
Source: Sci Adv. 2025 Oct 29;11(44):eadv4298. doi: 10.1126/sciadv.adv4298 (PMC12571055; doi:10.1126/sciadv.adv4298)
Supplement: Supplementary file 1 — Supplementary Text Figs. S1 to S25 Tables S1 to S14 Legend for table S15 References [file sciadv.adv4298_sm.pdf]

Science Advances
AAAS

Supplementary Materials for

Chloramine chemistry as a missing link in atmospheric chlorine cycling

Yijing Chen *et al.*

Corresponding author: Jingkun Jiang, jiangjk@tsinghua.edu.cn

Sci. Adv. **11**, eadv4298 (2025)
DOI: 10.1126/sciadv.adv4298

This PDF file includes:

Supplementary Text
Figs. S1 to S25
Tables S1 to S14
Legend for table S15
References

Supplementary Text

Text. S1 Details of measurements in Beijing

1. Instrument configurations

The Figaero-I^--LToF-CIMS (hereafter referred to as CIMS) was housed near a side wall at the THU site (40.00°N, 116.33°E; ~10 m above the ground level) to minimize inlet length. The PFA sampling tube (1.5 m-long, 0.156-inch inner diameter) penetrated the wall to draw ambient air. A total of 10 slpm of ambient air passed through the inlet (residence time: ~0.1 s) with ~2 lpm being directed into the CIMS for analysis, while the excessive air was pumped out. This inlet design aimed to reduce the contact time between ambient air and the sampling inlet, thus minimizing inlet artifacts. Additionally, we replaced the sampling tube weekly to further prevent such artifacts.

Reagent ions were generated by passing 2.3 lpm of CH_3I in dry N_2 through a soft X-ray ion source (L9491, Hamamatsu). The ion-molecule reaction chamber (IMR) was maintained at 300 mbar and 50 °C. The RH and T of the excess sampling flow were recorded by an in-line sensor and used to calibrate the measured species, accounting for their sensitivity variations with ambient RH. The gas-phase data was recorded at a rate of 1 Hz for 20 min every hour and was averaged over 1-hour intervals for further analysis. The average mass resolution was ~9500. HOCl was not reported in this study due to the interference from a dominant signal of the adjacent fluorine-containing ion ($C_3F_5O_3^-$) on the mass spectrum. Nevertheless, other species reported here were free from such interference.

2. Instrument stability

Backgrounds and tracer compound signals were regularly measured during the campaign to monitor the instrument stability. Background signals were recorded by switching the instrument inlet to dry zero air for 5 min, once every 1~2 days in summer and every four days in winter. The background signals remained stable throughout the campaign, with hourly average (± standard deviation) of 0.34 ± 0.13, 0.08 ± 0.05, 0.42 ± 0.14, 0.42 ± 0.35, and 3.14 ± 1.35 cps for NCl_3, $NHCl_2$, Cl_2, $ClNO_2$, and N_2O_5, respectively. Background measurements with zero air under varying RH showed ignorable variance, confirming their reliability for ambient measurements.

Additionally, we used O_3 as a tracer compound to track the instrument stability by comparing the on-site and post-campaign sensitivity variance in O_3. Our laboratory calibrations and the previous studies (*62*) showed that the sum of IO_x^- ($IO^- + IO_2^- + IO_3^-$) signals measured by I^--CIMS correlated linearly with the injected O_3 concentrations, and the sensitivity of O_3 was water-dependent (Fig. S24). Ambient O_3 was continuously monitored by the CIMS and a collocated O_3 analyzer (49i, Thermo Fisher) throughout the campaign, with calibrations performed during the same period (June of 2023) as chloramine calibrations. A strong positive correlation (R^2 = 0.90) and overall consistency (slope ~1) were found between the CIMS-derived and directly measured (by 49i) hourly O_3 concentrations (Fig. S24), confirming the instrument stability of CIMS during the campaign and the comparability of on-site and post-campaign

calibrations. The measurement uncertainty due to sensitivity drifts was estimated to be approximately 17.1% (relative standard deviation of the ratio between CIMS-derived and directly measured O_3 concentrations). The intercept on the x axis in Fig. S24C reflected the detection limit of O_3 (3σ, 1 hour) at ~6.6 ppb.

3. Reagents and materials

All chemicals used in this study were purchased from Sigma-Aldrich in reagent grade. Ultrapure water (resistivity 18.2 MΩ·cm at 25 °C, GenPure UV/UF, Thermo Scientific) was used as the solvent for calibrations and testing. The acetate buffer solutions (total concentration 0.2 M, pH 4.8 for $NHCl_2$ and pH 3.7 for NCl_3) used for the chloramine calibration were prepared by mixing sodium acetate and acetic acid solutions in varying ratios following the Henderson–Hasselbalch equation. The buffer pH was further verified by a pH meter (HI2002-01, HANNA instruments) and was found to be within ±5% of the reference value. Chloride concentrations during chloramine calibrations were determined using Ion Chromatography (Thermo Scientific Dionex Aquion RFIC System). To prevent column overloading from high concentrations of sulfite ions, samples were diluted tenfold with ultrapure water prior to injection. An external standard calibration curve of chloride (0.5, 2, 5, 10, 20, and 50 mg L^{-1}, r = 0.999) was constructed before each sample analysis, and the chloride level in samples fell well within the linearity range of the calibration curve.

4. Calibrations of related species and uncertainty analysis

Cl_2. A five-point external calibration of Cl_2 was performed using a permeation tube (VICI Metronics, certified permeation rate of 120 ng min^{-1} at 40 ^oC) (Fig. S18C). The permeation tube was put in a glass U-tube submerged in a water bath maintained at 40 ^oC, with a steady flow of ~200 sccm N_2 passing through it. This Cl_2-containing flow was then diluted to a Cl_2 concentration range of 240~700 ppt by a mixture of dry and humidified zero air with varying flow rates before entering CIMS. The sensitivity of Cl_2 was 2.64 cps ppt^{-1} at an H_2O mixing ratio of 0.55%. The calibration uncertainty from the permeation source was estimated to be about 20%.

N_2O_5 and $ClNO_2$. N_2O_5 was synthesized *in situ* in a dark flow tube via the reaction of O_3 with excess NO_2, and the generated N_2O_5 was quantified from the decrease in NO_2 (measured by Model 42i, Thermo Scientific) following the addition of O_3 (*63*). The NO_2-to-NO molybdenum converter in the 42i analyzer was replaced with a photolytic NO_2 converter (Air Quality Design Inc.) to avoid interferences in NO_2 measurements (*64*). A quantifiable amount of $ClNO_2$ was produced by passing a known amount of N_2O_5 over a humidified NaCl slurry bed placed in a Teflon tube (*65*). The production yield of $ClNO_2$ was assumed to be unity. The calibration tubing was flushed with dry zero air for at least 24 hours in advance to minimize the N_2O_5 hydrolysis to produce HNO_3 on the tubing surfaces. We maintained the NO_2 concentrations while varied the O_3 concentrations to obtain different levels of N_2O_5 for calibrations, which were then diluted with zero air at varying RH before entering CIMS. The tubing connecting the CIMS inlet and N_2O_5 in the diluted flow was kept as short as possible

(~0.3 m) to minimize N_2O_5 hydrolysis on the tubing. The sensitivity of N_2O_5 and $ClNO_2$ was 0.66 cps ppt^{-1} and 1.27 cps ppt^{-1}, respectively, at an H_2O mixing ratio of 0.55%. As N_2O_5 (<10%) was inevitably converted to HNO_3 during the calibration, an uncertainty of 10% was estimated for N_2O_5 and $ClNO_2$ measurements.

$NHCl_2$ and NCl_3. The detailed calibration setup has been described in the Method part of the main text. Uncertainties of this method could arise from:

(1) Chloride originated from species other than chloramines. We minimized potential interferences by keeping the target chloramine as the dominant species, achieved by adjusting the pH and Cl-to-N ratio in the chloramine solution. Additionally, we tracked and accounted for the signals of other chlorinated species (i.e., Cl_2, HOCl, HCl, and $ClNO_2$) when calculating chloride from chloramines. Except for Cl_2, other chlorinated species remained at the background level during the calibration. It was estimated that a fraction (<20%) of the trapped chloride originated from Cl_2, meaning that uncertainties in Cl_2 sensitivity contributed to the overall uncertainty in chloramine sensitivity.

(2) Trapping efficiency of chloramines using Na_2SO_3 solution and quantification of chloride using IC. We performed additional tests by directly injecting Cl_2 from the permeation tube into the Na_2SO_3 solution and detecting the chloride concentration using IC. The Cl_2 permeation rate derived from the Na_2SO_3 trapping method (98 ng min^{-1}) was consistent with the KI titration–UV spectrophotometry method (*10*) (93 ng min^{-1}) and within a 23% difference of the certified reference value of 120 ng min^{-1}.

An overall estimate of the calibration uncertainty was about 30%. $NHCl_2$ and NCl_3 were calibrated in the concentration range about 1~2 orders of magnitude higher than the ambient level, while $ClNO_2$ and N_2O_5 were calibrated within the ambient concentration range. Multi-point calibrations of $ClNO_2$, Cl_2, N_2O_5, $NHCl_2$, and NCl_3 all showed strong linearity ($R^2 \geq 0.99$) with the calibration curves passing through the origin. Therefore, we assumed the calibrated sensitivities are applicable to ambient measurements.

5. Sensitivity dependence on ambient water vapor content

The sensitivity of certain analytes measured using CIMS can vary substantially, depending on the water vapor pressure in IMR, which is controlled by the humidity of the sampled ambient air (*66*). The varying sensitivity is caused by different reactivity of the analytes towards I^- and $I \cdot H_2O^-$ in IMR. The RH and T of the excessive sample gas were continuously recorded by an in-line RH/T sensor during field observations. The recorded RH and T were converted to water mixing ratios according to the ideal gas law and the water saturation vapor pressure over different temperatures (*67*). The water mixing ratios ranged from 0.10% to 0.50% (RH 13% to 93%) in winter and 0.15% to 3.01% (RH 5% to 100%) in summer, aligning with the range adopted in calibrations (water mixing ratios from 0.09% to 2.30%, RH 2% to 85%). Then, we applied the sensitivity dependency on water mixing ratio obtained in the laboratory calibrations to ambient conditions.

6. Potential artifact tests

Indoor activities such as swimming and bleach cleaning (*11, 15, 18*), followed by the transport of contaminants to the outdoor environment, could interfere with field measurements of chloramines. However, no such activities occurred inside the sampling building during the field campaign, and chloramine levels were constantly below the detection limits during indoor air sampling (Fig. S19).

To assess potential inlet artifacts, we passed laboratory-generated $NHCl_2$ or Cl_2 through a used sampling tube at the same flow rate as in the field campaign before entering the CIMS inlet. The inlet flow RH was about 40%. Results showed that negligible $NHCl_2$ (~3%) was lost on the used sampling tube, and no NCl_3 was observed (Fig. S20). Furthermore, no detectable chloramines were observed when Cl_2 passed through the used tube, ruling out the potential conversion of Cl_2 to chloramines via reactions with adsorbed NH_3 on the inlet surfaces. The wall loss of NCl_3 was not determined experimentally, as we could not generate a stable NCl_3 air stream. However, we anticipated negligible wall loss of NCl_3, based on its higher volatility compared to $NHCl_2$. Additionally, an experiment was performed to investigate the potential inlet formation of NCl_3 and $NHCl_2$ from HOCl uptake. HOCl was synthesized by bubbling ~171 sccm N_2 through a phosphate-buffered (pH = 6.80) NaOCl solution (4.00~4.99% free chlorine, Sigma-Aldrich), and the HOCl-containing gas was diluted and introduced to the CIMS (*68*). Neither NCl_3 nor $NHCl_2$ increased when HOCl passed through the used tube, ruling out HOCl as a source of potential chloramine artifacts.

We also measured a ~5% wall loss of $ClNO_2$ in the used sampling tube. However, fewer $ClNO_2$ inlet artifacts were expected during field observations, as the flow rate passed through the sampling tube in the field (~10 lpm) was higher than that used in laboratory tests (~5 lpm).

7. Laboratory experiments simulating atmospheric chloramine formation

Bleach spraying. A series of indoor bleach-spraying experiments were conducted to characterize the direct emission pattern of chloramines. Household bleach solutions (free chlorine content: 80-120 mg L^{-1}) and $NH_3 \cdot H_2O$ (0.1% by mass) were used to generate reactive chlorines. Four spraying events, each lasting for approximately 2~6 minutes, were conducted. For each event, ~100 ml bleach solution was placed in a beaker, and a nozzle (shown in the red rectangle box in Fig. S6) was used to atomize the solution around the instrument inlet. For the third spraying event, we added ~100 ml $NH_3 \cdot H_2O$ solution to the beaker and subsequently poured it off. Substantial increases in NCl_3 and $NHCl_2$ were instantly observed upon adding ~100 ml bleach solution to the same beaker, even without physical atomizing. This likely resulted from the reactions between trace amounts of NH_3 (residues on the inner wall of the beaker) and the excessive free chlorine. The interval between sequential spraying events lasted for 30-40 min to ensure the reactive chlorine species decay to background levels.

Cl_2 + acidified $(NH_4)_2SO_4$ slurry. To qualitatively test the hypothesis of atmospheric multiphase chloramine production, an experiment was implemented

involving the reaction between Cl_2 and acidified $(NH_4)_2SO_4$ slurry. The slurry was prepared by adding ~1.2 ml pH 2.13 H_2SO_4 solution to ~5.0 g $(NH_4)_2SO_4$ particles (Fig. S11). A continuous flow of ~100 sccm Cl_2, with a mixing ratio of ~408 ppb Cl_2 in dry N_2, was passed through the $(NH_4)_2SO_4$ slurry. The resulting gas stream was further diluted with ~12 lpm of zero air to ~3.4 ppb of Cl_2 before entering the CIMS. Appreciable $NHCl_2$ (~7.2 ppb) and a small amount of NCl_3 (~0.015 ppb) were produced during the experiment. This outcome supports the hypothesis of multiphase production of chloramines, with $NHCl_2$ formation favored over NCl_3 in this case. The predominance of $NHCl_2$ is likely attributed to the acidity of the $(NH_4)_2SO_4$ slurry which was assumed to have a pH of approximately 4~5, a range favorable for $NHCl_2$ production.

8. Supporting measurements in Beijing

This section outlines the auxiliary measurements conducted in Beijing, including VOCs, water-soluble ions, and meteorological parameters. VOCs were measured by either online Vocus-PTR (TOFWERK AG) or offline canister sampling followed by detection using a gas chromatography system equipped with a mass spectrometric detector (GC-MS, Agilent Tech., 7890/5975, USA) and a flame-ionization detector (FID). The Vocus-PTR measurements were performed on a 2-hour cycle at the rate of 1 Hz, with 110 min dedicated to ambient air sampling, 5 min for zero gas, and 5 min for calibration gas. Further details of this method were described in our previous publication (*69*). For VOCs contained in the calibration gases (C_7H_8/C_8H_{10}/C_9H_{12}/$C_{10}H_{16}$ in winter and C_6H_6/C_7H_8/C_8H_{10}/C_9H_{12}/$C_{10}H_{16}$ in summer), the measurement uncertainties were within 3%.

For NH_2Cl, the sensitivity was determined by the ionization and ion-transmission efficiency of Vocus-PTR. The ionization efficiency was obtained by proton-transfer-reaction kinetics, and relative transmission curves were parameterized by the molecular mass of NH_2Cl (*70, 71*). The polarizability and permanent dipole moment of NH_2Cl were used as the input parameters for calculating the proton transfer rate constant (k_{PTR}) of H_3O^+ with NH_2Cl (*71*). In addition to the counting errors in mass spectrometry, the uncertainty arose from the lack of an experimental k_{PTR} value of NH_2Cl and the uncertainty in trajectory-parameterization method, resulting in an estimated overall uncertainty of ~30%.

For the offline VOC measurements, the 3.2 L summa canisters were used to collect air samples with a stable flow rate of 4.26 mL min^{-1}. A pressure gauge was used to check for air leakage in the canister before each sampling. C2-C3 compounds (C_2H_6, C_3H_8, C_2H_2) were detected by GC-FID, while other VOCs (i-C_4H_{10}, n-C_4H_{10}, i-C_5H_{12}, n-C_5H_{12}, n-C_6H_{12}) were detected by GC-MS. The calibration curves for the target VOCs were constructed using the standard substance (SPECTRA GASES Inc., USA) referenced for Photochemical Assessment Monitoring Stations (PAMS) and the US EPA TO-15 standard. The correlation coefficients of the calibration curves for all species exceeded 0.98, and the relative standard deviation (RSD) for triplicate

measurements ranged 0.5%–6.0%. Further details can be found in our previous study (*72*).

The hourly average concentrations of water-soluble ions (NO_3^-, SO_4^{2-}, NH_4^+, Cl^-, Na^+, K^+, Mg^{2+}, and Ca^{2+}) in ambient $PM_{2.5}$, along with inorganic gases (NH_3, HNO_3, HONO, and HCl), were measured using a Monitor for Aerosols and Gases in Ambient air (MARGA; Metrohm Applikon B.V., NL). The water-soluble gases were diffused into the absorption solution (0.0024% H_2O_2) through a wet rotating denuder (WRD), while the particles were collected in a steam-jet aerosol collector (SJAC). The absorption solutions from the WRD and SJAC were drawn using syringes and injected into IC with an internal standard (lithium bromide, LiBr) for analysis. The accuracy of MARGA measurements for NH_3 and HONO gases was ≤24% and ≤20%, respectively. For the eight water-soluble ions, the measurement accuracy was ≤5~7%.

Meteorological factors, i.e., T, RH, wind speed, and wind direction, were measured by a weather station (Vaisala). The boundary layer height (BLH) was retrieved by measurements of a ceilometer (Vaisala, model: CL31). The photolysis frequency of NO_2, $j(NO_2)$, was measured by a Filtered Radiometer (Metcon GmbH). The photolysis frequencies of reactive chlorine species were calculated using the TUV model, based on their respective absorption cross sections (Fig. S13) and scaled to ambient conditions using $j(NO_2)$. Trace gases were measured by on-line gas analyzers (Thermo Scientific). Aerosol surface area density was calculated using particle number size distribution measured by a Differential Mobility Particle Sizer (DMPS, TSI).

Text. S2 Details of the field measurements in India

The field measurements were conducted on the eighth floor of the main building at the Indian Institute of Technology in New Delhi (IITD; 28.54°N, 77.19°E). The IITD site represents a typical urban environment in India, affected by various anthropogenic sources such as vehicular, residential, and industrial emissions, as well as regional transport (*73*). The FIGAERO-I^--HRToF-CIMS (hereafter referred to as CIMS) was accommodated in a temperature-controlled room and was adopted to measure reactive chlorine species ($NHCl_2$, NCl_3, Cl_2, and $ClNO_2$) and N_2O_5, which were unambiguously detected as iodide adducts. Reagent ions (I^- and $I\cdot H_2O^-$) were generated by passing ~2 lpm ultrahigh purity (UHP) N_2 over a CH_3I permeation tube and further ionized by a Po-210 ion source before entering the IMR. A 10 sccm water vapor-saturated UHP N_2 was directly added to the IMR to minimize sensitivity variations due to fluctuations in the ambient water vapor content. The IMR pressure was maintained at 217 ± 20 mbar during the gas sampling period.

The CIMS used two sampling lines: one for gas measurements and the other for collecting $PM_{2.5}$. The gas sampling line consists of a PFA tube (6 mm inner diameter, 4-5 m in length) with a total inflow of 3 lpm, of which 1 lpm was injected into the IMR and the remaining 2 lpm was pumped away. The $PM_{2.5}$ collection line used a copper tube (6 mm inner diameter, 4-5 m in length) with a total inflow of 3 lpm, with 1 lpm allocated for particle collection and 2 lpm pumped away. These sampling lines were

positioned ~30 m above the ground level. For the gas measurements, an additional 1 lpm UHP N_2 dilution flow was introduced before the FIGAERO orifice to avoid titration of reagent ions by high levels of ambient pollutants (especially HNO_3) in New Delhi. Consequently, the detected signals were multiplied by a factor of 2 when calculating the ambient mixing ratios. The perfluoropentanoic acid (PFPA, $C_5HF_9O_2$) standard (detected as $I{\cdot}C_5HF_9O_2^-$, m/z 391) in UHP N_2 flow (~0.3 lpm) was injected into the main gas sampling flow every 1~2 days for mass calibration. A flow of acetic acid in UHP N_2 (~0.3 lpm) from a temperature-controlled permeation source (30 ^{0}C) was injected into the CIMS on 2/27, 2/28, 3/5, 3/10, 3/12, and 3/14 to monitor the instrument sensitivity.

The CIMS operated in a ~1.5-hour cycle, alternating between gas sampling (20 min, collecting $PM_{2.5}$ simultaneously) and particle desorption (50 min). During each gas sampling period, UHP N_2 was measured for the initial and final 2 min as backgrounds, with ambient air measurements conducted during the remaining time. The ion signals were normalized to the sum of the dominant reagent ions (I^- and $I{\cdot}H_2O^-$) at 10^6 cps, which were further background-subtracted and averaged to 1-hour intervals.

Meteorological parameters (T, RH, wind speed, and wind direction) were measured with a time resolution of 1 min at the same site during the campaign. The hourly $PM_{2.5}$ concentration and solar radiation data were retrieved from the Delhi Pollution Control Committee. The data from the nearest station, Sri Aurobindo Marg, about 3 km to the north of the measurement site, was used. Then, we converted the solar radiation to $j(NO_2)$ using the method described in Trebs et al. (*74*). The derived $j(NO_2)$ was then used to scale the photolysis frequencies of reactive chlorine species.

Text. S3 Details of the multiphase chemical box model

1. Additional mechanisms in the model

Besides the default MCM mechanism and the previously compiled reactive halogen mechanism (*46*), we incorporated additional modules, i.e., gas-phase and multiphase chloramine chemistry. Details of these modules were introduced as follows.

1.1 Gas-phase chloramine chemistry

1.1.1 Photolysis of chloramines

This module included the photolysis of NCl_3 and $NHCl_2$ and subsequent reactions of the photolysis products (Table S2). Photolysis of NH_2Cl was not considered due to its negligible photon absorption at tropospherically relevant wavelengths (*39*). The photolysis frequencies of NCl_3 and $NHCl_2$ (j_{NCl3} and j_{NHCl2}) were calculated by multiplying their absorption cross sections (σ, unit: cm^2 molec^{-1}), actinic photon fluxes (F, unit: photon cm^{-2} s^{-1} nm^{-1}), and quantum yields at specific wavelengths (ϕ). Generally, the photolysis frequency of a molecule (J, unit: s^{-1}) is expressed as follows:

$$J = \int_{\lambda_1}^{\lambda_2} F(\lambda)\sigma(\lambda)\phi(\lambda)\mathrm{d}\lambda \qquad \text{(Eq. 1)}$$

where λ_1 and λ_2 define the wavelength range under which the molecule can be photolyzed. The actinic photon flux is obtained from the Tropospheric Ultraviolet and Visible (TUV) Radiation Model.

The absorption cross section of gaseous NCl_3 is obtained from the work of Clark and Clyne (*75*), while for gaseous $NHCl_2$, no values have been reported to date. The absorption cross section of NCl_3 measured in the aqueous solutions (*75*) is about a factor of 1.4 ± 0.7 times that measured in the gas phase (*39*). We thus estimated the absorption cross section of gaseous $NHCl_2$ from those measured in the aqueous solution (*39*), applying a correction factor of 1.4. The quantum yield of NCl_3 is assumed as unity to keep consistent with the previous field study (*12*). The quantum yield of $NHCl_2$ is adopted as 0.52 at the wavelength of 301 nm (*76*), rather than assuming as unity, to provide a conservative evaluation of the photolysis rate of $NHCl_2$. Then, we divided the photolysis frequencies of NCl_3 and $NHCl_2$ by that of NO_2 and obtained a ratio of 0.579 and 0.0818, respectively. Assuming a constant j_{NCl3} / j_{NO2} and j_{NHCl2} / j_{NO2} ratio, the real-time j_{NCl3} and j_{NHCl2} were calculated using the field-observed j_{NO2} in the model. As the absorption cross-section and quantum yield of gaseous $NHCl_2$ were derived from previous measurements in the aqueous phase, we provide further justification for the scaling of the aqueous-phase $NHCl_2$ photolysis frequency (j($NHCl_2$ (aq)) to the gaseous counterpart j($NHCl_2$ (g)).

1.1.1.1 Justification in estimating gaseous σ($NHCl_2$)

The solvent has the potential to change the absorption cross section of the solute upon dissolution, which is also known as solvatochromic shift (Table R2). The extent of solvatochromism depends on the interactions between solvents and solutes (*77*).

The n→σ* electronic transition is responsible for the light absorption of $NHCl_2$ and NCl_3, and the additional energy leads to the cleavage of the N-Cl bond (*76*). As polar solvents like water are likely to stabilize the non-bonding orbit, thus lowering the ground-state energy (*78*), the absorption of $NHCl_2$ would potentially shift to shorter wavelengths in aqueous solutions compared to that in the gas phase (i.e., blue shift). Therefore, the overlap between the absorption spectra of gaseous $NHCl_2$ with the tropospheric solar radiation will likely be larger than that in the aqueous phase, implying a higher cross-section of $NHCl_2$ in the gas phase.

Additionally, a ~6 nm blue shift was observed in the absorption spectra of both $NHCl_2$ and NCl_3 in water relative to those in carbon tetrachloride (22, 25, 26), indicating comparable solvent effects for these two species. Given that the aqueous molar absorptivity of NCl_3 is 1.4 ± 0.7 times that in the gas phase (27, 25), we applied the same scaling factor to estimate the molar absorptivity of gaseous $NHCl_2$. This approach, involving the scaling of the molar absorptivity and without incorporating the blue shift, likely provides a lower-limit estimation for j($NHCl_2$ (g)).

1.1.1.2 Justification in estimating gaseous ϕ_{inn}($NHCl_2$)

The ϕ_{inn} is defined as the number of N-Cl bonds broken per photon absorbed in the parent $NHCl_2$ molecule (*76*). The value 0.54 used in the manuscript was retrieved from the ϕ_{inn} of $NHCl_2$ measured in the aqueous solutions at 300 nm (*76*). To our knowledge, no other values of ϕ_{inn} of $NHCl_2$ were available for wavelengths larger than 300 nm that were more relevant to the photolysis of gaseous $NHCl_2$ in the atmosphere. In addition, the observed ϕ_{inn} of $NHCl_2$ exhibited a linearly decreasing trend with the increase of the irradiated wavelength below 300 nm, with values of 0.93, 0.84, and 0.65 at the wavelengths of 255 nm, 265 nm, and 285 nm, respectively (*76*), which stems from the fact that the molar photon energy of the radiation decreases with increasing wavelength. Previous studies have shown that the ϕ_{inn} of species in the aqueous solutions is usually lower than the values in the gas phase due to the solvent "cage" effect, which means that the excited solutes or generated free radicals are trapped by the surrounding solvents, thus facilitating energy dissipation and recombination by colliding with the solvent molecules, resulting in a reduction in ϕ_{inn} (*79*). In this context, assuming that solvent "cage" effect is negligible and extrapolating the wavelength-dependent decreasing trend of ϕ_{inn} of $NHCl_2$ above 300 nm would represent a lower bound for the estimation of *j*($NHCl_2$ (g)), while applying a constant ϕ_{inn} of unity would be an upper bound. Our estimation in the main text using a constant ϕ_{inn} of 0.54 for all wavelengths provides a moderate evaluation of *j*($NHCl_2$ (g)).

1.1.3 Reactions between intermediates

Besides the first-step photolysis of chloramines (NCl_3 and $NHCl_2$), the model also considers further reactions involving photochemical intermediates (i.e., Cl and NCl_2) (Table S5), which release additional products, i.e., Cl and Cl_2. To calculate the proportion of ambient Cl_2 produced by reactions No. 2 - 4 in Table S5, we labeled those Cl_2 as Cl_2_x, a new marker species in the model. Cl_2_x follows an identical fate in the model (mainly photolysis) compared with Cl_2. However, the Cl atoms produced by Cl_2_x photolysis do not participate in subsequent reactions. This is because the model constrains the ambient Cl_2 levels, which already accounts for those Cl_2 produced by the NCl_3-related reactions (i.e., Cl_2_x). Therefore, the ratio of Cl_2_x to ambient Cl_2 levels indicates the contribution of NCl_3-related reactions to overall Cl_2 productions. It is noted that the Cl_2 production by NCl_3 photolysis has not been verified by our laboratory study. As the NCl_3-containing air synthesized in the laboratory always contains appreciable proportions (about 20%) of Cl_2, a NCl_3 photolysis experiment is expected to simultaneously photolyze Cl_2, which makes it challenging to isolate the Cl_2 production by NCl_3 photolysis. Another concern is about the safety issue due to the explosive nature of pure NCl_3 (*80*).

1.2 Multiphase chloramine chemistry

The chloramine-related reaction kinetics used in this study are shown in Table S7, which were compiled from previously reported kinetic information of chloramines in aqueous solutions. Here, we show additional technical details on the multiphase chloramine chemistry, including phase transfer processes and aqueous-phase reactions.

Relevant species, e.g., reactive nitrogen oxides (HONO and N_2O_5) and organic aerosols, were also considered.

Phase transfer processes (Pt1~Pt66 in Table S15) encompass both gas-to-aerosol (forward) mass transfer and aerosol-to-gas (backward) mass transfer. In both directions, the rate of change in the concentrations of gaseous and aqueous species was separately simulated in two steps, because these species were expressed in different units (ppb for gases and mol L^{-1} for aqueous species). Mass transfer rates were calculated by the mass transfer constant, k_{mt}, which was a function of temperature, particle size, Henry's law constant, and mass accommodation coefficient (Table S2). We followed the procedure of CAABA/MECCA box model to calculate k_{mt} (*49*).

Aqueous-phase reactions are categorized into four groups (Table S15):

(1) reversible acid dissociation reactions of 34 species (Ad1~Ad68). For example, the dissociation of HOCl (aq) into H^+ (aq) and ClO^- (aq), along with the recombination of H^+ (aq) and ClO^- (aq) to form HOCl (aq), are modeled as two separate reactions. Typically, only an equilibrium constant is reported for reversible acid dissociation reactions, while both forward and backward rate constants must be assigned in the model. In this case, we assume the rate constant of the forward reaction is 10^5 s^{-1}.

(2) aqueous-phase reactions without chloramines (Aq1~Aq280). These irreversible reactions mainly describe the chemical evolution of reactive chlorine species (e.g., Cl_2, HOCl, and $ClNO_2$) in the aqueous phase. The formation and loss of some intermediates are also included, e.g., the formation of Cl_2^- (aq) from the Cl (aq) and Cl^- (aq) reaction. Besides chlorine chemistry, we incorporated reactive nitrogen chemistry that affects reactive chlorine. For example, the hydrolysis of N_2O_5 (aq) produces NO_2^+ (aq), which undergoes branching reactions with Cl^- (aq), NO_2^- (aq), and SO_3^{2-} (aq).

(3) chloramine-related aqueous reactions (CA1~CA38). These reactions describe how NH_2Cl (aq), $NHCl_2$ (aq), and NCl_3 (aq) are produced and consumed in the model. As primary emissions are excluded from the model and currently there is no gas-phase production mechanism of chloramines, our model can generate chloramines solely through these reactions. Consequently, we can evaluate the impact of chloramine chemistry by running the model with and without incorporating this category of reactions. Reactions involving three reactants or more are approximated as two-reactant reactions. For example, the reaction NH2CL_aq + SO32m_aq + (NH4p_aq) = NH3_aq + NH3_aq + CLSO3m_aq (No. CA6 in Table S7) treats NH_4^+ (aq) as a general acid. Literature (*81*) assumes that this reaction involves only two reactants, i.e., NH2CL_aq and SO32m_aq, and the rate constant (1.7×10^2 M^{-2} $s^{-1}\times[NH_4^+]$) depends on the aqueous concentration of NH_4^+ (aq) (unit: M).

(4) aqueous photolysis reactions (Ph1~Ph10). These reactions are primary sources of OH (aq), which is mostly contributed by NO_3^- (aq) photolysis. When Cl_2 (g) and HOCl (g) are not constrained in the model, OH (aq) produced by these photolysis reactions oxidizes Cl^- (aq), ultimately producing Cl_2 (aq) and HOCl (aq), which are ingredients for chloramine production.

Additional notes on the unit and syntax for aqueous-phase reactions are introduced as follows. Units of the rate constants are s^{-1} for reactions involving one reactant, and $mol^{-1}\ L\ s^{-1}$ for reactions involving two reactants. The suffixes of chemical species, i.e., “m” and “p”, mean negative and positive ions, respectively. For example, CLm_aq represents chloride ions in the aqueous phase, while Hp_aq means aqueous hydrogen ions. According to the literature, the species with brackets in Table S7 participate in the reaction but are not accounted for in calculating the rate constants of the reaction. For example, in reaction No. 31, H2O_aq participates in the reaction, but only the concentrations of NCL3_aq and SO32m_aq determine the reaction rate. Those rate constants multiplied by OHm and HCO3m are general base-assisted reactions, while those rate constants multiplied by NH4p and Hp are general acid-assisted reactions. Note that OHm, HCO3m, NH4p, and Hp denote the aqueous phase concentrations of OH^-, HCO_3^-, NH_4^+, and H^+, respectively.

2. Input data

The hourly observations of gas-phase parameters/species and aqueous-phase components were constrained in the model. Gas-phase input included meteorological factors, trace gases, and volatile organic compounds (VOCs), while aqueous/aerosol parameters consist of aerosol surface area density (S_a), liquid water content (LWC), and aqueous-phase concentrations of HOCl, Cl_2, and inorganic ions (e.g., H^+ and Cl^-). Campaign-averaged input and their data sources were shown in Table S13, with detailed explanations of certain input exhibited below.

Field-measured j_{NO2} was used to scale the photolysis frequencies of other photolabile molecules in the model. The model assumes that the ratio of the photolysis frequency between different species remains constant throughout the observation period. While this assumption would cause minor uncertainty for the photolysis frequencies of some species, it represents the best approach when field-measured photolysis frequencies are unavailable.

The S_a under dry conditions was calculated by aerosol particle number size distribution (PNSD), assuming that ambient particles have a spherical shape. Then, a parameterized growth factor (GF) of the particle size was adopted to account for the hygroscopicity of aerosols (*82*). S_a under ambient wet conditions was calculated by multiplying the square of GF and dry S_a. In the model, S_a was used to calculate the rate of heterogeneous reactions, e.g., $ClONO_2$ uptake, to produce HOCl.

The NH_3 and HONO constrained in the model were measured by MARGA at the THU site. Considering the potential uncertainty of MARGA to measure these species, we performed an intercomparison between MARGA and other benchmark instruments to see the magnitude of such uncertainty. We adopted the Picarro instrument (model G2103) to measure NH_3 after our field campaigns at the THU site. Besides, we compared the HONO measured at the THU site to that simultaneously measured by LOPAP at the BUCT site (see Methods for site descriptions). Results showed that MARGA can provide acceptable measurements of NH_3 and HONO compared with

their respective benchmark measurement techniques. In this study, the HONO data was used to constrain the primary production of OH radicals in the model, which did not noticeably affect the production and loss of Cl radicals. On the other hand, the input of NH_3 might affect the box model simulation of chloramines. Nevertheless, as the ambient level of Cl_2 (several ppt) was much lower than that of NH_3 (several ppb), we have demonstrated that the simulated chloramine concentrations depended mainly on Cl_2 levels rather than NH_3 levels. Consequently, we conclude that the uncertainty in NH_3 and HONO measured by MARGA was acceptable and could support our conclusion regarding chloramines.

The C_8H_{10}, C_9H_{12}, and $C_{10}H_{16}$ measured by Vocus-PTR were used to infer the concentrations of aromatic VOCs. We assumed that the measured C_8H_{10} molecules were composed of equal concentrations of *m*-xylene, *o*-xylene, and ethylbenzene. Please note that the F0AM model omitted *p*-xylene. Similarly, we assumed that C_9H_{12} was contributed equally by propylbenzene, *i*-propylbenzene, 1,2,3-trimethylbenzene, 1,2,4-trimethylbenzene, and 1,3,5-trimethylbenzene. Besides, the measured $C_{10}H_{16}$ was regarded as equally contributed by *α*-pinene and *β*-pinene. Sensitivity tests were performed to evaluate the uncertainty for these assumptions, which found no apparent influence on the simulated Cl· budget and chloramine levels.

3. Sensitivity tests

As mentioned in the above section, we could not unambiguously identify the C_8H_{10}, C_9H_{12}, and $C_{10}H_{16}$ molecules measured by Vocus-PTR. So, we performed sensitivity tests to evaluate the uncertainty following the procedure in Xia et al. (2023) (*83*). These tests show that varying the isomer distribution of C_8H_{10}, C_9H_{12}, and $C_{10}H_{16}$ did not cause remarkable differences in the modelled OH· and Cl· levels.

The tests described above were designed to validate the model, while the following sensitivity tests were performed to investigate the influencing factors of the secondary formations of chloramine. We varied the levels of NH_3, Cl_2, RH, and T individually to assess their impacts on chloramine formation. For the sensitivity test of Cl_2 and T, we held other parameters constant in the model. For the RH sensitivity test, we also considered how RH would affect aerosol liquid water content (ALWC) and pH, which were obtained by running the ISORROPIA II model (*23*). After that, we updated the RH along with ALWC and pH in the box model and ran the sensitivity test. For the sensitivity test of NH_3, we assumed that changes in ambient NH_3 levels also caused changes in NH_4^+ concentrations. Correspondingly, SO_4^{2-} and NO_3^- concentrations should also change simultaneously with NH_4^+, as these anions mostly combine with NH_4^+ to form $(NH_4)_2SO_4$ and NH_4NO_3. So, we ran the ISORROPIA II (*23*) model by simultaneously changing the input of NH_3, NH_4^+, SO_4^{2-} and NO_3^- concentrations. Results show that the ALWC and aerosol pH also changed accordingly. Then, we ran the sensitivity test of NH_3 in the box model with updated gas-phase NH_3 and aerosol compositions (including ALWC and pH). Results were discussed in the main text. We did not perform sensitivity tests for aerosol pH alone, as changes in aerosol pH always

accompanied changes in other aerosol-phase species or parameters. Besides, the effect of aerosol pH has been incorporated as a consequence of changes in other factors, e.g., RH and NH_3.

In addition to the above tests of chemical species, we also tested the sensitivity of rate constants of key chloramine-related reactions (Table S14). In detail, we multiplied the rate constants of these reactions by a factor of 2 and simulated the mixing ratio of NCl_3 again. We constrained other reactive chlorines (i.e., Cl_2 and $ClNO_2$) and supporting measurements observed in summer Beijing for the sensitivity test. Results showed that the model simulated NCl_3 was sensitive to some aqueous-phase rate constants (No. 3, 4, 6 in Table S14) while insensitive to those gas-phase reactions in Table S5. As for the intermediate chloramines, NH_2Cl was insensitive to all the tested rate constants, as gaseous NH_2Cl originated from the aqueous phase was released to the gas phase in the very beginning. Additionally, the formation of NH_2Cl was restricted by the availability of Cl_2 (aq) and HOCl (aq). Increasing the rate constant of NH_2Cl productions would correspondingly fasten the exhaust of Cl_2 (aq) and HOCl (aq) but did not cause a net gain of NH_2Cl. The simulated $NHCl_2$ was always near-zero levels, showing complete conversion to NCl_3.

We also conducted additional sensitivity tests to examine how changes in *j*($NHCl_2$ (g)) under the lower- and upper-limit scenarios could affect P(Cl•). Results show that in comparison to the original results, the change of P(Cl•) in Beijing (±1 %) is relatively small since NCl_3 dominates the contribution of chloramine photolysis to P(Cl•). By contrast, due to the relatively high abundance of $NHCl_2$ observed in New Delhi and Toronto, the relative changes in P(Cl•) are -3% to 8% and -10% and 24% under the lower- and upper-limits of *j*($NHCl_2$ (g)) scenarios, respectively, in these two places. As only the photolysis of NCl_3 is considered for the estimation of chloramine contribution to P(Cl•) in other places shown in Fig. 4E, the estimated P(Cl•) would therefore not be largely influenced by the uncertainty of *j*($NHCl_2$ (g)).

Text. S4 Details of the machine learning model

1. Discarding outliers

To minimize the impact of interference factors, outliers were excluded from the dataset. A percentile-based method inspired by John Tukey's Method (*103*), also known as the interquartile range (IQR) method, was implemented to remove outliers in the sample. The outliers were identified using the following formula, where Q_p^j referred to the *p*th percentile of the feature *j*:

$$\text{Outliers} = \left\{x_i \,\middle|\, \exists j, x_{i,j} < Q_{50}^j - r\left(Q_{p_2}^j - Q_{p_1}^j\right) \quad \text{or} \quad x_{i,j} > Q_{50}^j + r\left(Q_{p_2}^j - Q_{p_1}^j\right)\right\} \text{ (Eq. 2)}$$

In this work, p_1 = 5, p_2 = 95, and *r* = 2 were adopted, which were more tolerable than the usual approach (*84*), as we observed high variations in measured NCl_3 such as pronounced spikes in Case 1. A more tolerable setting helps conserving those observations in Case 1. 78 samples were discarded finally.

2. Model construction

Thirty-six common machine learning (ML) regression methods were compared to better predict NCl_3. A 10-fold cross-validation (CV) method was used for optimizing hyper-parameters throughout the model construction, except for the test dataset. Four metrics were used to evaluate model performance, which were coefficient of determination (R^2), Pearson correlation coefficient (Corr), root-mean-square error (RMSE), and mean absolute error (MAE). These four metrics were computed as follows.

$$\mathrm{R}^2 = 1 - \frac{\sum_i (\hat{y}_i - y_i)^2}{\sum_i (\bar{y}_i - y_i)^2} \quad \text{(Eq. 3)}$$

$$\mathrm{Corr} = \frac{\sum_i (\hat{y}_i - \bar{\hat{y}}_i)(y_i - \bar{y}_i)}{\sqrt{\sum_i (\hat{y}_i - \bar{\hat{y}}_i)(\hat{y}_i - \bar{\hat{y}}_i)}\sqrt{\sum_i (y_i - \bar{y}_i)(y_i - \bar{y}_i)}} \quad \text{(Eq. 4)}$$

$$\mathrm{RMSE} = \sqrt{\frac{\sum_i (\hat{y}_i - y_i)^2}{N}} \quad \text{(Eq. 5)}$$

$$\mathrm{MAE} = \frac{\sum_i |\hat{y}_i - y_i|}{N} \quad \text{(Eq. 6)}$$

where $\hat{y}_i$, $\bar{\hat{y}}_i$, y_i, $\bar{y}_i$ refers to the predicted NCl_3 of the *i*th sample, the mean value of predicted NCl_3, measured NCl_3 value of the *i*th sample, the mean value of measured NCl_3, respectively, and *N* refers to the number of samples.

Due to the vast amount of work involved in hyper-parameter optimization, a two-stage model selection approach was implemented. In the first stage, the default hyper-parameters in scikit-learn were used for model training and validation (results were shown in Table S11). The top five models in the first stage were selected for further optimization. In the second stage, the hyper-parameters for each of the five models were optimized, and the results were presented in Table S12. Subsequently, a feature selection process utilizing a Greedy algorithm was meticulously executed to refine the model performance. The ML models were developed within a Python environment, powered by the “scikit-learn”, “lazypredict”, and “shap” packages.

3. Explanation on feature selection rationale

In this study, machine learning was employed to complement the knowledge-driven field observation and chemical box modelling analyses, offering a relatively objective perspective to enhance the interpretation of complex atmospheric processes. The selected features were largely based on mathematical efficiency rather than physical implications.

For instance, ALWC might have a more direct connection with aqueous-phase chemistry than RH. However, when we replaced RH with ALWC and retrained the XGBoost model, the model performance became worse. On test dataset, the R^2 dropped

from 0.83 to 0.67. The decrease in the R^2 can be explained by data distribution and outlier processing. As shown in Fig. S25A-B, RH presented a relatively uniform distribution, while ALWC mostly concentrated in a narrow range (< 50 μg m^{-3}). In addition, numerous data points of ALWC were recognized as outliers and removed from the dataset (Fig. S25C), which reduced the diversity of the data. As for the SHAP value results, the relative importance and mean absolute SHAP value of the influencing factors almost all remained unchanged except for the importance of water, dropping from 0.36 using RH to 0.10 using ALWC (Fig. S25D). After all, an overall positive relationship between RH and ALWC was observed. Considering the mathematical accuracy of the model performance, we prefer to use RH rather than ALWC as an input in our machine learning model.

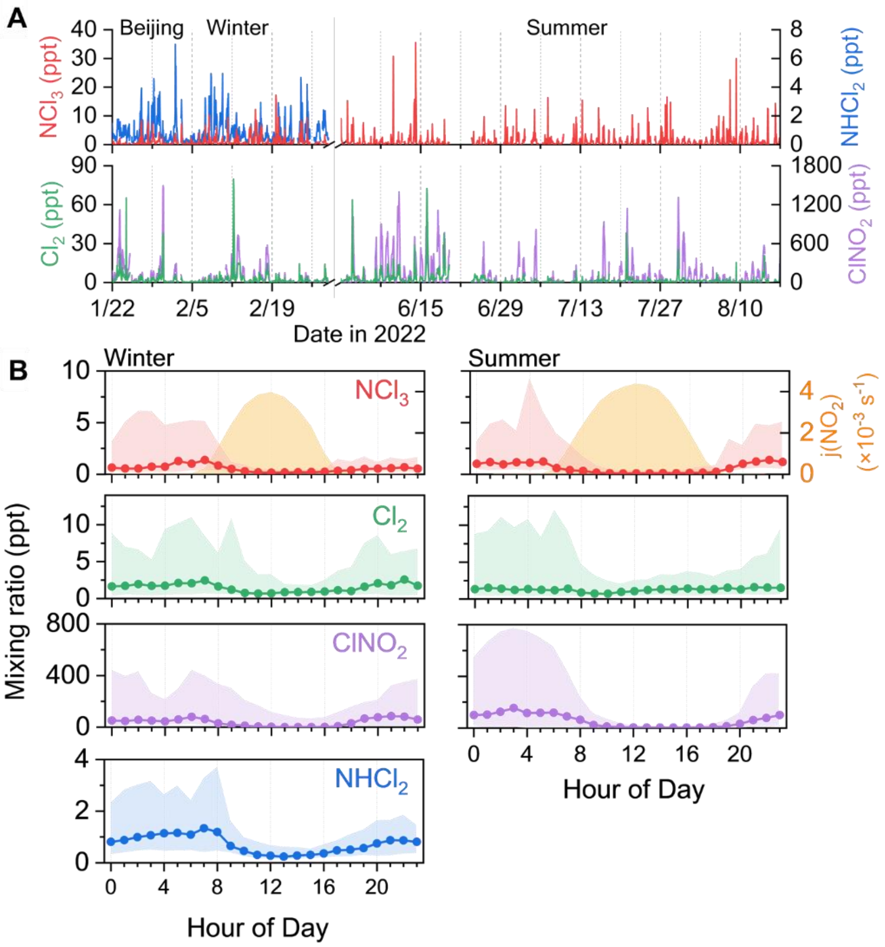

Fig. S1 Field measurements of reactive chlorines in winter and summer Beijing. (A) Time series and (B) diurnal patterns. The solid dots represent the median values, and the shaded areas denote the 10th and 90th percentiles. The filled area of j(NO_2) indicates the measured average NO_2 photolysis frequency.

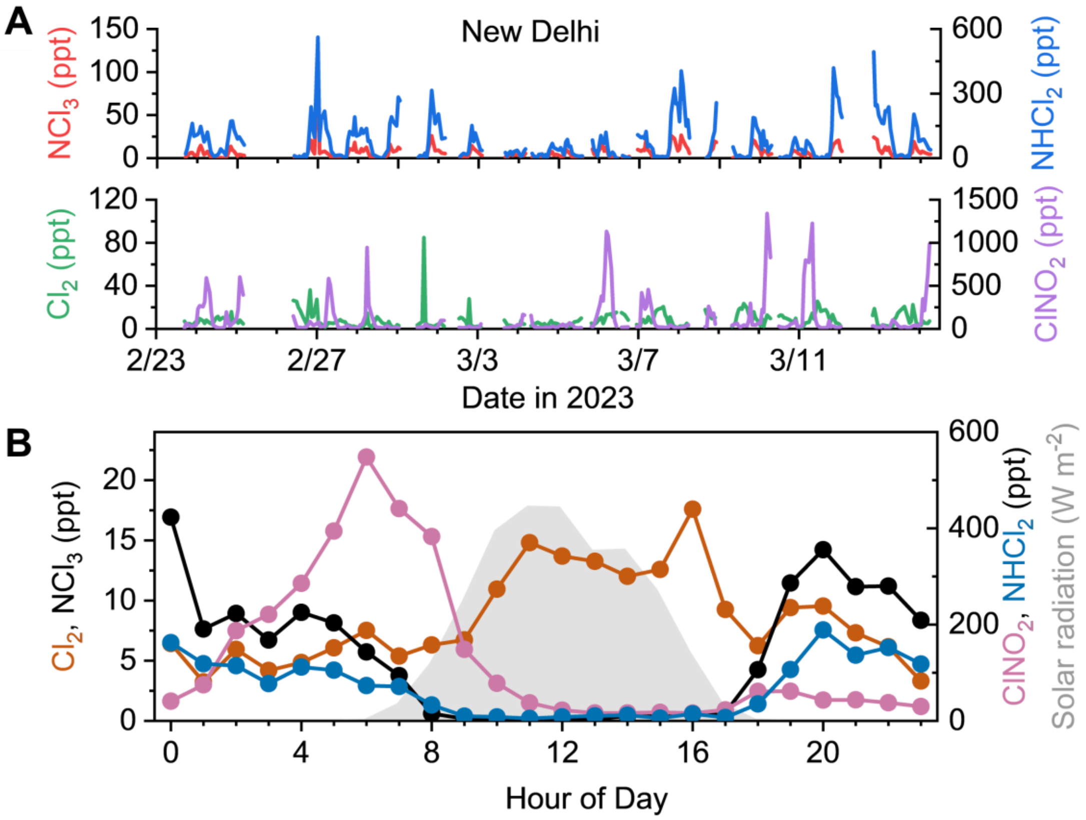

Fig. S2 Field measurements of reactive chlorines in New Delhi. (A) Time series and (B) average diurnal variations.

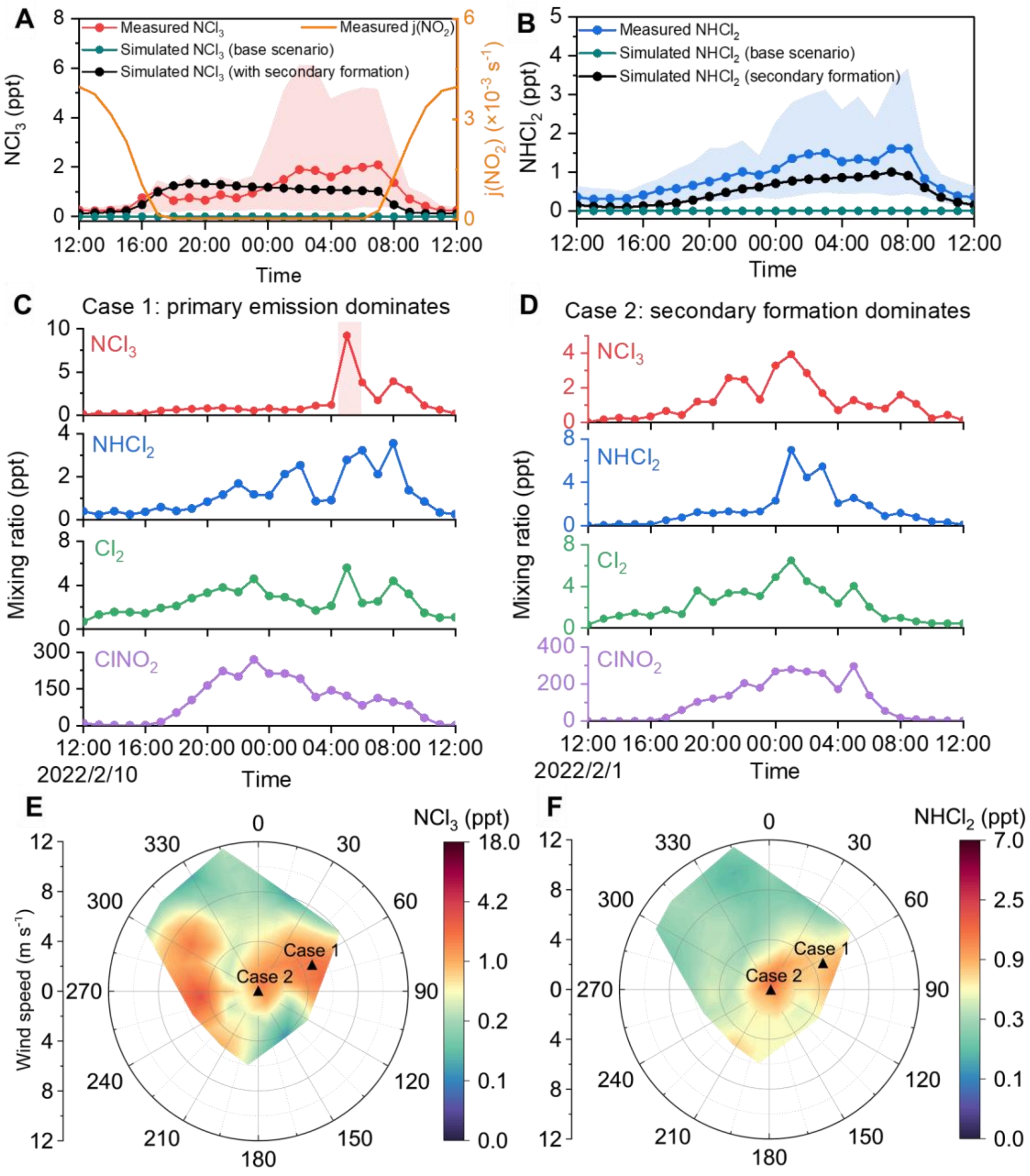

Fig. S3 Field characterization of primary and secondary productions of NCl_3 in winter Beijing. Secondary production of NCl_3 in winter Beijing. Comparison of the measured and simulated daily averages of (A) NCl_3 and (B) $NHCl_2$ mixing ratios in winter Beijing. The shaded area represents the 10th and 90th percentiles of measured NCl_3 and $NHCl_2$. The orange line in (A) denotes the measured average diurnal variation in NO_2 photolysis frequency. A case indicates (C) primary emission and (D) secondary production dominates the observed NCl_3. Wind rose plots color-coded by (E) NCl_3 and (F) $NHCl_2$ mixing ratios, respectively. Cases shown in (C) and (D) were marked as triangles in (E) and (F) accordingly.

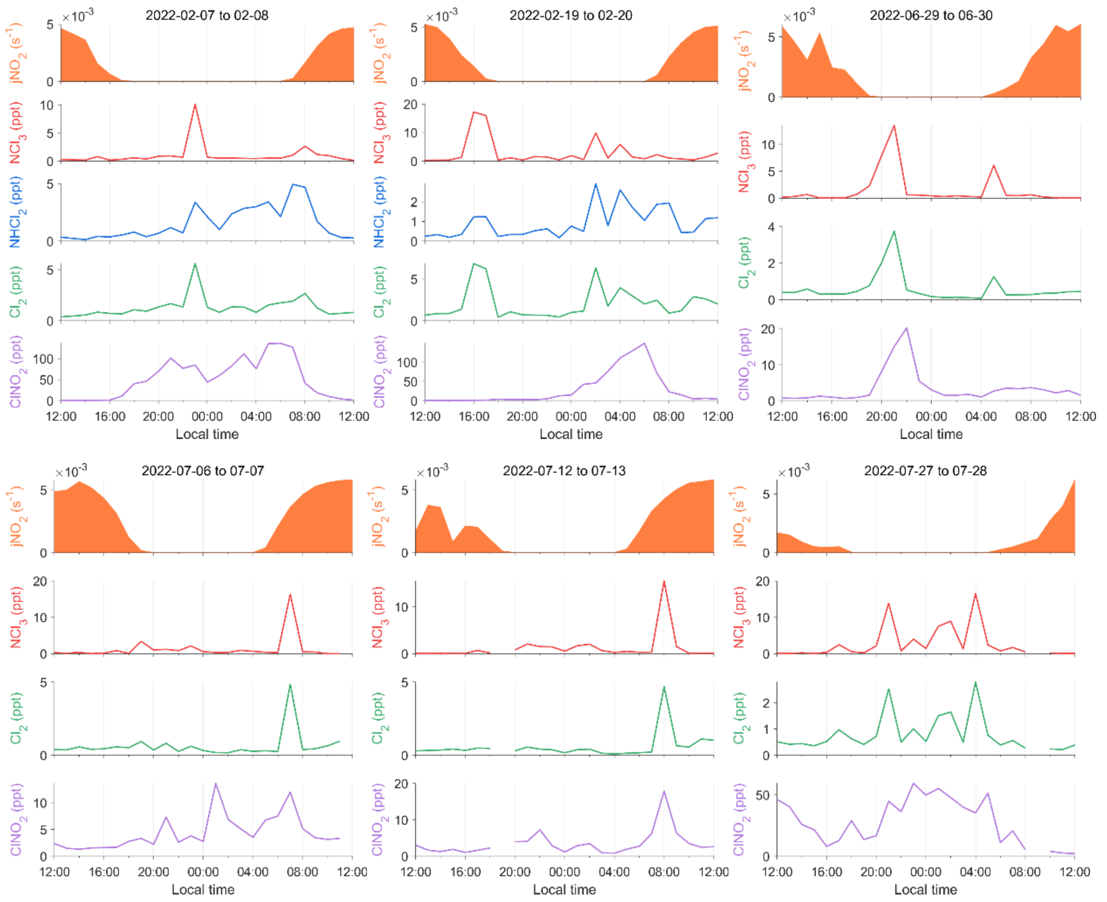

Fig. S4 Typical cases indicating primary emission of NCl_3 in Beijing. Hourly-averaged j_{NO2}, NCl_3, Cl_2, and $ClNO_2$ mixing ratios are shown in each figure, with the measurement dates indicated in the title, e.g., 2022-07-27 to 07-28 means 2022-07-27 12:00 to 07-28 12:00.

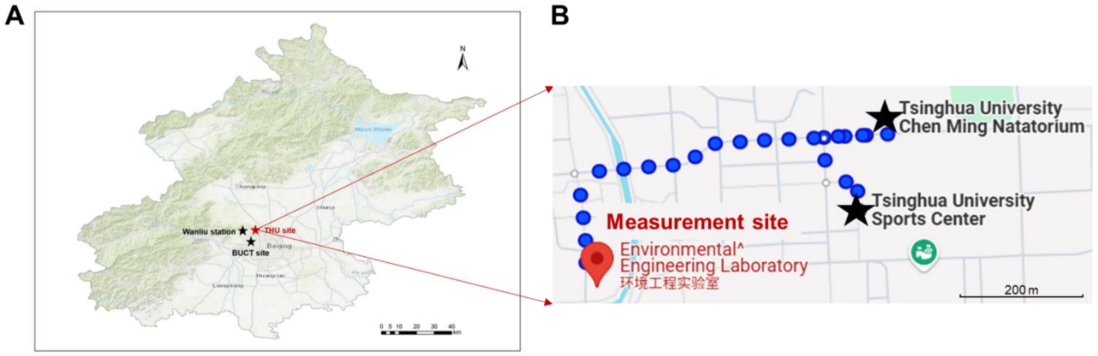

Fig. S5 Locations of the measurement site. (A) Locations of the THU site, BUCT site, and Wanliu station in Beijing, and (B) a zoom-in view of the THU sampling site. The positions of the nearby indoor swimming pool and sports center are marked as asterisks.

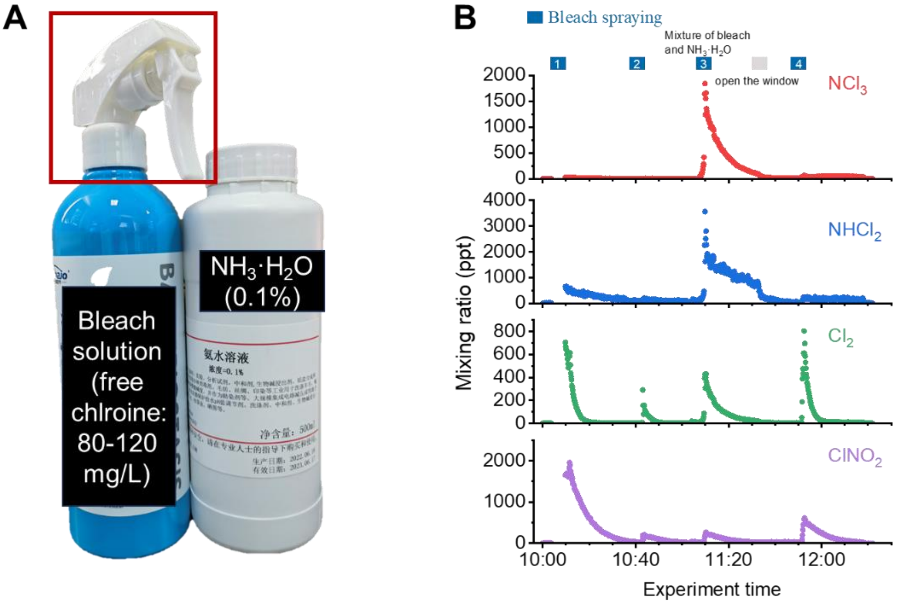

Fig. S6 The bleach spraying experiments. (A) A photo of the used reagents, (B) variations of reactive chlorines during the experiment. Substantial increases of NCl_3 and $NHCl_2$ were observed when mixing trace amounts of $NH_3 \cdot H_2O$ (residues on the inner wall of the beaker) and ~100 ml bleach solution.

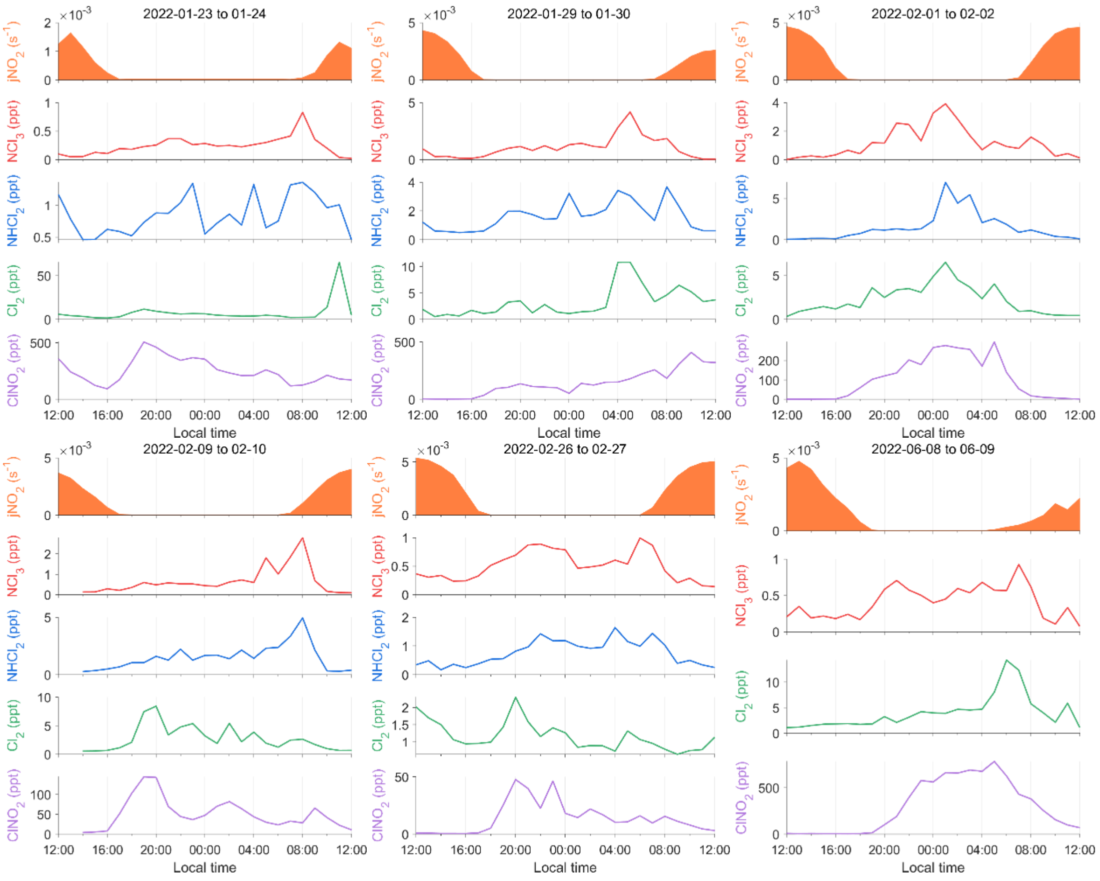

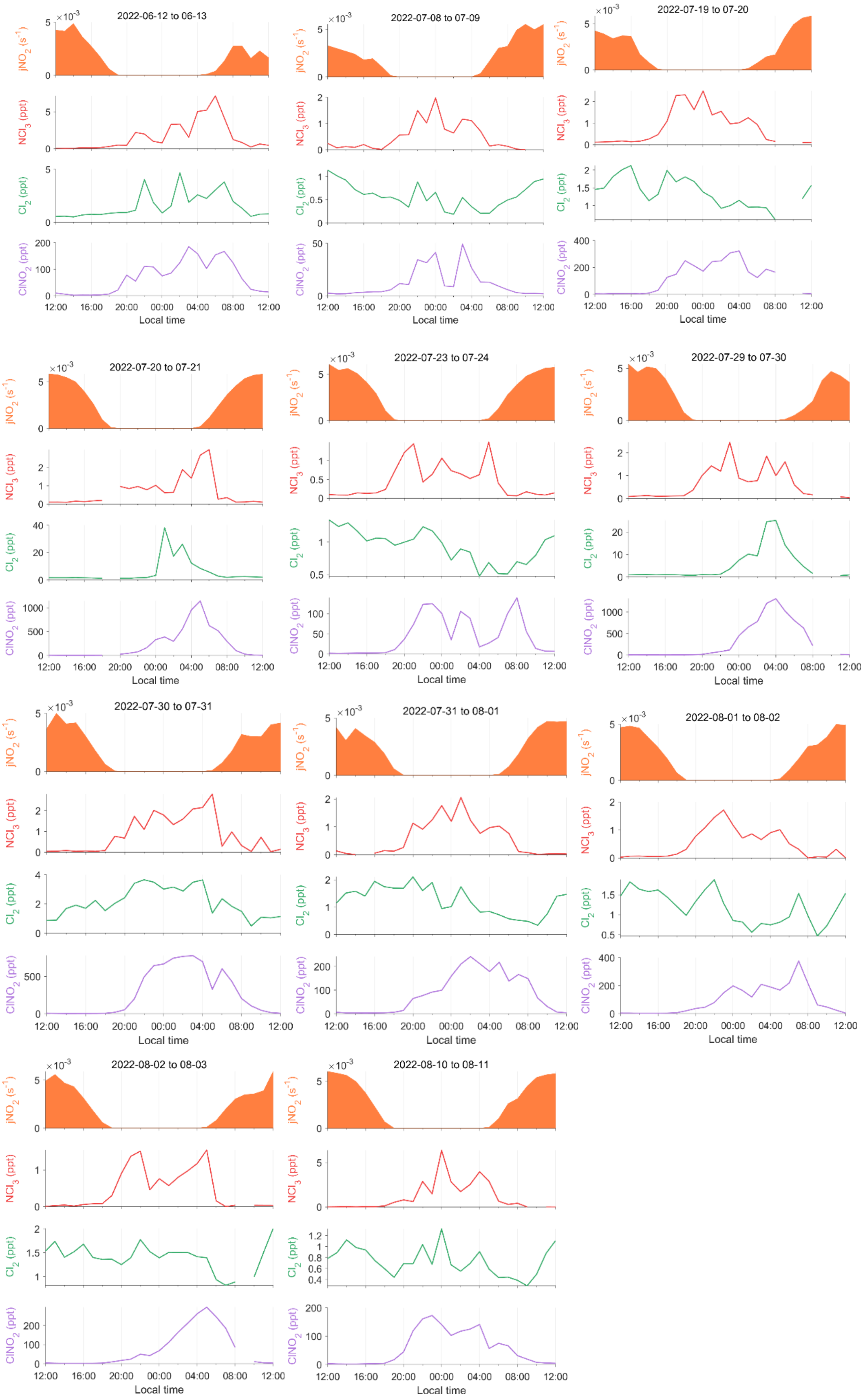

Fig. S7 Typical cases indicating secondary production of NCl_3 in Beijing. Hourly-averaged j_{NO2}, NCl_3, Cl_2, and $ClNO_2$ mixing ratios are shown in each figure, with the

measurement dates indicated in the title, e.g., 2022-08-10 to 08-11 means 2022-08-10 12:00 to 08-11 12:00.

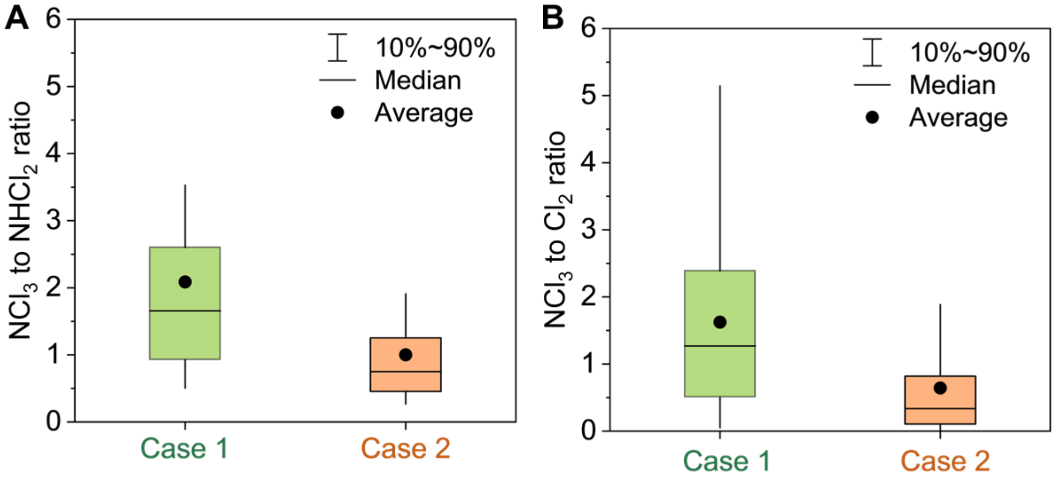

Fig. S8 Comparison of Case 1 and Case 2 during the field campaign in Beijing. Box plots of (A) NCl_3 to $NHCl_2$ and (B) NCl_3 to Cl_2 ratios. The upper and lower edges of the box represent 25th and 75th percentiles, respectively. The whiskers denote the 10th and 90th percentiles.

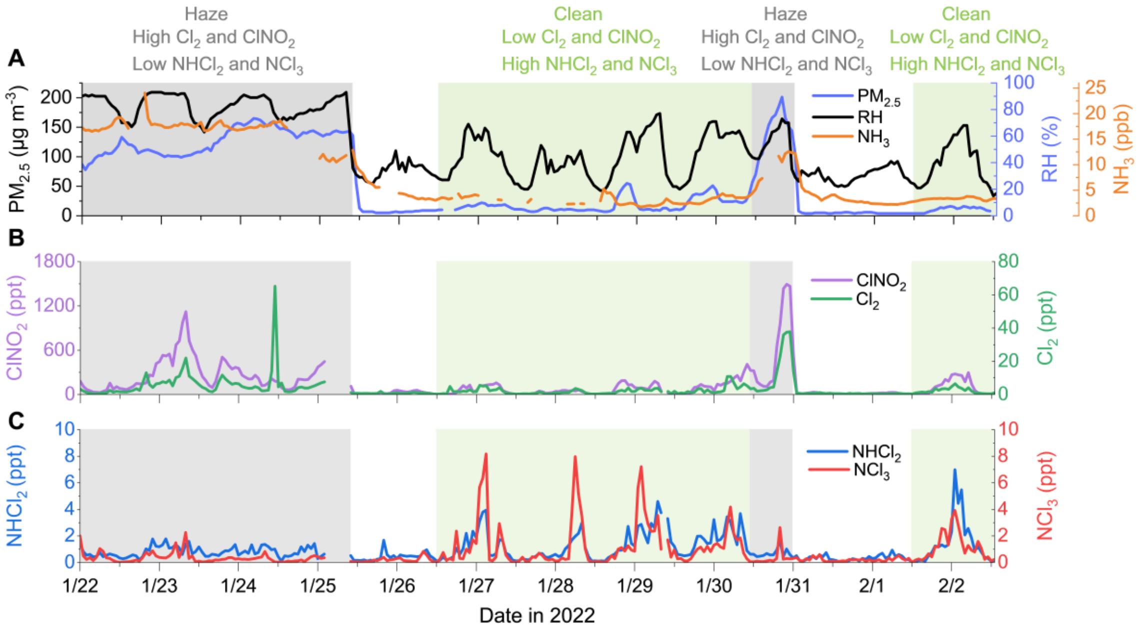

Fig. S9 Characteristics of reactive chlorines during haze and clean periods in winter. Time series of (A) $PM_{2.5}$, relative humidity (RH) and NH_3, (B) $ClNO_2$ and Cl_2, and (C) $NHCl_2$ and NCl_3. The haze and clean periods are shaded in gray and green, respectively.

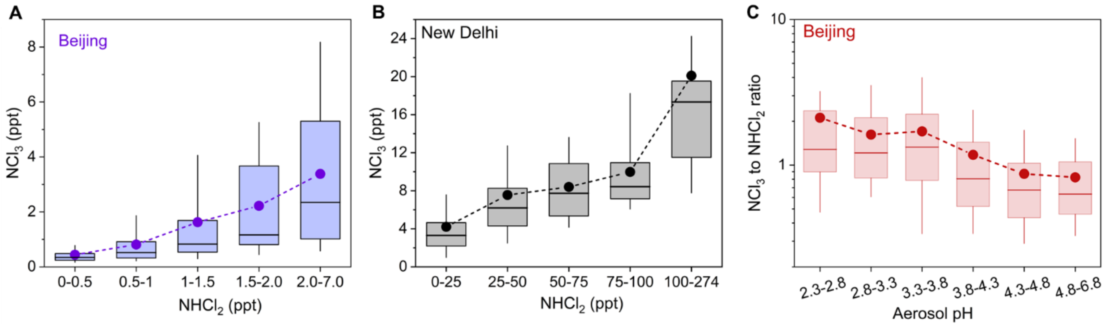

Fig. S10 Relationship between nocturnal $NHCl_2$ and NCl_3 mixing ratios. (A) Beijing, (B) New Delhi, and (C) dependency of nocturnal NCl_3 to $NHCl_2$ ratio and binned aerosol pH in Beijing.

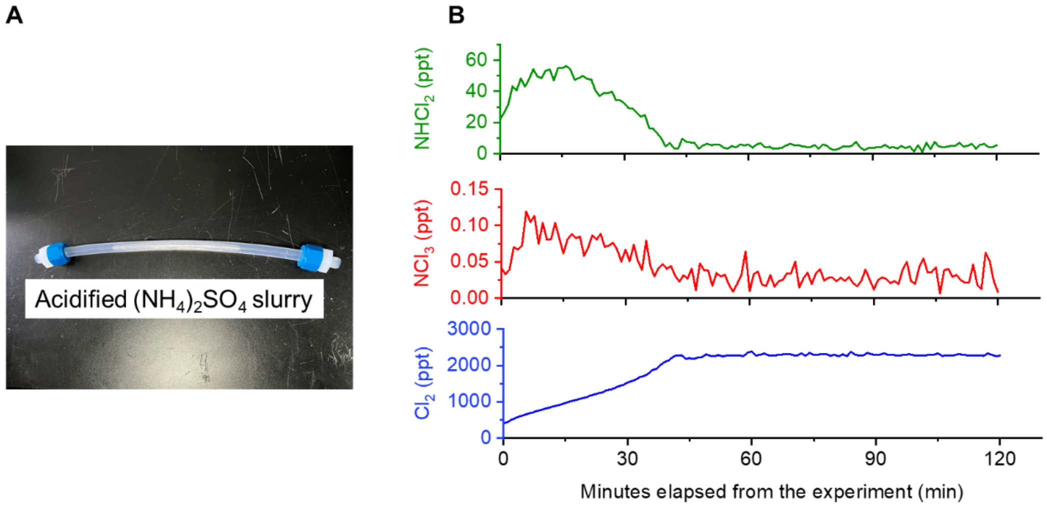

Fig. S11 The experiments designed to simulate secondary chloramine production. (A) The photo of the acidified $(NH_4)_2SO_4$ slurry, and (B) the variations of NCl_3, $NHCl_2$, and Cl_2 mixing ratios when passing Cl_2 through the slurry.

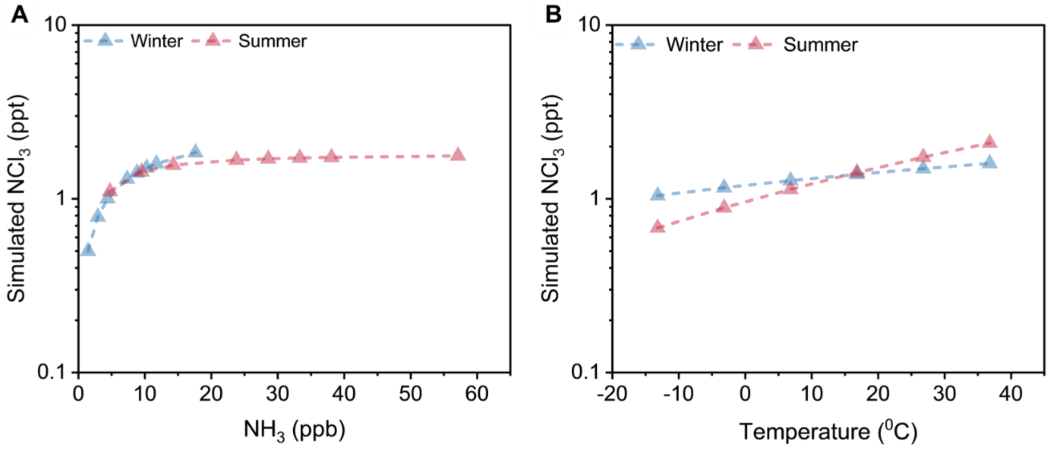

Fig. S12 Box model sensitivity tests. Variations of the simulated NCl_3 with the change of nocturnal (A) NH_3 and (B) temperature in Beijing. Other inputs besides NH_3 or temperature are constrained by the measurements.

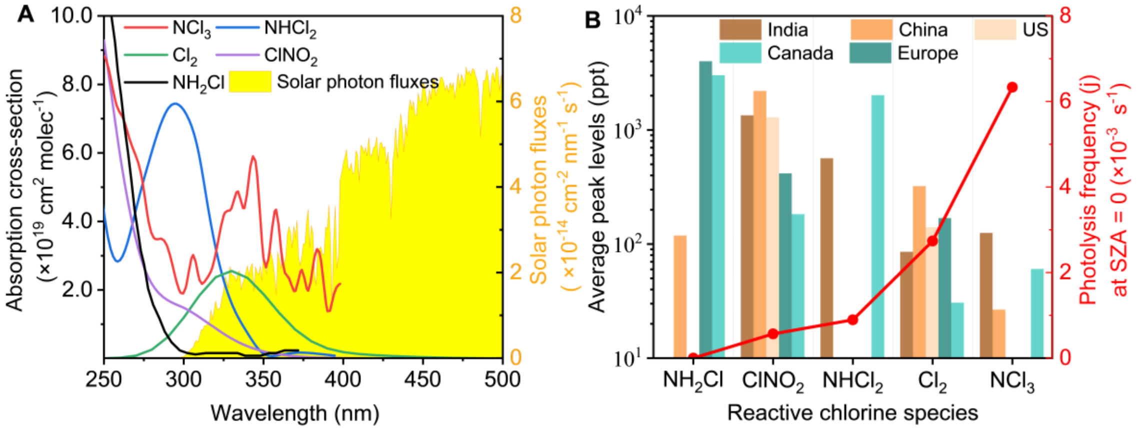

Fig. S13 Comparison of the photolysis frequency and abundances of reactive chlorines. (A) Absorption cross-sections (*39*, *75*, *76*, *85*) and (B) mixing ratios of reactive chlorines observed in different regions. The absorption cross-section for gaseous $NHCl_2$ and NH_2Cl is estimated from their molar absorptivity in aqueous solutions. The photolysis frequency shown in (B) is calculated at solar zenith angle (SZA) of 0. The average peak levels in (B) are averaged from field measurements in the US (e.g., (*8*, *9*)), China (e.g., (*22*, *60*)) (this study included), Canada (e.g., (*12*, *56*)), Europe (e.g., (*11*, *58*)), India (*73*) (this study included).

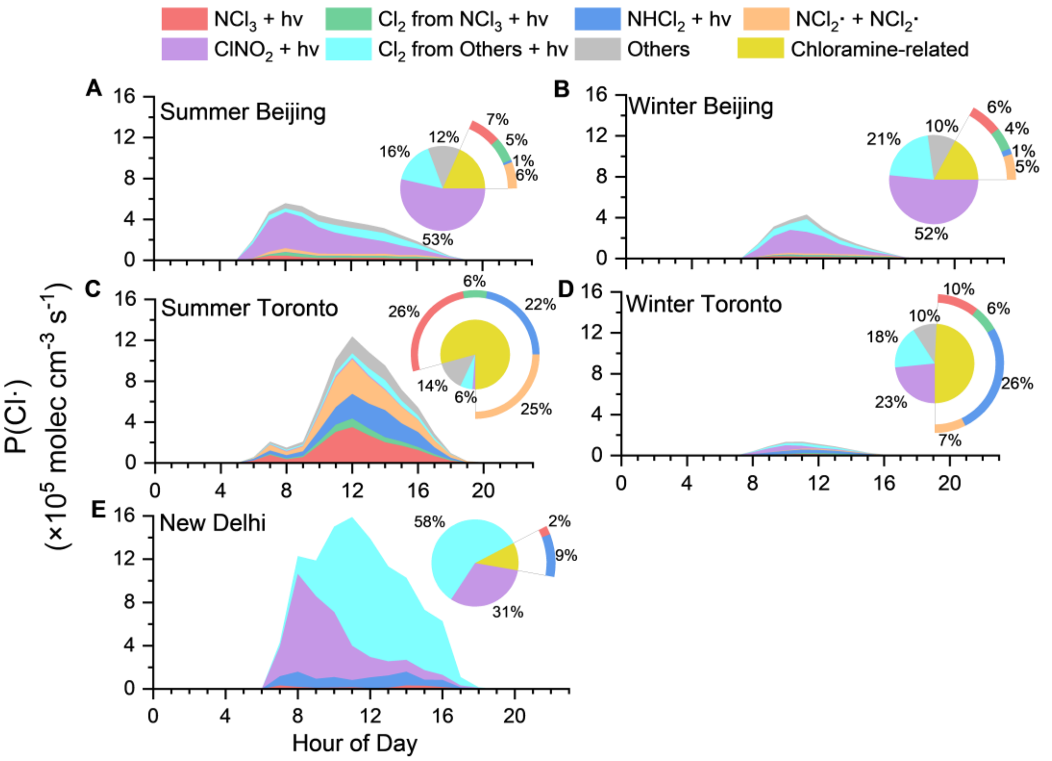

Fig. S14 Average diurnal contributions of reactive chlorines to Cl· production rate (P(Cl·)). In (A) summer and (B) winter Beijing, (C) summer and (D) winter Toronto (*12*), and (E) New Delhi. "Cl_2 from others" means the proportion of Cl_2 that is produced by other pathways except for chloramine chemistry. "Others" means the combination of all other trivial Cl· production pathways (e.g., ClO· + NO) which have smaller

contributions to P(Cl·). The inserted pie charts illustrate the campaign-averaged contributions from different pathways to P(Cl·).

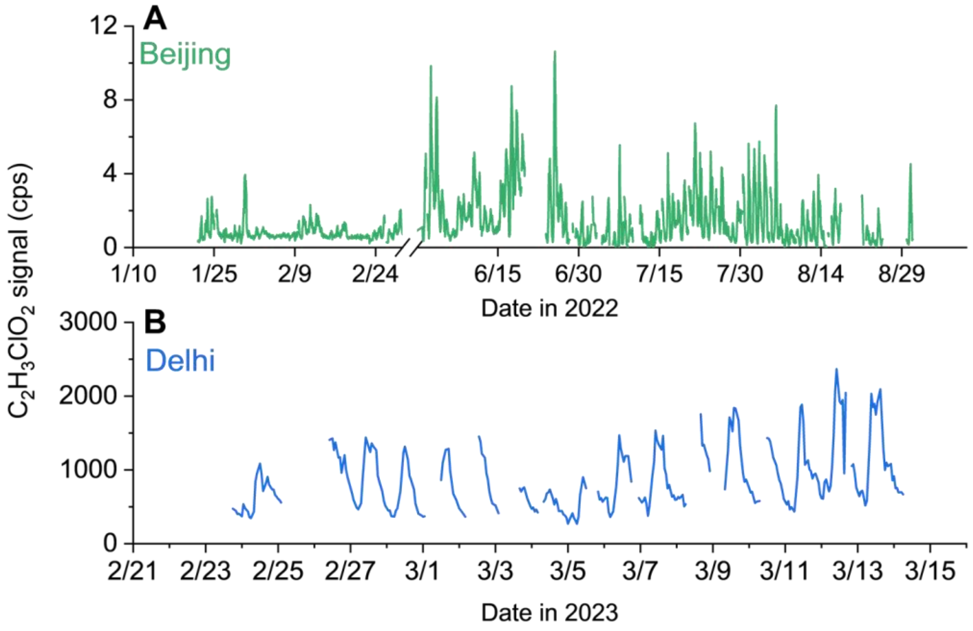

Fig. S15 Time series of $C_2H_3ClO_2$ (presumably chloroacetic acid) signals. During the campaign in (A) Beijing and (B) New Delhi, respectively. The data points are averaged into a 1-hour interval.

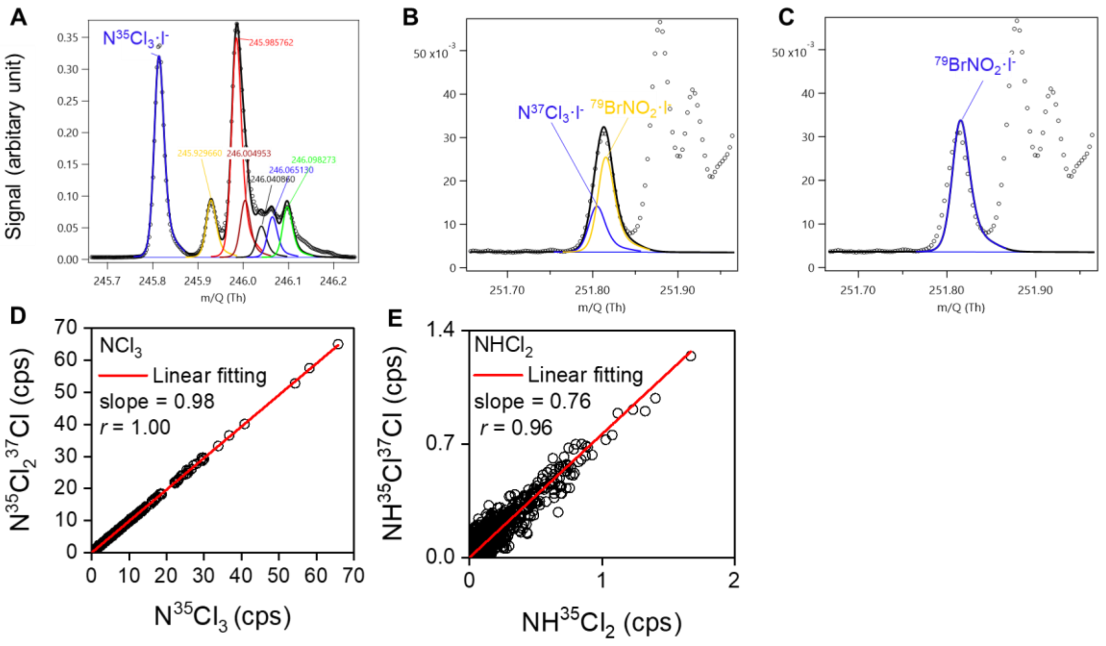

Fig. S16 Peak identification of chloramines. Peak fitting results of (A) NCl_3 and $BrNO_2$ (B) with and (C) without NCl_3 being identified. The open circles and solid lines indicate the measured and fitted signals, respectively. The fitted signal of a potential

Br· precursor, nitryl bromide ($BrNO_2$), is better constrained with NCl_3 being identified. Measured isotope correlations between (D) NCl_3 and (E) $NHCl_2$.

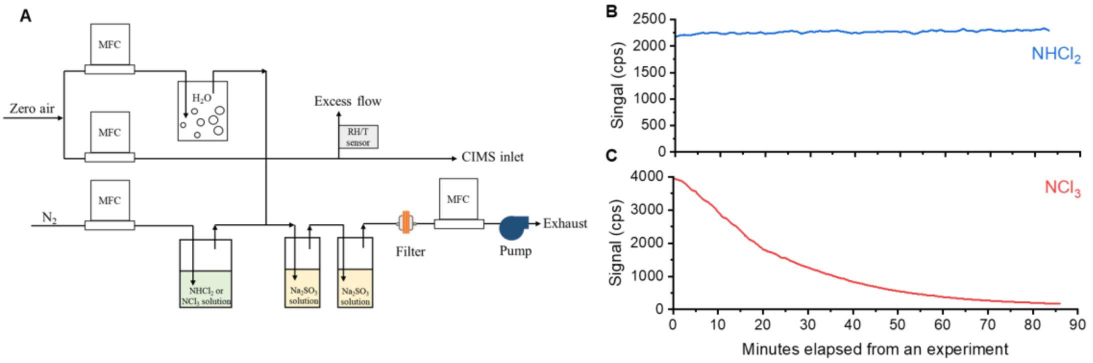

Fig. S17 Calibration of $NHCl_2$ and NCl_3 sensitivities. (A) calibration set-up, and signals of (B) $NHCl_2$ and (C) NCl_3 during the calibration. MFC indicates mass flow controller meters.

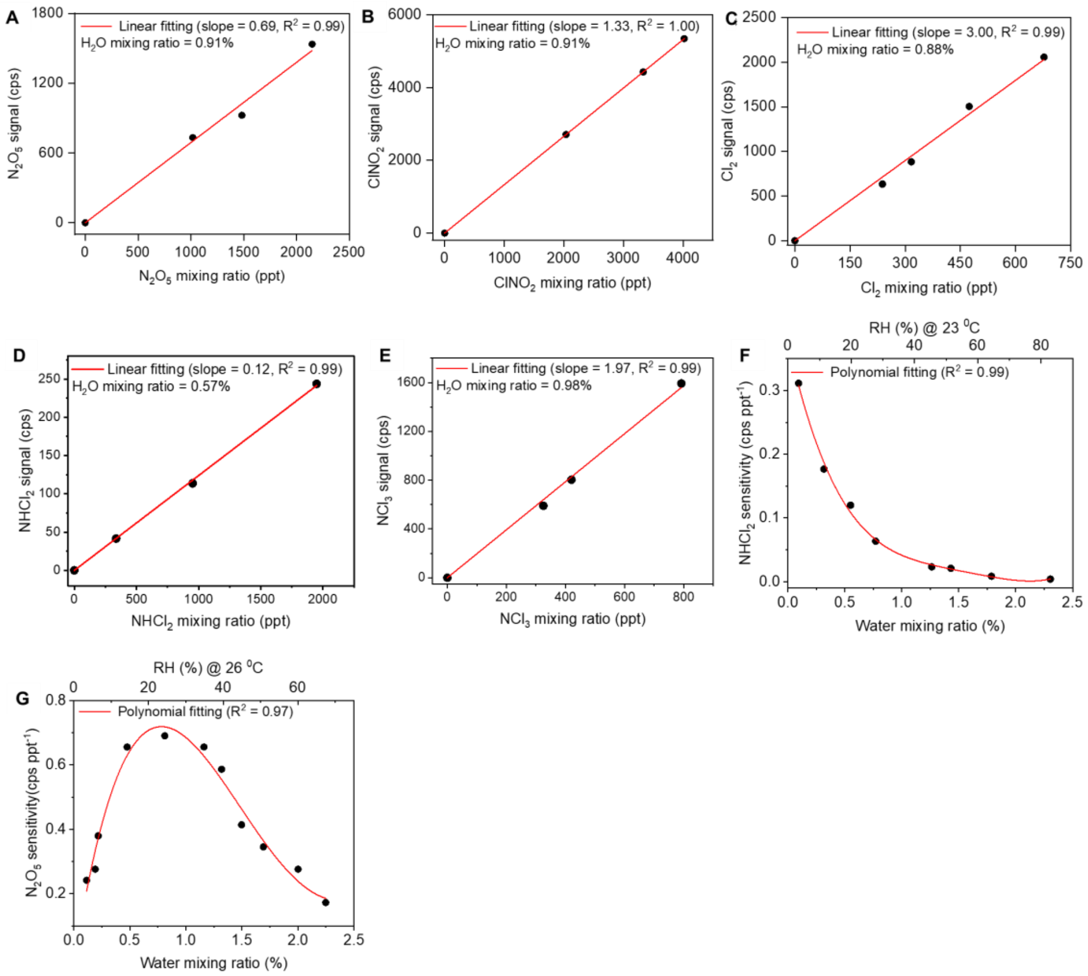

Fig. S18 Calibrations of reactive chlorines and N_2O_5. Sensitivity of (A) N_2O_5, (B) $ClNO_2$, (C) Cl_2, (D) $NHCl_2$, (E) NCl_3. (F) and (G) show the sensitivity dependency of $NHCl_2$ and N_2O_5 on water mixing ratio, respectively.

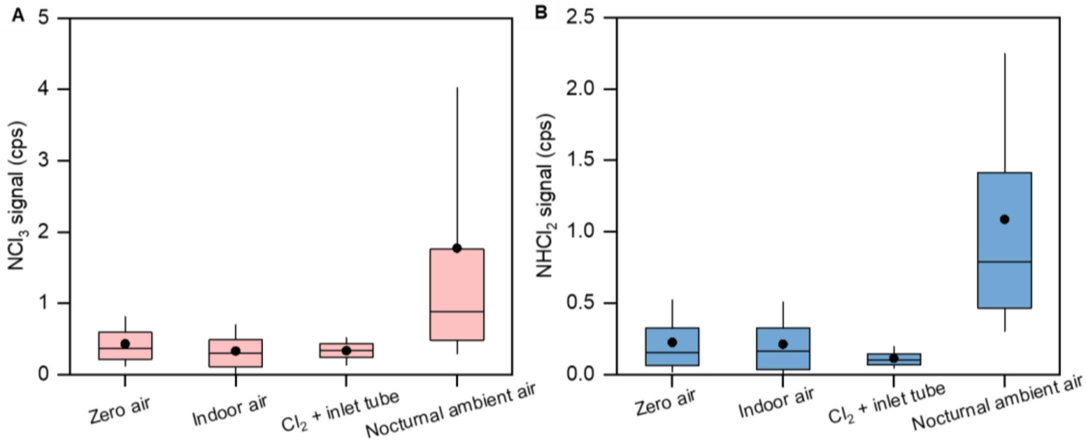

Fig. S19 Summary of chloramine measurement artifact tests. Comparison of (A) NCl_3 and (B) $NHCl_2$ signals when injecting dry zero air, indoor air, passing humidified Cl_2 through a used inlet tube, and sampling the nocturnal ambient air. The bottom and top edges of the box represent the 25th and 75th percentiles, respectively. The black lines and circles inside the box denote the median and average values, respectively. The whiskers indicate the 10th to 90th range. The indoor air sampling was conducted from 14:50 to 17:50 Feb 27th in winter and 23:30 Aug 14th to 9:00 Aug 15th in summer of 2022.

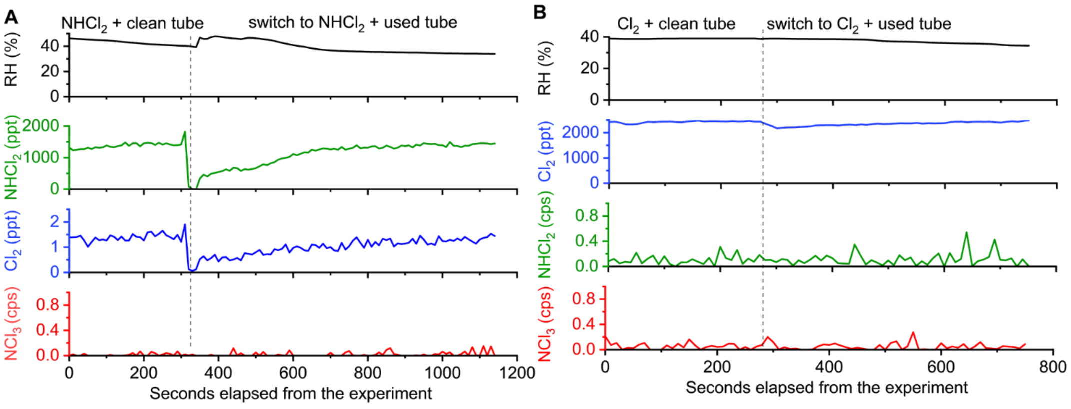

Fig. S20 Inlet artifact tests of chloramine measurements. Comparison of passing (A) $NHCl_2$ or (B) Cl_2 through a clean or used tube. The data points are averaged to 10-s intervals. The tests were performed on July 2nd, 2022 during the campaign in Beijing.

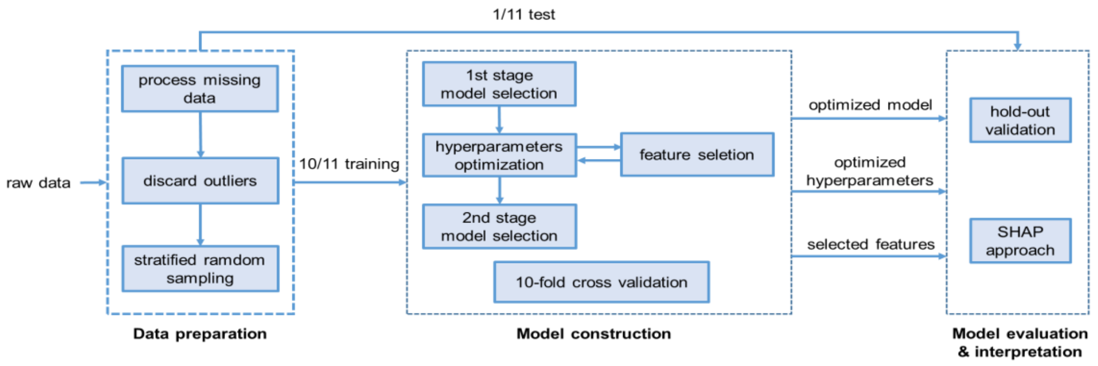

Fig. S21 The framework of the machine learning model.

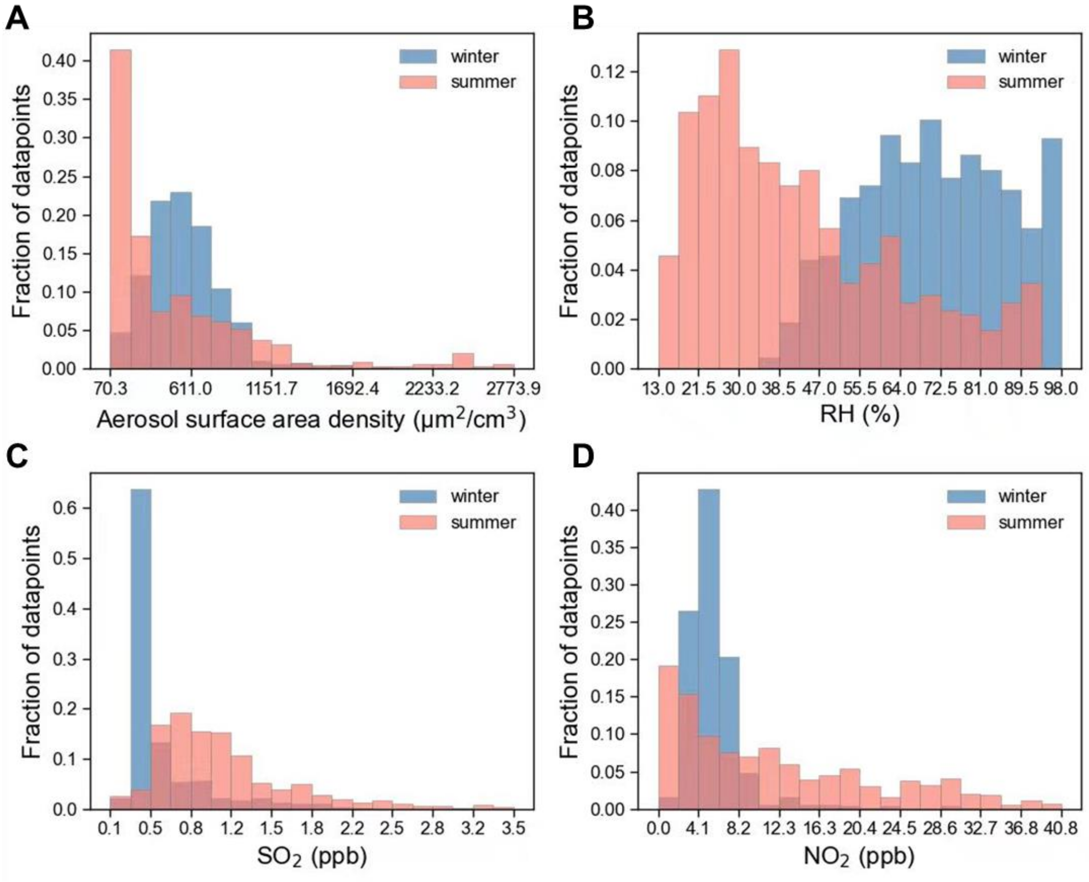

Fig. S22 Data distribution comparison of observational parameters in winter and summer Beijing. (A) aerosol surface area density, (B) RH, (C) SO_2, and (D) NO_2.

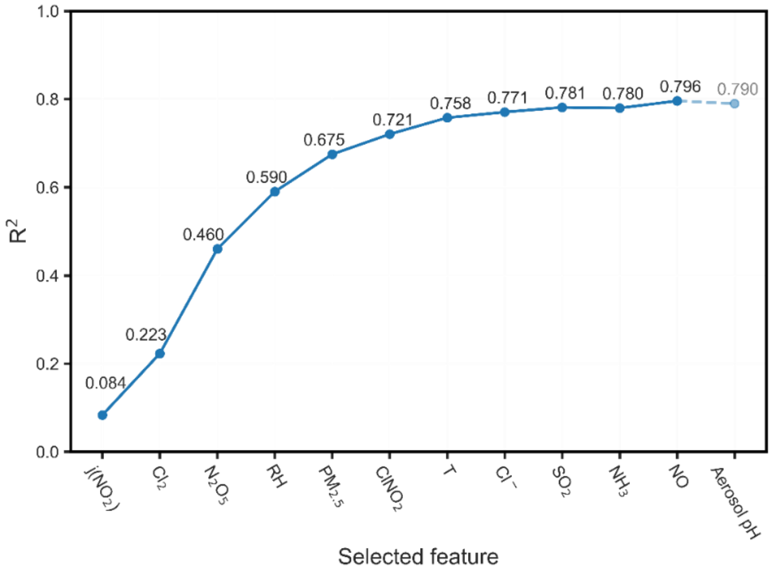

Fig. S23 R^2 variation with feature addition based on the greedy algorithm.

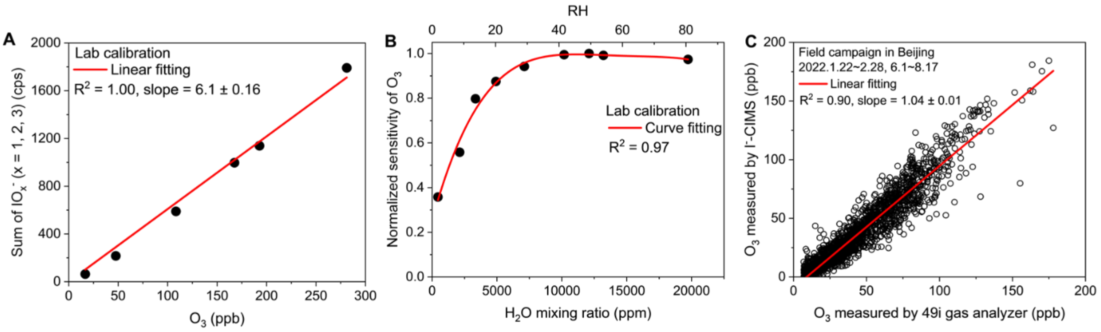

Fig. S24 Comparison of O_3 measured by I^--CIMS and gas analyzer. (A) Laboratory calibration of O_3 in Beijing at RH=45%. Strong linearity ($R^2 \geq 0.99$) was also observed at RH=1%, 14%, and 80% (figures not shown). (B) RH dependency of O_3 sensitivity, which was normalized to the maximum value. (C) Comparison of hourly O_3 mixing ratios measured by I^--CIMS and 49i gas analyzer during the field campaign in Beijing.

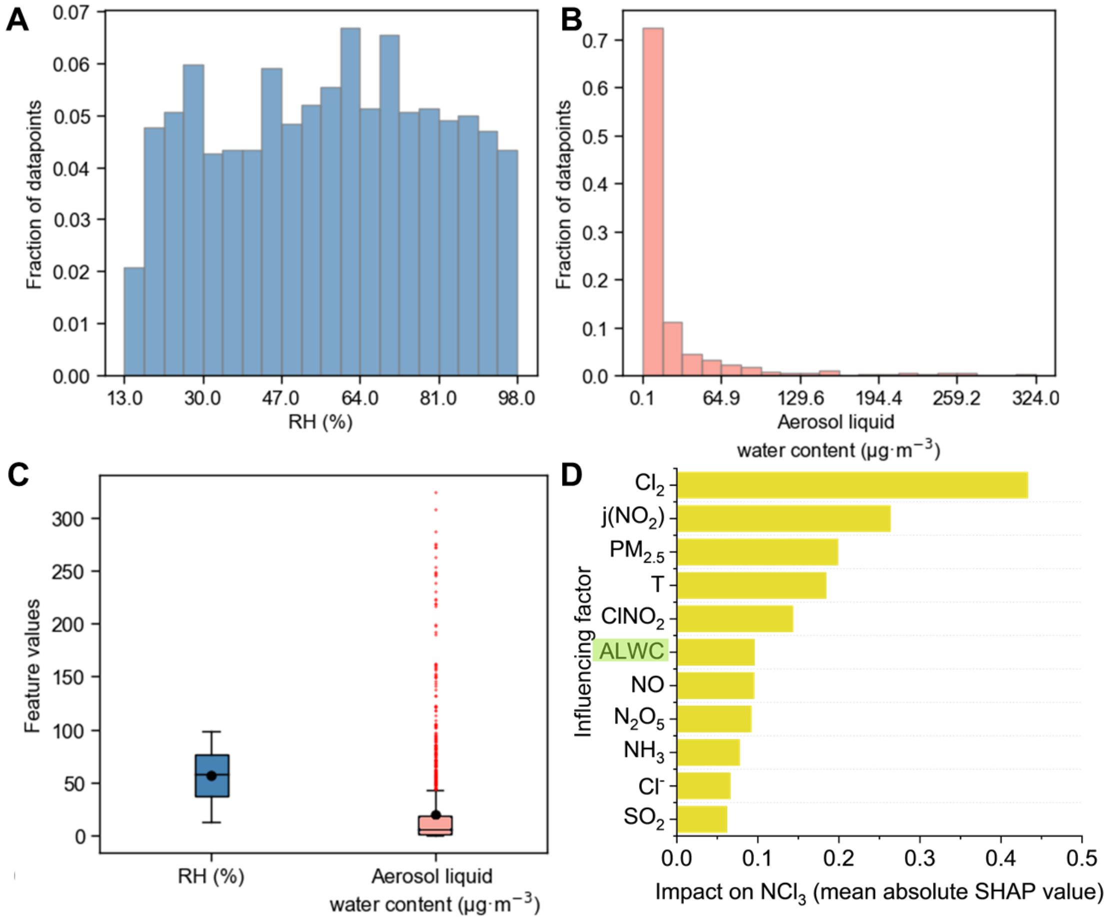

Fig. S25 Comparison of RH and ALWC observed in Beijing. Frequency distribution of (A) RH and (B) ALWC. Box plots of (C) RH and ALWC. (D) Mean absolute SHAP value of different factors ranked by their overall importance when replacing RH with ALWC.

Table S1 Summary of peak chloramine levels and related inorganic chlorinated species in indoor and outdoor observations (unit: ppb).

Location		Method	NH_2Cl	$NHCl_2$	NCl_3	Cl_2	HOCl	$ClNO_2$	Ref.
Indoor: room floor mopping with bleach		I^--CIMS		5×10^{-3} cps	4×10^{-2} cps	25	250	1.5×10^{-2} cps	(*18*)
Indoor: room floor mopping with bleach		PTR and I^--CIMS	60	0.5	7	130	370	34	(*17*)
Indoor: equipment dichlor solution spraying		PTR and I^--CIMS	9 cps	650 cps	3200 cps	500 cps	200 cps	300 cps	(*86*)
Indoor: equipment dichlor solution spraying		I^--CIMS		1500 cps	7500 cps	0.13	400 cps	1.1	(*87*)
Indoor: swimming pool		online derived CRDS			226				(*15*)
Indoor: swimming pool		Colorimetric analysis			204				(*88*)
Indoor: commercial kitchen bleach cleaning		PTR	15						(*89*)
Indoor: bleach spraying in a chamber		PTR and I^--CIMS	1.5	1.0*		80	800	70	(*90*)
Indoor: bathroom bleach cleaning		CRDS					220		(*91*)
Indoor: factories processing vegetables		Potentiometry and Ion Chromatography	1319 (NH_2Cl + $NHCl_2$)		470				(*16*)
Indoor: bleach spraying		I^--CIMS		4	2	0.8		2	this study
Outdoor: University of Leicester (urban background)	summer	I^--CIMS	2.2	BDL	BDL	0.22			(*11*)
	winter	I^--CIMS	4.0	BDL	BDL	0.32			
Outdoor: University of Toronto (urban downtown)	summer	I^--CIMS	6	4	0.1	0.006		0.08	(*12*)
	winter	I^--CIMS	0.03	0.025	0.014	0.02		0.3	
Outdoor: California Institute of Technology		PTR	observed						(*92*)
Outdoor: Tsinghua University	summer	PTR and I^--CIMS	0.17	BDL	0.036	0.072		1.4	this study
Outdoor: Tsinghua University	winter	PTR and I^--CIMS	0.07	0.007	0.017	0.080		1.5	
Outdoor: India Institute of Technology Delhi		I^--CIMS		0.562	0.124	0.085		1.3	

Note: (1) Except for NH_2Cl, the recorded mixing ratios are measured by I-CIMS for the other chlorinated species unless otherwise noted.

(2) *measured by PTR. (3) CRDS is the abbreviation for cavity ring-down spectroscopy, BDL indicates below detection limit.

Table S2 Henry's law constants and mass accommodation coefficients used in the box model.

Species	H^{cp} at T^{Θ}(M/atm)	$dlnH^{cp}/d(1/T)$	α^{Θ}	$dln[\alpha/(1-\alpha)]/d(1/T)$
O_3	1.03×10^{-2}	2.83×10^{3}	2.00×10^{-3}	0
OH	3.00×10^{1}	4.30×10^{3}	1.00×10^{-2}	0
HO_2	3.90×10^{3}	5.90×10^{3}	5.00×10^{-1}	0
H_2O_2	1.10×10^{5}	6.34×10^{3}	7.70×10^{-2}	3127
NO_2	1.20×10^{-2}	2.36×10^{3}	6.30×10^{-4}	0
NO_3	3.80×10^{-2}	2.00×10^{3}	4.00×10^{-2}	0
NH_3	6.02×10^{1}	4.16×10^{3}	6.00×10^{-2}	0
HONO	4.90×10^{1}	4.78×10^{3}	4.00×10^{-2}	0
HNO_3	2.60×10^{6}	8.69×10^{3}	5.00×10^{-1}	0
HNO_4	1.26×10^{4}	6.90×10^{3}	1.00×10^{-1}	0
N_2O_5	2.10	3.40×10^{3}	1.00×10^{-1}	0
CO_2	3.40×10^{-2}	2.40×10^{3}	5.00×10^{-5}	2000
HCHO	3.20×10^{3}	7.10×10^{3}	4.00×10^{-2}	0
CH_3O_2	6.00	5.60×10^{3}	1.00×10^{-2}	2000
HCOOH	8.90×10^{3}	6.10×10^{3}	1.40×10^{-2}	3978
$HOCH_2O_2$	8.00×10^{4}	8.20×10^{3}	1.00×10^{-1}	0
CH_3OOH	3.00×10^{2}	5.32×10^{3}	4.60×10^{-3}	3273
CH_3OH	2.20×10^{2}	5.20×10^{3}	1.00×10^{-1}	0
$HOCH_2OH$	1.00×10^{4}	9.87×10^{3}	1.00×10^{-1}	0
$HOCH_2OOH$	1.70×10^{6}	9.87×10^{3}	1.00×10^{-1}	0
CH_3NO_3	2.00	4.74×10^{3}	1.00×10^{-1}	0
CH_3COOH	4.10×10^{3}	6.20×10^{3}	2.00×10^{-2}	4079
CH_3CHO	5.91	5.89×10^{3}	3.00×10^{-2}	0
PAN	2.80	5.73×10^{3}	1.00×10^{-1}	0
CH_3CH_2OH	2.00×10^{2}	6.63×10^{3}	9.00×10^{-3}	0
ETHGLY	4.00×10^{6}	5.00×10^{3}	1.00×10^{-1}	0
CH_3CO_3	1.00×10^{-1}	5.00×10^{3}	1.00×10^{-1}	0
$HOCH_2CHO$	2.40×10^{3}	3.85×10^{3}	1.00×10^{-1}	0
GLYOX	1.19×10^{3}	7.48×10^{3}	1.00×10^{-1}	0
CH_3CO_3H	8.40×10^{2}	5.30×10^{3}	1.00×10^{-1}	0
$HOCH_2CO_3H$	4.80×10^{4}	6.01×10^{3}	1.00×10^{-1}	0
C_2H_5OOH	3.34×10^{2}	6.00×10^{3}	1.00×10^{-1}	0
HOOCCOOH	5.00×10^{8}	5.00×10^{3}	1.00×10^{-1}	0
$HOOCH_2CO_2H$	1.50×10^{6}	6.01×10^{3}	1.00×10^{-1}	0
$HOCH_2CO_2H$	2.40×10^{4}	4.03×10^{3}	1.00×10^{-1}	0
$CH_3CHOHOH$	7.63×10^{3}	5.00×10^{3}	1.00×10^{-1}	0
CHOHOHCHOHOH	5.71×10^{6}	5.00×10^{3}	1.00×10^{-1}	0

CHOCHOHOH	2.58×10^{3}	5.00×10^{3}	1.00×10^{-1}	0
$HOOCH_2CHOHOH$	2.09×10^{5}	5.00×10^{3}	1.00×10^{-1}	0
$CH3COCH_3$	2.78×10^{1}	5.53×10^{3}	3.72×10^{-3}	6395
MACR	4.90	4.30×10^{3}	1.00×10^{-1}	0
MVK	2.60	4.80×10^{3}	1.00×10^{-1}	0
C_6H_5O	2.90×10^{3}	6.80×10^{3}	1.00×10^{-1}	0
C_6H_5OOH	2.90×10^{3}	6.80×10^{3}	1.00×10^{-1}	0
DNPHEN	2.30×10^{3}	5.00×10^{3}	1.00×10^{-1}	0
DNPHENOOH	2.30×10^{3}	5.00×10^{3}	1.00×10^{-1}	0
PHENOL	2.90×10^{3}	6.80×10^{3}	1.00×10^{-1}	0
PHENOOH	2.00×10^{6}	5.00×10^{3}	1.00×10^{-1}	0
$HOC_6H_4NO_2$	1.40×10^{2}	5.70×10^{3}	1.00×10^{-1}	0
$C_6H_5CO_3H$	2.40×10^{4}	5.00×10^{3}	1.00×10^{-1}	0
CRESOL	2.90×10^{3}	6.80×10^{3}	1.00×10^{-1}	0
CRESOOH	2.00×10^{6}	5.00×10^{3}	1.00×10^{-1}	0
DNCRES	2.30×10^{3}	5.00×10^{3}	1.00×10^{-1}	0
DNCRESOOH	2.30×10^{3}	5.00×10^{3}	1.00×10^{-1}	0
OXYL1OOH	2.90×10^{3}	6.80×10^{3}	1.00×10^{-1}	0
TOL1O	2.90×10^{3}	6.80×10^{3}	1.00×10^{-1}	0
CL_2	9.20×10^{-2}	2.00×10^{3}	3.80×10^{-2}	6546
HOCL	6.60×10^{2}	5.88×10^{3}	5.00×10^{-1}	0
$CLNO_2$	4.50×10^{-2}	2.92×10^{3}	9.50×10^{-3}	0
BR_2	7.25×10^{-1}	4.39×10^{3}	3.80×10^{-2}	6546
HOBR	1.30×10^{3}	5.86×10^{3}	5.00×10^{-1}	0
BRCL	9.70×10^{-1}	5.60×10^{3}	3.80×10^{-2}	6546
SO_2	1.30	2.90×10^{3}	3.00×10^{-4}	0
NCL_3	9.90×10^{-2}	4.10×10^{3}	1.00×10^{-1}	0
$NHCL_2$	2.90×10^{1}	4.20×10^{3}	1.00×10^{-1}	0
NH_2CL	8.60×10^{1}	6.00×10^{3}	1.00×10^{-1}	0

Note:

(1) H^{cp} at T^{Θ} denotes the Henry's law constant at standard conditions. $d\ln H^{cp}/d(1/T)$ describes the temperature dependence of the Henry's law constants. A complete definition and calculation of Henry's law constants shown in this table is referred to Sander (*40*), where the compiled data in the first row with the highest reliability is selected.

(2) α^{Θ} describes the mass accommodation coefficient at standard conditions. $d\ln[\alpha/(1-\alpha)]/d(1/T)$ represents the temperature dependence of the mass accommodation coefficients. These data are referred to Soni et al. (*49*) except for NO_2 (*93*), CO_2 *(94)*, and SO_2 (*94*). A value of 0.1 is assumed if the accommodation coefficient is unknown.

(3) Name of the species shown in this table is written in the MCM style. The numerical format of H^{cp} and α^{Θ} follows scientific notation.

Table S3 Mixing ratios and limit of detection (LOD) for the reported species (unit: ppt).

Species	Winter Beijing			Summer Beijing			New Delhi		
	Median	Average	LOD	Median	Average	LOD	Median	Average	LOD
NCl_3	0.4	1.0	0.1	0.5	1.3	0.1	3.9	5.9	0.3
$NHCl_2$	0.6	0.9	0.2	-	-	7.4	42.4	77.3	22.6
Cl_2	1.3	2.8	0.2	1.2	2.5	0.1	6.1	8.6	0.6
$ClNO_2$	28.7	90.5	0.2	20.2	110.1	0.9	31.9	113.5	1.6
N_2O_5	31.2	105.1	3.2	8.9	41.4	4.5	2.7	15.9	0.6

Note: LOD was estimated as three times the standard deviation of the hourly averaged background signals. Species sensitivities at H_2O mixing ratio of 0.24% (average RH 44%, T -1.0 ^{o}C) and 1.34% (average RH 67%, T 24.0 ^{o}C) was applied in winter and summer Beijing, respectively. In New Delhi, LOD was calculated at a H_2O mixing ratio of 1.80% (average RH 61%, T 24 ^{o}C during the winter campaign).

Table S4 Summary of the supporting measurements.

Target species		Site	Measured period	Instrumentation	Raw time resolution
Particle number size distribution	1 nm~10 μm	BUCT	2022.1.22~2.28; 2022.6.1~8.17	DEG-SMPS, 1 -7.5 nm, custom-made	5 min
				PSD system, 3 nm-10 μm, custom-made	
VOCs and monochloramine	C_2H_2, C_2H_6, C_3H_8, *i*-C_4H_{10}, *n*-C_4H_{10}, *i*-C_5H_{12}	THU	2022.1.22~2.28; 2022.7.1~7.31	GC-MS (Agilent Tech,7890/5975)	12 h
	C_3H_6, C_5H_8, C_5H_{10}, C_6H_6, C_6H_{12}, C_7H_8, C_8H_8, C_8H_{10}, C_9H_{12}, $C_{10}H_{16}$, NH_2Cl		2022.1.22~2.28 2022.6.6~8.17	Vocus PTR (Tofwerk AG)	1 s
O_3		THU & BUCT	2022.1.22~2.28; 2022.6.1~8.17	Model 49i, UV Photometric O_3 Analyzer, Thermo Scientific	1 min
NO, NO_2				Model 42i-TL, Chemiluminescence NO-NO_2-NO_x Analyzer, Thermo Scientific	1 min
CO		BUCT		Model 48i, Gas Filter Correlation CO Analyzer, Thermo Scientific	1 min
SO_2				Model 43i, Pulsed Fluorescence SO_2 Analyzer, Thermo Scientific	1 min
Inorganic gases (HCl, HONO, HNO_3, NH_3, SO_2) and water-soluble ions in $PM_{2.5}$ (SO_4^{2-}, NO_3^-, NH_4^+, Cl^-, K^+, Na^+, Ca^{2+}, Mg^{2+})		THU & BUCT		Monitor for AeRosols and Gases (MARGA, Metrohm)	1 h
j_{NO2}		BUCT		Filter radiometer (Metcon)	1 min

Table S5 The gas-phase chloramine chemistry module used in this study.

No.	Reaction	Rate constant	Unit	Reference
1	$NCl_3 + hv \rightarrow NCl_2 + Cl\cdot$	$5.79\times10^{-1}\times j_{NO2}$	s^{-1}	This study
2	$NCl_3 + Cl\cdot \rightarrow NCl_2\cdot + Cl_2$	1.60×10^{-12}	$cm^3\ s^{-1}\ molecule^{-1}$	*(75, 95)*
3	$NCl_2\cdot + NCl_2\cdot \rightarrow N_2 + Cl_2 + 2Cl\cdot$	6.00×10^{-13}	$cm^3\ s^{-1}\ molecule^{-1}$	*(95)*
4	$NCl_3 + NCl_2\cdot \rightarrow N_2 + Cl_2 + 3Cl\cdot$	$3.40\times10^{-11}\times\exp(-3250/T)$	$cm^3\ s^{-1}\ molecule^{-1}$	*(96)*
5	$NHCl_2 + hv \rightarrow Cl\cdot + NHCl\cdot$	$8.18\times10^{-2}\times j_{NO2}$	s^{-1}	This study

Note: The rate constants for bimolecular reactions (2-4) are currently not available under ambient conditions and are inferred from previous studies conducted under low-pressure conditions. Here we do not expect a remarkable pressure dependence on the rate constants. Additional gas-phase reactions concerning chloramines or $NCl_2\cdot$ could exist in the atmosphere, which requires further studies to reveal.

Table S6 Four simulation scenarios designed in the box model.

Scenario	Purpose	Default MCM	Gas-phase chlorine chemistry	Multiphase chloramine chemistry	Constrain Cl_2 and $ClNO_2$	Constrain chloramine	Note
1	Evaluate the impacts of observed chloramines on P(Cl·)	Yes	Yes	Yes	Yes	Yes	Beijing, New Delhi, and Toronto
2	Simulate the time series of chloramines and investigate their production and loss	Yes	Yes	Yes	Yes	No	Beijing
3	Evaluate the impacts of the proposed mechanism	Yes	Yes	Yes	No	No	Beijing
		Yes	Yes	No	No	No	Beijing
4	Simulate the average diurnal chloramine levels	Yes	Yes	Yes	Yes	No	Hong Kong, Nanjing, Cape Verde, and an Arctic site

Note: (1) Default MCM indicates all MCM reactions considered. Reference: Jenkin et al (*45*) shown in the main text.
(2) Gas-phase chlorine chemistry is referred to Xia et al. (2022) (*46*) shown in the main text.
(3) Multiphase chloramine chemistry is developed in this study. Refer to Table S7 in the SI.
(4) Combining scenarios 1 and 2, we simulated chloramine contributions to P(Cl·) at Hong Kong, Nanjing, Cape Verde, and an Arctic site where relevant supporting data, e.g., the observed diurnal level of Cl_2 and $ClNO_2$, meteorological parameters, etc., was available.

Table S7 Compilation of chloramine-related aqueous-phase reactions.

No.	Reaction	Rate constant	Unit	Ref.
Aqueous-phase reactions				
1) NH_2Cl production and loss: CA is the abbreviation for chloramine here				
CA1	NH3_aq + HOCL_aq = NH2CL_aq + H2O_aq	$5.40\times10^{9}\times\exp(-2237/T)$	$M^{-1}\ s^{-1}$	(*97*)
CA2	NH3_aq + CL2_aq = NH2CL_aq + Hp_aq + CLm_aq	4.00×10^{9}	$M^{-1}\ s^{-1}$	(*27*)
CA3	NH4p_aq + CLOm_aq = NH2CL_aq + H2O_aq	5.93×10^{4}	$M^{-1}\ s^{-1}$	(*97*)
CA4	NH3CLp_aq = NH2CL_aq + Hp_aq	$1.00\times10^{6}/28$*	s^{-1}	(*25*)
CA5	NH2CL_aq + (H2O_aq) = HOCL_aq + NH3_aq	2.11×10^{-5}	s^{-1}	(*98*)
CA6	NH2CL_aq + SO32m_aq + (NH4p_aq) = NH3_aq + NH3_aq + CLSO3m_aq	$1.70\times10^{2}\times$NH4p†	$M^{-1}\ s^{-1}$	(*81*)
CA7	NH2CL_aq + SO32m_aq + (H2O_aq) = OHm_aq + NH3_aq + CLSO3m_aq	7.70×Hp	$M^{-1}\ s^{-1}$	(*81*)
CA8	NH2CL_aq + NO2m_aq + (Hp_aq) = NH3_aq + CLNO2_aq	$7.60\times10^{6}\times$Hp	$M^{-1}\ s^{-1}$	(*99*)
CA9	NH2CL_aq + Hp_aq = NH3CLp_aq	1.00×10^{6}	s^{-1}	(*25*)
CA10	NH2CL_aq + CL_aq = NHCL_aq + CLm_aq + Hp_aq	1.00×10^{9}	$M^{-1}\ s^{-1}$	(*100*)
CA11	NH2CL_aq + OH_aq = NHCL_aq + H2O_aq	1.02×10^{9}	$M^{-1}\ s^{-1}$	(*100*)
CA12	NH2CL_aq + CL2m_aq = NHCL_aq + 2CLm_aq + Hp_aq	1.14×10^{7}	$M^{-1}\ s^{-1}$	(*101*, *102*)
2) $NHCl_2$ production and loss：				
CA13	NH2CL_aq + HOCL_aq = NHCL2_aq + H2O_aq	$3.00\times10^{5}\times\exp(-2010/T)$	$M^{-1}\ s^{-1}$	(*103*)
CA14	NH2CL_aq + NH2CL_aq + (Hp_aq) → NHCL2_aq + NH3_aq	$1.05\times10^{7}\times\exp(-2169/T)\times$Hp	$M^{-1}\ s^{-1}$	(*104*)
CA15	NH2CL_aq + NH2CL_aq + (H2CO3_aq) → NHCL2_aq + NH3_aq	$8.19\times10^{6}\times\exp(-2169/T)\times$H2CO3	$M^{-1}\ s^{-1}$	(*104*)
CA16	NH2CL_aq + NH2CL_aq + (HCO3m_aq) → NHCL2_aq + NH3_aq	$4.17\times10^{31}\times\exp(-22144/T)\times$HCO3	$M^{-1}\ s^{-1}$	(*104*)
CA17	NH2CL_aq + NH3CLp_aq = NHCL2_aq + NH4p_aq	3.35×10^{2}	$M^{-1}\ s^{-1}$	(*105*)

CA18	NHCL2_aq + CL_aq = NCL2_aq + CLm_aq + Hp_aq	1.00×10^{9}	$M^{-1}\ s^{-1}$	(*106*)
CA19	NHCL2_aq + OH_aq = NCL2_aq + H2O_aq	6.21×10^{8}	$M^{-1}\ s^{-1}$	(*106*)
CA20	NHCL2_aq + CL2m_aq = NCL2_aq + 2CLm_aq + Hp_aq	4.40×10^{6}	$M^{-1}\ s^{-1}$	(*101*)
CA21	NHCL2_aq + (H2O_aq) = HOCL_aq + NH2CL_aq	6.39×10^{-7}	s^{-1}	(*38*)
CA22	NHCL2_aq + OH_aq = products	1.11×10^{2}	$M^{-1}\ s^{-1}$	(*107*)
CA23	NHCL2_aq + SO32m_aq + (H2O_aq) = NH2CL_aq + CLSO3m_aq + OHm_aq	5.80×10^{6}	$M^{-1}\ s^{-1}$	(*39*)
CA24	NHCL2_aq + NH4p_aq = 2NH2CL_aq + Hp_aq	6.40×10^{-3}	$M^{-1}\ s^{-1}$	(*27*)
CA25	NHCL2_aq + NH3_aq = NH2CL_aq + NH2CL_aq	$6.00\times10^{4}\times$Hp	$M^{-1}\ s^{-1}$	(*38*)
CA26	NHCL2_aq + OHm_aq = NH2CL_aq + CLm_aq	1.50×10^{2}	$M^{-1}\ s^{-1}$	(*107*)
CA27	NHCL2_aq + NH2CL_aq = products	1.53×10^{-2}	$M^{-1}\ s^{-1}$	(*38*)
3) NCl_3 production and loss ：				
CA28	NHCL2_aq + HOCL_aq + (OHm_aq) = NCL3_aq + OHm_aq + H2O_aq	3.3×10^{9}	$M^{-1}\ s^{-1}$	(*108*)
CA29	NCL3_aq + OHm_aq + (H2O_aq) = NHCL2_aq + HOCL_aq + OHm_aq	$1.40\times10^{-1}\times$OHm	$M^{-1}\ s^{-1}$	(*108*)
CA30	NCL3_aq + OHm_aq + (HCO3m_aq) = NHCL2_aq + HOCL_aq + CO3m2_aq	$6.50\times10\times$HCO3m	$M^{-1}\ s^{-1}$	(*108*)
CA31	NCL3_aq + SO32m_aq + (H2O_aq) = NHCL2_aq + CLSO3m_aq + OHm_aq	4.50×10^{9}	$M^{-1}\ s^{-1}$	(*39*)
CA32	NCL3_aq + HSO3m_aq = NHCL2_aq + CLSO3m_aq	1.40×10^{7}	$M^{-1}\ s^{-1}$	(*39*)
CA33	NHCL2_aq + NCL3_aq + (2H2O_aq) = 2HOCL_aq + products	5.56×10^{10}	$M^{-1}\ s^{-1}$	(*38*)
CA34	NH2CL_aq + NCL3_aq + (H2O_aq) = HOCL_aq + products	1.39×10^{9}	$M^{-1}\ s^{-1}$	(*38*)
4) Other related reactions:				
CA35	NHCL_aq + O2_aq = NHCLO2_aq	1.20×10^{8}	$M^{-1}\ s^{-1}$	(*101*, *102*)
CA36	NHCLO2_aq = NO_aq + product	1.00×10^{8}	s^{-1}	(*106*)
CA37	NHCLO2_aq = N2O_aq	6.70×10^{8}	s^{-1}	(*106*)
CA38	(Hp_aq) + CLOm_aq + NO2m_aq = NH3_aq + CLNO2_aq	$1.40\times10^{12}\times$Hp	$M^{-1}\ s^{-1}$	(*99*)

Note for the expression of reactions: aq represents aqueous, m and p stand for negative and positive ions, respectively, e.g., OHm_aq stands for OH^- while OH_aq stands for OH·. The specie in brackets indicate its concentration is constrained in the model.

*: K_{eq} was reported to be 28 M^{-1} (*25*), and we assume the reverse reaction rate constant is 1×10^{6} M^{-1} s^{-1}. M stands for mol L^{-1}.

†: NH4p stands for the NH_4^+ mole concentration (unit: M) while 1.70×10^{2} denotes the three-order rate constant in the unit of M^{-2} s^{-1}, which also applies for the remaining expression of rate constants in Table S7.

Table S8 Model input for simulations in Nanjing, Cape Verde, and an Arctic site.

Arctic site (Utqiaġvik, AK)				Cape Verde				Nanjing			
No.	Parameter	Average	Ref.	No.	Parameter	Value	Ref.	No.	Parameter	Average	Ref.
1	Latitude	71.275 °N	location	1	Latitude	17 °N	location	1	Latitude	32 °N	location
2	Longitude	156.641 ° W	location	2	Longitude	24 °W	location	2	Longitude	119 °E	location
3	Altitude (m)	10.0	location	3	Altitude (m)	10.0	location	3	Altitude (m)	10.0	location
4	Temp. (K)	268	(*109*)	4	Temp. (K)	272.2	(*57*)	4	Temp. (K)	290.8	(*22*)
5	RH (%)	78	(*110*)	5	RH (%)	80		5	RH (%)	67.4	
6	j_{NO2} (s^{-1})	3.5×10^{-3}	(*55*)	6	jNO_2 (s^{-1})	2.5×10^{-3}	(*111*)	6	jNO_2 (s^{-1})	1.8×10^{-3}	
7	Sa (um^2 cm^{-3})	10.4	(*109*)	7	Sa (um^2 cm^{-3})	200	assumed	7	Sa (um^2 cm^{-3})	689	
8	BLH (m)	400	(*112*)	8	BLH (m)	1000	(*57*)	8	BLH (m)	1000	(*113*)
9	MLH (m)	10	(*111*)	9	MLH (m)	50	(*111*)	9	MLH (m)	50	(*111*)
10	H_2 (ppb)	550	default	10	H_2 (ppb)	550	default	10	H_2 (ppb)	550	default
11	NH_3 (ppb)	0.26	(*114*)	11	NH_3 (ppb)	0.651	(*115*)	11	NH_3 (ppb)	7.79	(*22*)
12	NO (ppb)	1.68	(*55*)	12	NO (ppb)	2.29	(*111*)	12	NO (ppb)	3.65	
13	NO_2 (ppb)		modelled	13	NO_2 (ppb)	6.28		13	NO_2 (ppb)	13.21	
14	O_3 (ppb)	29.8	(*55*)	14	O_3 (ppb)	30.86	(*57*)	14	O_3 (ppb)	26.1	
15	CO (ppb)	127	(*109*)	15	CO (ppb)	96		15	CO (ppb)	445	
16	HONO (ppb)		modelled	16	HONO (ppb)	0.01	(*111*)	16	HONO (ppb)	0.626	
17	$ClNO_2$ (ppb)	9.31×10^{-4}	(*55*)	17	$ClNO_2$ (ppb)	N/A	modelled	17	$ClNO_2$ (ppb)	0.539	
18	Cl_2 (ppt)	2.12		18	Cl_2 (ppt)	4.96	(*57*)	18	Cl_2 (ppt)	22	
19	CH_4 (ppb）	1923	(*109*)	19	CH_4 (ppb）	1800		19	CH_4 (ppb）	1900	(*112*)
20	C_2H_6 (ppb）	1.24		20	C_2H_6 (ppb）	4.88×10^{-1}	(*111*)	20	C_2H_6 (ppb）	1.60	
21	C_3H_8 (ppb)	1.77×10^{-1}		21	C_3H_8 (ppb)	2.89×10^{-2}		21	C_3H_8 (ppb)	1.49	
22	i-C_4H_{10} (ppb)	1.40×10^{-2}		22	i-C_4H_{10} (ppb)	3.26×10^{-3}		22	i-C_4H_{10} (ppb)	4.80×10^{-1}	
23	n-C_4H_{10} (ppb)	2.50×10^{-2}		23	n-C_4H_{10} (ppb)	2.21×10^{-2}		23	n-C_4H_{10} (ppb)	7.80×10^{-1}	
24	C_6H_6 (ppb)	2.70×10^{-2}	(*110*)	24	i-C_5H_{12} (ppb)	1.95×10^{-3}		24	i-C_5H_{12} (ppb)	4.60×10^{-1}	

25	HCHO (ppb)	2.20×10^{-1}		25	n-C_5H_{12} (ppb)	5.16×10^{-3}		25	n-C_5H_{12} (ppb)	4.60×10^{-1}	
26	LWC (m^3/m^3)	2.60×10^{-12}	(*109*)	26	n-C_6H_{14} (ppb)	1.79×10^{-2}		26	n-C_6H_{14} (ppb)	2.80×10^{-1}	
27	OH^- (mol L^{-1})	3.80×10^{-11}	(*116*) & Isorropia II	27	C_2H_4 (ppb)	2.31×10^{-2}		27	C_2H_4 (ppb)	4.40×10^{-1}	
28	H^+ (mol L^{-1})	2.60×10^{-4}		28	C_3H_6 (ppb)	7.80×10^{-3}		28	C_3H_6 (ppb)	2.80×10^{-1}	
29	NH_4^+ (mol L^{-1})	1.84	(*116*)	29	C_5H_8 (ppb)	7.28×10^{-2}		29	C_5H_8 (ppb)	2.00×10^{-1}	
30	Cl^- (mol L^{-1})	2.13	(*109*) & Isorropia II	30	C_6H_6 (ppb)	1.41×10^{-2}		30	C_6H_6 (ppb)	7.40×10^{-1}	
31	NO_3^- (mol L^{-1})	2.38	(*116*) & Isorropia II	31	C_7H_8 (ppb)	2.17×10^{-3}		31	C_7H_8 (ppb)	1.14	
32	SO_4^{2-} (mol L^{-1})	0.48		32	C_8H_{10} (ppb)	7.39×10^{-2}		32	m-C_8H_{10} (ppb)	3.00×10^{-1}	
33	H_2O (mol L^{-1})	35.5		33	$C_{10}H_{16}$ (ppb)	1.08×10^{-3}		33	o-C_8H_{10} (ppb)	2.00×10^{-1}	
				34	LWC (m^3/m^3)	1.20×10^{-10}		34	HCHO (ppb)	1.40	
				35	OH^- (mol L^{-1})	5.60×10^{-11}		35	CH_3CHO (ppb)	3.69	
				36	H^+ (mol L^{-1})	1.78×10^{-4}	(*115*) & Isorropia II	36	LWC (m^3/m^3)	1.60×10^{-10}	
				37	NH_4^+ (mol L^{-1})	6.38×10^{-2}		37	OH^- (mol L^{-1})	2.50×10^{-11}	
				38	Cl^- (mol L^{-1})	1.59		38	H^+ (mol L^{-1})	4.00×10^{-4}	(*22*) & Isorropia II
				39	NO_3^- (mol L^{-1})	4.23×10^{-1}		39	NH_4^+ (mol L^{-1})	6.73	
				40	SO_4^{2-} (mol L^{-1})	1.50		40	Cl^- (mol L^{-1})	0.13	
				41	H_2O (mol L^{-1})	36.80		41	NO_3^- (mol L^{-1})	3.52	
								42	SO_4^{2-} (mol L^{-1})	1.6	
								43	H_2O (mol L^{-1})	27.8	

Note: The campaign average values are shown here. The input for Hong Kong was all retrieved from Table S3 in Xia et al. (*46*) and was not listed above.

Table S9 Improved XGBoost performance after feature selection compared with all features included.

	Internal model validation				Test dataset			
	R^2	Corr	RMSE	MAE	R^2	Corr	RMSE	MAE
With feature selection	0.796	0.895	0.595	0.288	0.829	0.912	0.548	0.366
W/o feature selection	0.789	0.890	0.605	0.297	0.807	0.906	0.583	0.362

Note: The average performance metrics on the validation datasets in the 10-fold cross-validation process are presented as internal model validation, while the test dataset results are the metrics evaluating the model performance with the test dataset that was excluded from the training and validation datasets. W/o means "without".

Table S10 Features used in the XGBoost model.

No.	Classification	Abbreviation	Full Name	Unit	Selected?
1	Meteorology	T	Temperature	°C	Yes
2		RH	Relative humidity	%	Yes
3	Trace gas	NO	Nitric oxide	ppb	No
4		NO_2	Nitrogen dioxide	ppb	No
5		O_3	Ozone	ppb	No
6		SO_2	Sulfur dioxide	ppb	Yes
7		CO	Carbon monoxide	ppb	No
8		NH_3	Ammonia	μg m^{-3}	Yes
9		Cl_2	Molecular chlorine	ppt	Yes
10		$ClNO_2$	Nitryl chloride	ppt	Yes
11		N_2O_5	Dinitrogen pentoxide	ppt	Yes
12	Aerosol physicochemical properties	Aerosol Sa	Aerosol surface area density	$μm^2$ cm^{-3}	Yes
13		Aerosol pH	Aerosol pH	N/A	No
14		$PM_{2.5}$	$PM_{2.5}$ mass concentration	μg m^{-3}	No
15		Cl^-	Chloride mass concentration	μg m^{-3}	No
16		NH_4^+	Ammonium mass concentration	μg m^{-3}	No
17		SO_4^{2-}	Sulfate mass concentration	μg m^{-3}	Yes
18		NO_3^-	Nitrate mass concentration	μg m^{-3}	No
19		j_{NO2}	Photolysis frequency of NO_2	s^{-1}	Yes
20		Season index	1 for winter, 2 for summer	N/A	No

Table S11 First-stage ML model selection: 4 evaluation metrics over 36 common ML models.

ID	ML model	R^2	Corr	RMSE	MAE
1	ExtraTreesRegressor	0.784	0.890	0.610	0.262
2	LGBMRegressor	0.769	0.879	0.631	0.316
3	HistGradientBoostingRegressor	0.768	0.878	0.634	0.316
4	XGBRegressor	0.757	0.872	0.647	0.308
5	RandomForestRegressor	0.755	0.874	0.651	0.307
6	MLPRegressor	0.732	0.859	0.678	0.399
7	BaggingRegressor	0.727	0.858	0.688	0.328
8	KNeighborsRegressor	0.651	0.812	0.779	0.369
9	GradientBoostingRegressor	0.637	0.806	0.795	0.446
10	NuSVR	0.504	0.775	0.934	0.443
11	SVR	0.501	0.775	0.936	0.440
12	ExtraTreeRegressor	0.480	0.748	0.935	0.413
13	DecisionTreeRegressor	0.443	0.726	0.976	0.420
14	BayesianRidge	0.269	0.528	1.129	0.714
15	RidgeCV	0.269	0.528	1.129	0.713
16	Ridge	0.268	0.528	1.129	0.713
17	TransformedTargetRegressor	0.268	0.528	1.129	0.713
18	LinearRegression	0.268	0.528	1.129	0.713
19	LassoLarsCV	0.267	0.526	1.131	0.710
20	LassoCV	0.267	0.526	1.131	0.710
21	ElasticNetCV	0.267	0.526	1.131	0.709
22	LassoLarsIC	0.267	0.527	1.130	0.714
23	SGDRegressor	0.265	0.527	1.131	0.722
24	OrthogonalMatchingPursuitCV	0.228	0.488	1.161	0.731
25	PoissonRegressor	0.217	0.513	1.171	0.726
26	TweedieRegressor	0.210	0.499	1.176	0.727

27	GammaRegressor	0.204	0.493	1.181	0.675
28	HuberRegressor	0.152	0.507	1.220	0.619
29	OrthogonalMatchingPursuit	0.139	0.383	1.227	0.771
30	LinearSVR	0.116	0.503	1.246	0.617
31	AdaBoostRegressor	-0.059	0.716	1.339	1.198
32	KernelRidge	-0.293	0.528	1.495	1.060
33	LarsCV	-0.524	0.390	1.546	1.038
34	PassiveAggressiveRegressor	-0.838	0.322	1.740	1.274
35	RANSACRegressor	-0.962	0.164	1.825	1.145
36	Lars	-4.154	0.307	2.455	1.664

Note: The model performance on the validation datasets in the 10-fold cross-validation process is presented. Coefficient of determination (R^2), root-mean-square error (RMSE), mean square error (MSE), and mean absolute error (MAE) are computed.

Table S12 Summary of second-stage hyperparameter optimization results. Model performance was evaluated on validation datasets using 10-fold cross-validation. The following regression metrics were computed: coefficient of determination (R^2), root mean squared error (RMSE), mean squared error (MSE), and mean absolute error (MAE).

ID	ML model	Hyperparameters	R^2	Corr	RMSE	MAE
1	XGB	the maximum depth of the tree<br>learning rate<br>the number of trees<br>the minimum weight in a leaf,<br>subsample ratio of the training instance<br>the number of features when creating a tree<br>L1 regularization parameter<br>L2 regularization parameter	0.789	0.890	0.605	0.297
2	ExtraTrees	the number of trees<br>the maximum depth of the tree<br>the minimum amount of data when splitting nodes<br>the minimum amount of data in a leaf	0.787	0.891	0.607	0.266

		the number of features when splitting leaves<br>impurity decrease threshold<br>bootstrap method				
3	LGBM	type of boosting<br>the maximum number of leaves per estimator<br>the maximum depth of the estimator<br>learning rate<br>the number of trees<br>the minimum amount of data in a leaf<br>subsample ratio of the training instance<br>L1 regularization parameter<br>L2 regularization parameter	0.778	0.883	0.620	0.299
4	HGBoosting	learning rate<br>the maximum number of trees<br>the maximum number of leaves per tree<br>the maximum depth of the tree<br>the minimum number of samples per leaf<br>L2 regularization parameter<br>maximum number of bins	0.776	0.883	0.619	0.288
5	RF	the number of trees<br>the maximum depth of the tree<br>the minimum number of data when splitting nodes<br>the minimum amount of data in a leaf<br>the number of features when splitting leaves<br>impurity decrease threshold<br>bootstrap method	0.755	0.874	0.651	0.307

Table S13 Input parameters used in the box model. Campaign-averaged values are shown here. An exception is that the daytime maximum value of $j(NO_2)$ is shown instead of the average value.

No.	Species/parameters	Winter	Summer	Source
1	Latitude (degree)	39.9	39.9	location
2	Longitude (degree)	116.3	116.3	location
3	Altitude (m)	15.0	15.0	location
4	Temperature (K)	272.2	299.6	Meteorological station
5	RH (%)	43.4	71.7	Meteorological station
6	$j(NO_2)$ (s^{-1})	4.0×10^{-3}	4.4×10^{-3}	Filter radiometer
7	S_a (μm^2 cm^{-3})[1]	566.4	547.5	PNSD[2]
8	BLH (m)[3]	301	589	Lidar
9	MLH (m)[4]	50	50	Zhang et al. (*117*)
10	H_2 (ppb)	550	550	Model default setting
11	NH_3 (ppb)	5.4	18.2	MARGA[5]
12	NO (ppb)	3.3	0.6	Thermo 42i-TL
13	NO_2 (ppb)	12.8	5.6	Thermo 42i-TL
14	O_3 (ppb)	24.6	49.9	Thermo 49i
15	CO (ppb)	987.6	423	Thermo 48i
16	SO_2 (ppb)	1.1	0.5	Thermo 43i
17	HONO (ppb)	0.7	1.1	MARGA
18	N_2O_5 (ppb)	0.1	0.03	Iodide-LTof-CIMS[6]
19	$ClNO_2$ (ppb)	0.1	0.1	Iodide-LTof-CIMS
20	Cl_2 (ppt)	2.7	2.5	Iodide-LTof-CIMS
21	NCl_3 (ppt)	1.0	1.2	Iodide-LTof-CIMS
22	$NHCl_2$ (ppt)	0.9	2.1	Iodide-LTof-CIMS
23	CH_4 (ppb)	2000	2000	Assumed
24	C_2H_6 (ppb)	3.7	2.5	Canister sampling & GC[7]
25	C_3H_8 (ppb)	2.7	2.7	Canister sampling & GC
26	i-C_4H_{10} (ppb)	0.7	0.9	Canister sampling & GC
27	n-C_4H_{10} (ppb)	0.6	1.2	Canister sampling & GC
28	i-C_5H_{12} (ppb)	1.1	1.2	Canister sampling & GC
29	n-C_5H_{12} (ppb)	1.1	0.5	Canister sampling & GC
30	n-C_6H_{14} (ppb)	0.2	0.4	Canister sampling & GC
31	C_2H_2 (ppb)	1.8	1.4	Canister sampling & GC
32	C_3H_6 (ppb)	0.2	0.3	Vocus-PTR[8]
33	C_5H_8 (ppb)	0.4	0.6	Vocus-PTR
34	C_6H_6 (ppb)	1.2	0.3	Vocus-PTR
35	C_7H_8 (ppb)	0.9	0.5	Vocus-PTR
36	C_8H_{10} (ppb)	0.5	0.2	Vocus-PTR
37	C_9H_{12} (ppb)	0.1	0.1	Vocus-PTR

38	$C_{10}H_{16}$ (ppb)	0.04	0.06	Vocus-PTR
39	LWC (m^3/m^3)[9]	1.8×10^{-11}	3.7×10^{-11}	MARGA & Isorropia II
40	OH^- (mol L^{-1})	1.8×10^{-10}	1.1×10^{-11}	MARGA & Isorropia II
41	H^+ (mol L^{-1})	5.6×10^{-5}	8.9×10^{-4}	MARGA & Isorropia II
42	HCO_3^- (mol L^{-1})	7.1×10^{-6}	2.8×10^{-5}	Model-simulated
43	H_2CO_3 (mol L^{-1})	$1.2E\times10^{-3}$	3.4×10^{-3}	Model-simulated
44	NH_4^+ (mol L^{-1})	8.7	5.8	MARGA & Isorropia II
45	Cl^- (mol L^{-1})	1.4	0.5	MARGA & Isorropia II
46	NO_3^- (mol L^{-1})	6.2	3.9	MARGA & Isorropia II
47	SO_4^{2-} (mol L^{-1})	1.1	1.4	MARGA & Isorropia II
48	H_2O (mol L^{-1})	31.0	37.7	MARGA & Isorropia II
49	HOCl_aq (mol L^{-1})	1.1×10^{-10}	2.4×10^{-7}	Model-simulated
50	Cl_2_aq (mol L^{-1})	2.2×10^{-5}	5.4×10^{-6}	Model-simulated

Notes for the abbreviations**:** 1. S_a: aerosol surface area density. 2. PNSD: particle number size distributions. 3. BLH: boundary layer height. 4. MLH: mixing layer height. 5. MARGA: Online analyzer of Monitoring of Aerosols and gases 6. Iodide-LTof-CIMS: iodide-adduct long-time-of-flight chemical ionization mass spectrometer 7. GC: gas chromatography 8. Vocus-PTR: Vocus proton-transfer-reaction mass spectrometer 9. LWC: liquid water content.

Table S14 Sensitivity test of rate constants of key chloramine-related reactions. The units for the simulated chloramine mixing ratios are ppt.

No.	Tested reaction	Simulated NH_2Cl	Simulated $NHCl_2$	Simulated NCl_3
1	Original model simulations in summer Beijing	13.678	0.026	1.543
2	NH3_aq + CL2_aq = NH2CL_aq + Hp_aq + CLm_aq	13.678	0.026	1.543
3	NH3_aq + HOCL_aq = NH2CL_aq + H2O_aq	13.844	0.027	0.804
4	NHCL2_aq + NH3_aq = NH2CL_aq + NH2CL_aq	13.837	0.014	0.817
5	NH2CL_aq + NH2CL_aq + (Hp_aq) = NHCL2_aq + NH3_aq	13.678	0.026	1.543
6	NHCL2_aq + HOCL_aq + OHm_aq = NCL3_aq + OHm_aq + H2O_aq	13.393	0.024	2.864
7	NCL3 + hv = NCL2 + CL	13.679	0.026	1.427
8	NCL3 + CL = NCL2 + CL2	13.678	0.026	1.543
9	NCL2 + NCL2 = N2 + CL2 + 2CL	13.678	0.026	1.543
10	NCL3 + NCL2 = N2 + CL2 + 3CL	13.678	0.026	1.543
11	NHCL2 + hv = CL + NHCL	13.677	0.024	1.543

Note: (1) The absence of subscripts in the table is intentional, as the model itself does not include subscripts.

(2) Diurnal average of box-model simulated mixing ratio when the tested rate constant was multiplied by a factor of 2.
(3) Chemical species names are written in MCM style. The species marked with "_aq" means aqueous species, while those without the "_aq" mark represent gas-phase species.

Table S15 Phase transfer processes and aqueous-phase reactions incorporated in the model.

The full content of Table S15 is provided in the Excel file available at https://doi.org/10.6084/m9.figshare.30305371.

REFERENCES AND NOTES

1. M. J. Molina, F. S. Rowland, Stratospheric sink for chlorofluoromethanes: Chlorine atom-catalysed destruction of ozone. *Nature* **249**, 810–812 (1974).

2. X.-C. He, M. Simon, S. Iyer, H.-B. Xie, B. Rörup, J. Shen, H. Finkenzeller, D. Stolzenburg, R. Zhang, A. Baccarini, Y. J. Tham, M. Wang, S. Amanatidis, A. A. Piedehierro, A. Amorim, R. Baalbaki, Z. Brasseur, L. Caudillo, B. Chu, L. Dada, J. Duplissy, I. El Haddad, R. C. Flagan, M. Granzin, A. Hansel, M. Heinritzi, V. Hofbauer, T. Jokinen, D. Kemppainen, W. Kong, J. Krechmer, A. Kürten, H. Lamkaddam, B. Lopez, F. Ma, N. G. A. Mahfouz, V. Makhmutov, H. E. Manninen, G. Marie, R. Marten, D. Massabò, R. L. Mauldin, B. Mentler, A. Onnela, T. Petäjä, J. Pfeifer, M. Philippov, A. Ranjithkumar, M. P. Rissanen, S. Schobesberger, W. Scholz, B. Schulze, M. Surdu, R. C. Thakur, A. Tomé, A. C. Wagner, D. Wang, Y. Wang, S. K. Weber, A. Welti, P. M. Winkler, M. Zauner-Wieczorek, U. Baltensperger, J. Curtius, T. Kurtén, D. R. Worsnop, R. Volkamer, K. Lehtipalo, J. Kirkby, N. M. Donahue, M. Sipilä, M. Kulmala, Iodine oxoacids enhance nucleation of sulfuric acid particles in the atmosphere. *Science* **382**, 1308–1314 (2023).

3. A. Saiz-Lopez, R. P. Fernandez, Q. Li, C. A. Cuevas, X. Fu, D. E. Kinnison, S. Tilmes, A. S. Mahajan, J. C. Gómez Martín, F. Iglesias-Suarez, R. Hossaini, J. M. C. Plane, G. Myhre, J.-F. Lamarque, Natural short-lived halogens exert an indirect cooling effect on climate. *Nature* **618**, 967–973 (2023).

4. W. R. Simpson, S. S. Brown, A. Saiz-Lopez, J. A. Thornton, R. von Glasow, Tropospheric halogen chemistry: Sources, cycling, and impacts. *Chem. Rev.* **115**, 4035–4062 (2015).

5. R. Atkinson, J. Arey, Atmospheric degradation of volatile organic compounds. *Chem. Rev.* **103**, 4605–4638 (2003).

6. X. Chen, M. Xia, W. Wang, H. Yun, D. Yue, T. Wang, Fast near-surface $ClNO_2$ production and its impact on O_3 formation during a heavy pollution event in South China. *Sci. Total Environ.* **858**, 159998 (2023).

7. M. S. Choi, X. Qiu, J. Zhang, S. Wang, X. Li, Y. Sun, J. Chen, Q. Ying, Study of secondary organic aerosol formation from chlorine radical-initiated oxidation of volatile organic

compounds in a polluted atmosphere using a 3D chemical transport model. *Environ. Sci. Technol.* **54**, 13409–13418 (2020).

8. J. A. Thornton, J. P. Kercher, T. P. Riedel, N. L. Wagner, J. Cozic, J. S. Holloway, W. P. Dube, G. M. Wolfe, P. K. Quinn, A. M. Middlebrook, B. Alexander, S. S. Brown, A large atomic chlorine source inferred from mid-continental reactive nitrogen chemistry. *Nature* **464**, 271–274 (2010).

9. C. W. Spicer, E. G. Chapman, B. J. Finlayson-Pitts, R. A. Plastridge, J. M. Hubbe, J. D. Fast, C. M. Berkowitz, Unexpectedly high concentrations of molecular chlorine in coastal air. *Nature* **394**, 353–356 (1998).

10. X. Peng, W. Wang, M. Xia, H. Chen, A. R. Ravishankara, Q. Li, A. Saiz-Lopez, P. Liu, F. Zhang, C. Zhang, L. Xue, X. Wang, C. George, J. Wang, Y. Mu, J. Chen, T. Wang, An unexpected large continental source of reactive bromine and chlorine with significant impact on wintertime air quality. *Natl. Sci. Rev.* **8**, nwaa304 (2021).

11. A. A. Angelucci, L. R. Crilley, R. Richardson, T. S. E. Valkenburg, P. S. Monks, J. M. Roberts, R. Sommariva, T. C. VandenBoer, Elevated levels of chloramines and chlorine detected near an indoor sports complex. *Environ. Sci. Process. Impacts* **25**, 304–313 (2023).

12. C. Wang, J. Liggio, J. J. B. Wentzell, S. Jorga, A. Folkerson, J. P. D. Abbatt, Chloramines as an important photochemical source of chlorine atoms in the urban atmosphere. *Proc. Natl. Acad. Sci. U.S.A.* **120**, e2220889120 (2023).

13. H. Delalu, L. Peyrot, C. Duriche, F. Elomar, M. Elkhatib, Synthesis of enriched solutions of chloramine starting from hypochlorite at high chlorometric degree. *Chem. Eng. J.* **83**, 219–224 (2001).

14. D. L. McCurry, The chloramine dilemma. *Science* **386**, 851–852 (2024).

15. T. Wu, T. Foldes, L. T. Lee, D. N. Wagner, J. Jiang, A. Tasoglou, B. E. Boor, E. R. Blatchley III, Real-time measurements of gas-phase trichloramine (NCl_3) in an indoor aquatic center. *Environ. Sci. Technol.* **55**, 8097–8107 (2021).

16. M. Hery, J. M. Gerber, G. Hecht, I. Subra, C. Possoz, S. Aubert, M. Dieudonne, J. C. Andre, Exposure to chloramines in a green salad processing plant. *Ann. Occup. Hyg.* **42**, 437–451 (1998).

17. J. M. Mattila, P. S. J. Lakey, M. Shiraiwa, C. Wang, J. P. D. Abbatt, C. Arata, A. H. Goldstein, L. Ampollini, E. F. Katz, P. F. DeCarlo, S. Zhou, T. F. Kahan, F. J. Cardoso-Saldana, L. H. Ruiz, A. Abeleira, E. K. Boedicker, M. E. Vance, D. K. Farmer, Multiphase chemistry controls inorganic chlorinated and nitrogenated compounds in indoor air during bleach cleaning. *Environ. Sci. Technol.* **54**, 1730–1739 (2020).

18. J. P. S. Wong, N. Carslaw, R. Zhao, S. Zhou, J. P. D. Abbatt, Observations and impacts of bleach washing on indoor chlorine chemistry. *Indoor Air* **27**, 1082–1090 (2017).

19. T. P. Riedel, T. H. Bertram, T. A. Crisp, E. J. Williams, B. M. Lerner, A. Vlasenko, S. M. Li, J. Gilman, J. de Gouw, D. M. Bon, N. L. Wagner, S. S. Brown, J. A. Thornton, Nitryl chloride and molecular chlorine in the coastal marine boundary layer. *Environ. Sci. Technol.* **46**, 10463–10470 (2012).

20. J. L. Fairey, J. R. Laszakovits, H. T. Pham, T. D. Do, S. D. Hodges, K. McNeill, D. G. Wahman, Chloronitramide anion is a decomposition product of inorganic chloramines. *Science* **386**, 882–887 (2024).

21. H. D. Osthoff, J. M. Roberts, A. R. Ravishankara, E. J. Williams, B. M. Lerner, R. Sommariva, T. S. Bates, D. Coffman, P. K. Quinn, J. E. Dibb, H. Stark, J. B. Burkholder, R. K. Talukdar, J. Meagher, F. C. Fehsenfeld, S. S. Brown, High levels of nitryl chloride in the polluted subtropical marine boundary layer. *Nat. Geosci.* **1**, 324–328 (2008).

22. M. Xia, X. Peng, W. Wang, C. Yu, P. Sun, Y. Li, Y. Liu, Z. Xu, Z. Wang, Z. Xu, W. Nie, A. Ding, T. Wang, Significant production of $ClNO_2$ and possible source of Cl_2 from N_2O_5 uptake at a suburban site in eastern China. *Atmos. Chem. Phys.* **20**, 6147–6158 (2020).

23. C. Fountoukis, A. Nenes, ISORROPIA II: A computationally efficient thermodynamic equilibrium model for K^+-Ca^{2+}-Mg^{2+}-NH_4^+-Na^+-SO_4^{2-}-NO_3^--Cl^--H_2O aerosols. *Atmos. Chem. Phys.* **7**, 4639–4659 (2007).

24. D. Trogolo, J. S. Arey, Equilibria and speciation of chloramines, bromamines, and bromochloramines in water. *Environ. Sci. Technol.* **51**, 128–140 (2017).

25. E. T. J. Gray, D. W. Margerum, R. P. Huffman, "Chloramine equilibria and the kinetics of disproportionation in aqueous solution," in *Organometals and Organometalloids* (ACS Symposium Series, American Chemical Society, 1979), chap. 16, vol. 82, pp. 264-277.

26. M. Hery, G. Hecht, J. M. Gerber, J. C. Gendre, G. Hubert, J. Rebuffaud, Exposure to chloramines in the atmosphere of indoor swimming pools. *Ann. Occup. Hyg.* **39**, 427–439 (1995).

27. D. W. Margerum, E. T. J. Gray, R. P. Huffman, "Chlorination and the formation of *N*-chloro compounds in water treatment," in *Organometals and Organometalloids* (ACS Symposium Series, American Chemical Society, 1979), chap. 17, vol. 82, pp. 278-291.

28. D. Sakic, M. Hanzevacki, D. M. Smith, V. Vrcek, A computational study of the chlorination and hydroxylation of amines by hypochlorous acid. *Org. Biomol. Chem.* **13**, 11740–11752 (2015).

29. Y. Tao, J. G. Murphy, The sensitivity of $PM_{2.5}$ acidity to meteorological parameters and chemical composition changes: 10-year records from six Canadian monitoring sites. *Atmos. Chem. Phys.* **19**, 9309–9320 (2019).

30. R. Zhang, M. Gen, D. Huang, Y. Li, C. K. Chan, Enhanced sulfate production by nitrate photolysis in the presence of halide ions in atmospheric particles. *Environ. Sci. Technol.* **54**, 3831–3839 (2020).

31. G. Zheng, H. Su, S. Wang, M. O. Andreae, U. Pöschl, Y. Cheng, Multiphase buffer theory explains contrasts in atmospheric aerosol acidity. *Science* **369**, 1374–1377 (2020).

32. G. F. Nordberg, N.-G. Lundstrom, B. Forsberg, A. Hagenbjork-Gustafsson, B. J.-s. Lagerkvist, J. Nilsson, M. Svensson, A. Blomberg, L. Nilsson, A. Bernard, X. Dumont, H. Bertilsson, K. Eriksson, Lung function in volunteers before and after exposure to trichloramine in indoor pool environments and asthma in a cohort of pool workers. *BMJ Open* **2**, e000973 (2012).

33. W. Lee, P. Westerhoff, Formation of organic chloramines during water disinfection – Chlorination versus chloramination. *Water Res.* **43**, 2233–2239 (2009).

34. M. Li, M. Xia, C. Lin, Y. Jiang, W. Sun, Y. Wang, Y. Zhang, M. He, T. Wang, Mechanistic insights into chloroacetic acid production from atmospheric multiphase volatile organic compound–chlorine chemistry. *Atmos. Chem. Phys.* **25**, 3753–3764 (2025).

35. K. He, F. Yang, Y. Ma, Q. Zhang, X. Yao, C. K. Chan, S. Cadle, T. Chan, P. Mulawa, The characteristics of $PM_{2.5}$ in Beijing, China. *Atmos. Environ.* **35**, 4959–4970 (2001).

36. R. Cai, D. Yang, Y. Fu, X. Wang, X. Li, Y. Ma, J. Hao, J. Zheng, J. Jiang, Aerosol surface area concentration: A governing factor in new particle formation in Beijing. *Atmos. Chem. Phys.* **17**, 12327–12340 (2017).

37. F. D. Lopez-Hilfiker, C. Mohr, M. Ehn, F. Rubach, E. Kleist, J. Wildt, T. F. Mentel, A. Lutz, M. Hallquist, D. Worsnop, J. A. Thornton, A novel method for online analysis of gas and particle composition: Description and evaluation of a Filter Inlet for Gases and AEROsols (FIGAERO). *Atmos. Meas. Tech.* **7**, 983–1001 (2014).

38. C. T. Jafvert, R. L. Valentine, Reaction scheme for the chlorination of ammoniacal water. *Environ. Sci. Technol.* **26**, 577–586 (1992).

39. B. S. Yiin, D. W. Margerum, Nonmetal redox kinetics: Reactions of sulfite with dichloramines and trichloramine. *Inorg. Chem.* **29**, 1942–1948 (1990).

40. R. Sander, Compilation of Henry's law constants (version 5.0.0) for water as solvent. *Atmos. Chem. Phys.* **23**, 10901–12440 (2023).

41. Y. Liu, C. Yan, Z. Feng, F. Zheng, X. Fan, Y. Zhang, C. Li, Y. Zhou, Z. Lin, Y. Guo, Y. Zhang, L. Ma, W. Zhou, Z. Liu, L. Dada, K. Dällenbach, J. Kontkanen, R. Cai, T. Chan, B. Chu, W. Du, L. Yao, Y. Wang, J. Cai, J. Kangasluoma, T. Kokkonen, J. Kujansuu, A. Rusanen, C. Deng, Y. Fu, R. Yin, X. Li, Y. Lu, Y. Liu, C. Lian, D. Yang, W. Wang, M. Ge, Y. Wang, D. R. Worsnop, H. Junninen, H. He, V.-M. Kerminen, J. Zheng, L. Wang, J. Jiang, T. Petäjä, F. Bianchi, M. Kulmala, Continuous and comprehensive atmospheric observations in Beijing: A station to understand the complex urban atmospheric environment. *Big Earth Data* **4**, 295–321 (2020).

42. J. D. Haskins, L. Jaeglé, V. Shah, B. H. Lee, F. D. Lopez-Hilfiker, P. Campuzano-Jost, J. C. Schroder, D. A. Day, H. Guo, A. P. Sullivan, R. Weber, J. Dibb, T. Campos, J. L. Jimenez, S. S. Brown, J. A. Thornton, Wintertime gas-particle partitioning and speciation of inorganic chlorine in the lower troposphere over the Northeast United States and Coastal Ocean. *JGR Atmos.* **123**, 12897–12916 (2018).

43. B. H. Lee, F. D. Lopez-Hilfiker, P. R. Veres, E. E. McDuffie, D. L. Fibiger, T. L. Sparks, C. J. Ebben, J. R. Green, J. C. Schroder, P. Campuzano-Jost, S. Iyer, E. L. D'Ambro, S. Schobesberger, S. S. Brown, P. J. Wooldridge, R. C. Cohen, M. N. Fiddler, S. Bililign, J. L. Jimenez, T. Kurtén, A. J. Weinheimer, L. Jaegle, J. A. Thornton, Flight deployment of a high-resolution time-of-flight chemical ionization mass spectrometer: Observations of reactive halogen and nitrogen oxide species. *JGR Atmos.* **123**, 7670–7686 (2018).

44. G. M. Wolfe, M. R. Marvin, S. J. Roberts, K. R. Travis, J. Liao, The framework for 0-D atmospheric modeling (F0AM) v3.1. *Geosci. Model Dev.* **9**, 3309–3319 (2016).

45. M. E. Jenkin, J. C. Young, A. R. Rickard, The MCM v3.3.1 degradation scheme for isoprene. *Atmos. Chem. Phys.* **15**, 11433–11459 (2015).

46. M. Xia, T. Wang, Z. Wang, Y. Chen, X. Peng, Y. Huo, W. Wang, Q. Yuan, Y. Jiang, H. Guo, C. Lau, K. Leung, A. Yu, S. Lee, Pollution-derived Br_2 boosts oxidation power of the coastal atmosphere. *Environ. Sci. Technol.* **56**, 12055–12065 (2022).

47. M. Ammann, R. A. Cox, J. N. Crowley, M. E. Jenkin, A. Mellouki, M. J. Rossi, J. Troe, T. J. Wallington, Evaluated kinetic and photochemical data for atmospheric chemistry: Volume VI – Heterogeneous reactions with liquid substrates. *Atmos. Chem. Phys.* **13**, 8045–8228 (2013).

48. M. Deborde, U. von Gunten, Reactions of chlorine with inorganic and organic compounds during water treatment—Kinetics and mechanisms: A critical review. *Water Res.* **42**, 13–51 (2008).

49. M. Soni, R. Sander, L. K. Sahu, D. Taraborrelli, P. Liu, A. Patel, I. A. Girach, A. Pozzer, S. S. Gunthe, N. Ojha, Comprehensive multiphase chlorine chemistry in the box model CAABA/MECCA: Implications for atmospheric oxidative capacity. *Atmos. Chem. Phys.* **23**, 15165–15180 (2023).

50. T. Chen, C. Guestrin, "XGBoost: A scalable tree boosting system," *Proceedings of the 22nd ACM SIGKDD International Conference on Knowledge Discovery and Data Mining* (Association for Computing Machinery, 2016), pp. 785–794.

51. S. M. Lundberg, G. Erion, H. Chen, A. DeGrave, J. M. Prutkin, B. Nair, R. Katz, J. Himmelfarb, N. Bansal, S.-I. Lee, From local explanations to global understanding with explainable AI for trees. *Nat. Mach. Intell.* **2**, 56–67 (2020).

52. W. J. Requia, Q. Di, R. Silvern, J. T. Kelly, P. Koutrakis, L. J. Mickley, M. P. Sulprizio, H. Amini, L. Shi, J. Schwartz, An ensemble learning approach for estimating high spatiotemporal resolution of ground-level ozone in the contiguous United States. *Environ. Sci. Technol.* **54**, 11037–11047 (2020).

53. Y. Wang, Y. Zhao, Y. Liu, Y. Jiang, B. Zheng, J. Xing, Y. Liu, S. Wang, C. P. Nielsen, Sustained emission reductions have restrained the ozone pollution over China. *Nat. Geosci.* **16**, 967–974 (2023).

54. L. Hou, Q. Dai, C. Song, B. Liu, F. Guo, T. Dai, L. Li, B. Liu, X. Bi, Y. Zhang, Y. Feng, Revealing drivers of haze pollution by explainable machine learning. *Environ. Sci. Technol. Lett.* **9**, 112–119 (2022).

55. S. M. McNamara, A. R. W. Raso, S. Wang, S. Thanekar, E. J. Boone, K. R. Kolesar, P. K. Peterson, W. R. Simpson, J. D. Fuentes, P. B. Shepson, K. A. Pratt, Springtime nitrogen oxide-influenced chlorine chemistry in the Coastal Arctic. *Environ. Sci. Technol.* **53**, 8057–8067 (2019).

56. L. H. Mielke, A. Furgeson, H. D. Osthoff, Observation of $ClNO_2$ in a mid-continental urban environment. *Environ. Sci. Technol.* **45**, 8889–8896 (2011).

57. M. J. Lawler, R. Sander, L. J. Carpenter, J. D. Lee, R. von Glasow, R. Sommariva, E. S. Saltzman, HOCl and Cl_2 observations in marine air. *Atmos. Chem. Phys.* **11**, 7617–7628 (2011).

58. M. Priestley, M. le Breton, T. J. Bannan, S. D. Worrall, A. Bacak, A. R. D. Smedley, E. Reyes-Villegas, A. Mehra, J. Allan, A. R. Webb, D. E. Shallcross, H. Coe, C. J. Percival, Observations of organic and inorganic chlorinated compounds and their contribution to chlorine radical concentrations in an urban environment in northern Europe during the wintertime. *Atmos. Chem. Phys.* **18**, 13481–13493 (2018).

59. G. J. Phillips, M. J. Tang, J. Thieser, B. Brickwedde, G. Schuster, B. Bohn, J. Lelieveld, J. N. Crowley, Significant concentrations of nitryl chloride observed in rural continental Europe associated with the influence of sea salt chloride and anthropogenic emissions. *Geophys. Res. Lett.* **39**, L10811 (2012).

60. F. Li, D. D. Huang, W. Nie, Y. J. Tham, S. Lou, Y. Li, L. Tian, Y. Liu, M. Zhou, H. Wang, L. Qiao, H. Wang, Z. Wang, C. Huang, Y. J. Li, Observation of nitrogen oxide-influenced chlorine chemistry and source analysis of Cl_2 in the Yangtze River Delta, China. *Atmos. Environ.* **306**, 119829 (2023).

61. D. Jeong, R. Seco, D. Gu, Y. Lee, B. A. Nault, C. J. Knote, T. McGee, J. T. Sullivan, J. L. Jimenez, P. Campuzano-Jost, D. R. Blake, D. Sanchez, A. B. Guenther, D. Tanner, L. G. Huey, R. Long, B. E. Anderson, S. R. Hall, K. Ullmann, H.-J. Shin, S. C. Herndon, Y. Lee, D. Kim, J. Ahn, S. Kim, Integration of airborne and ground observations of nitryl chloride in the Seoul metropolitan area and the implications on regional oxidation capacity during KORUS-AQ 2016. *Atmos. Chem. Phys.* **19**, 12779–12795 (2019).

62. R. Dörich, P. Eger, J. Lelieveld, J. N. Crowley, Iodide CIMS and m/z 62: The detection of HNO_3 as NO_3^- in the presence of PAN, peroxyacetic acid and ozone. *Atmos. Meas. Tech.* **14**, 5319–5332 (2021).

63. T. H. Bertram, J. A. Thornton, T. P. Riedel, An experimental technique for the direct measurement of N_2O_5 reactivity on ambient particles. *Atmos. Meas. Tech.* **2**, 231–242 (2009).

64. Y. J. Tham, C. Yan, L. Xue, Q. Zha, X. Wang, T. Wang, Presence of high nitryl chloride in Asian coastal environment and its impact on atmospheric photochemistry. *Chin. Sci. Bull.* **59**, 356–359 (2013).

65. B. J. Finlayson-Pitts, The tropospheric chemistry of sea salt: A molecular-level view of the chemistry of NaCl and NaBr. *Chem. Rev.* **103**, 4801–4822 (2003).

66. J. P. Kercher, T. P. Riedel, J. A. Thornton, Chlorine activation by N_2O_5: Simultaneous, in situ detection of $ClNO_2$ and N_2O_5 by chemical ionization mass spectrometry. *Atmos. Meas. Tech.* **2**, 193–204 (2009).

67. D. Bolton, The computation of equivalent potential temperature. *Mon. Weather Rev.* **108**, 1046–1053 (1980).

68. X. Peng, T. Wang, W. Wang, A. R. Ravishankara, C. George, M. Xia, M. Cai, Q. Li, C. M. Salvador, C. Lau, X. Lyu, C. N. Poon, A. Mellouki, Y. Mu, M. Hallquist, A. Saiz-Lopez, H. Guo, H. Herrmann, C. Yu, J. Dai, Y. Wang, X. Wang, A. Yu, K. Leung, S. Lee, J. Chen, Photodissociation of particulate nitrate as a source of daytime tropospheric Cl_2. *Nat. Commun.* **13**, 939 (2022).

69. Z. An, R. Yin, X. Zhao, X. Li, Y. Li, Y. Yuan, J. Guo, Y. Zhao, X. Li, D. Li, Y. Li, D. Wang, C. Yan, K. He, D. R. Worsnop, F. N. Keutsch, J. Jiang, Molecular and seasonal characteristics of organic vapors in urban Beijing: Insights from Vocus-PTR measurements. *Atmos. Chem. Phys.* **24**, 13793–13810 (2024).

70. B. Yuan, A. R. Koss, C. Warneke, M. Coggon, K. Sekimoto, J. A. de Gouw, Proton-transfer-reaction mass spectrometry: Applications in atmospheric sciences. *Chem. Rev.* **117**, 13187–13229 (2017).

71. K. Sekimoto, S.-M. Li, B. Yuan, A. Koss, M. Coggon, C. Warneke, J. de Gouw, Calculation of the sensitivity of proton-transfer-reaction mass spectrometry (PTR-MS) for organic trace gases using molecular properties. *Int. J. Mass Spectrom.* **421**, 71–94 (2017).

72. L. Cui, D. Wu, S. Wang, Q. Xu, R. Hu, J. Hao, Measurement report: Ambient volatile organic compound (VOC) pollution in urban Beijing: Characteristics, sources, and implications for pollution control. *Atmos. Chem. Phys.* **22**, 11931–11944 (2022).

73. S. L. Haslett, D. M. Bell, V. Kumar, J. G. Slowik, D. S. Wang, S. Mishra, N. Rastogi, A. Singh, D. Ganguly, J. Thornton, F. Zheng, Y. Li, W. Nie, Y. Liu, W. Ma, C. Yan, M. Kulmala, K. R. Daellenbach, D. Hadden, U. Baltensperger, A. S. H. Prevot, S. N. Tripathi, C. Mohr, Nighttime NO emissions strongly suppress chlorine and nitrate radical formation during the winter in Delhi. *Atmos. Chem. Phys.* **23**, 9023–9036 (2023).

74. I. Trebs, B. Bohn, C. Ammann, U. Rummel, M. Blumthaler, R. Königstedt, F. X. Meixner, S. Fan, M. O. Andreae, Relationship between the NO_2 photolysis frequency and the solar global irradiance. *Atmos. Meas. Tech.* **2**, 725–739 (2009).

75. T. C. Clark, M. A. A. Clyne, Kinetic mechanisms in nitrogen-chlorine radical systems. Part 1.—The formation and detection of the NCl_2 and N_3 free radicals using time-resolved absorption spectrophotometry. *Trans. Faraday Soc.* **65**, 2994–3004 (1969).

76. R. Yin, E. R. Blatchley III, C. Shang, UV photolysis of mono- and dichloramine using UV-LEDs as radiation sources: Photodecay rates and radical concentrations. *Environ. Sci. Technol.* **54**, 8420–8429 (2020).

77. A. Marini, A. Muñoz-Losa, A. Biancardi, B. Mennucci, What is solvatochromism? *J. Phys. Chem. B.* **114**, 17128–17135 (2010).

78. L. T. Molina, S. D. Schinke, M. J. Molina, Ultraviolet absorption spectrum of hydrogen peroxide vapor. *Geophys. Res. Lett.* **4**, 580–582 (1977).

79. J. M. González-Sánchez, N. Brun, J. Wu, S. Ravier, J. L. Clément, A. Monod, On the importance of multiphase photolysis of organic nitrates on their global atmospheric removal. *Atmos. Chem. Phys.* **23**, 5851–5866 (2023).

80. J. Wisniak, Pierre Louis Dulong. *Educ. Quím.* **12**, 219–228 (2001).

81. B. S. Yiin, D. M. Walker, D. W. Margerum, Nonmetal redox kinetics: General-acid-assisted reactions of chloramine with sulfite and hydrogen sulfite. *Inorg. Chem.* **26**, 3435–3441 (1987).

82. E. R. Lewis, An examination of Köhler theory resulting in an accurate expression for the equilibrium radius ratio of a hygroscopic aerosol particle valid up to and including relative humidity 100%. *JGR Atmos.* **113**, 2007JD008590 (2008).

83. M. Xia, X. Chen, W. Ma, Y. Guo, R. Yin, J. Zhan, Y. Zhang, Z. Wang, F. Zheng, J. Xie, Y. Wang, C. Hua, Y. Liu, C. Yan, M. Kulmala, Observations and modeling of gaseous nitrated phenols in Urban Beijing: Insights from seasonal comparison and budget analysis. *JGR Atmos.* **128**, e2023JD039551 (2023).

84. J. L. Serras, S. Vinga, A. M. Carvalho, Outlier detection for multivariate time series using dynamic bayesian networks. *Appl. Sci.* **11**, 1955 (2021).

85. J. Burkholder, S. Sander, J. Abbatt, J. Barker, C. Cappa, J. Crounse, T. Dibble, R. Huie, C. Kolb, M. Kurylo, "Chemical kinetics and photochemical data for use in atmospheric studies" (JPL Publication No. 19-5, Jet Propulsion Laboratory, 2020).

86. Z. Finewax, D. Pagonis, M. S. Claflin, A. V. Handschy, W. L. Brown, O. Jenks, B. A. Nault, D. A. Day, B. M. Lerner, J. L. Jimenez, P. J. Ziemann, J. A. de Gouw, Quantification and source characterization of volatile organic compounds from exercising and application of chlorine-based cleaning products in a university athletic center. *Indoor Air* **31**, 1323–1339 (2021).

87. A. Moravek, T. C. VandenBoer, Z. Finewax, D. Pagonis, B. A. Nault, W. L. Brown, D. A. Day, A. V. Handschy, H. Stark, P. Ziemann, J. L. Jimenez, J. A. de Gouw, C. J. Young, Reactive chlorine emissions from cleaning and reactive nitrogen chemistry in an indoor athletic facility. *Environ. Sci. Technol.* **56**, 15408–15416 (2022).

88. G. Predieri, P. Giacobazzi, Determination of nitrogen trichloride (NCl3) levels in the air of indoor chlorinated swimming pools: An impinger method proposal. *Int. J. Environ. Anal. Chem.* **92**, 645–654 (2012).

89. L. R. Crilley, J. C. Ditto, M. Lao, Z. Zhou, J. P. D. Abbatt, A. W. H. Chan, T. C. VandenBoer, Commercial kitchen operations produce a diverse range of gas-phase reactive nitrogen species. *Environ. Sci. Processes Impacts* **27**, d4em00491d (2025).

90. N. Bhattacharyya, M. Tang, D. C. Blomdahl, L. G. Jahn, P. Abue, D. T. Allen, R. L. Corsi, A. Novoselac, P. K. Misztal, L. Hildebrandt Ruiz, Bleach emissions interact substantially with surgical and KN95 mask surfaces. *Environ. Sci. Technol.* **57**, 6589–6598 (2023).

91. A. D. Stubbs, M. Lao, C. Wang, J. P. D. Abbatt, J. Hoffnagle, T. C. VandenBoer, T. F. Kahan, Near-source hypochlorous acid emissions from indoor bleach cleaning. *Environ Sci Process Impacts* **25**, 56–65 (2023).

92. A. R. Jensen, M. A. Morris, B. C. Schulze, A. C. Bradley, L. D. Anderson, O. J. Jenks, W. D. Dresser, K. Ball, R. X. Ward, D. A. Day, J. D. Crounse, S. Meinardi, B. Barletta, D. R. Blake, J. H. Seinfeld, P. O. Wennberg, J. L. Jimenez, J. A. de Gouw, Emissions and chemistry of volatile organic compounds in the los angeles basin in summer 2022. *JGR Atmos.* **129**, e2024JD041812 (2024).

93. J. H. Lee, I. N. Tang, Accommodation coefficient of gaseous NO_2 on water surfaces. *Atmos. Environ.* **22**, 1147–1151 (1988).

94. J. Boniface, Q. Shi, Y. Q. Li, J. L. Cheung, O. V. Rattigan, P. Davidovits, D. R. Worsnop, J. T. Jayne, C. E. Kolb, Uptake of gas-phase SO_2, H_2S, and CO_2 by aqueous solutions. *Chem. A Eur. J.* **104**, 7502–7510 (2000).

95. N. M. Rubtsov, Kinetic mechanism and chemical oscillations in the branching chain decomposition of nitrogen trichloride. *Mendeleev Commun.* **8**, 173–175 (1998).

96. N. M. Rubtsov, V. D. Kotelkin, V. P. Karpov, Transition of flame propagation from isothermal to thermal regimes in chain processes with nonlinear chain branching. *Kinet. Catal.* **45**, 1–9 (2004).

97. Z. Qiang, C. D. Adams, Determination of monochloramine formation rate constants with stopped-flow spectrophotometry. *Environ. Sci. Technol.* **38**, 1435–1444 (2004).

98. R. E. Corbett, W. S. Metcalf, F. G. Soper, 395. Studies of N-halogeno-compounds. Part IV. The reaction between ammonia and chlorine in aqueous solution, and the hydrolysis constants of chloroamines. *J. Chem. Soc.*, 1927–1929 (1953).

99. D. W. Margerum, L. M. Schurter, J. Hobson, E. E. Moore, Water chlorination chemistry: Nonmetal redox kinetics of chloramine and nitrite ion. *Environ. Sci. Tech.* **28**, 331–337 (1994).

100. Y.-H. Chuang, S. Chen, C. J. Chinn, W. A. Mitch, Comparing the UV/monochloramine and UV/free chlorine advanced oxidation processes (AOPs) to the UV/hydrogen peroxide AOP under scenarios relevant to potable reuse. *Environ. Sci. Technol.* **51**, 13859–13868 (2017).

101. S. Patton, W. Li, K. D. Couch, S. P. Mezyk, K. P. Ishida, H. Liu, Impact of the ultraviolet photolysis of monochloramine on 1,4-dioxane removal: New insights into potable water reuse. *Environ. Sci. Technol. Lett.* **4**, 26–30 (2017).

102. S. Patton, M. Romano, V. Naddeo, K. P. Ishida, H. Liu, Photolysis of mono- and dichloramines in UV/hydrogen peroxide: Effects on 1,4-dioxane removal and relevance in water reuse. *Environ. Sci. Technol.* **52**, 11720–11727 (2018).

103. J. C. Morris, R. A. Isaac, “A critical review of kinetic and thermodynamic constants for the aqueous chlorine-ammonia system,” in *Water Chlorination: Environmental Impact and Health Effects* (Ann Arbor Science Publisher Inc., 1983), vol. 4, pp. 49–62.

104. S. Hossain, C. W. K. Chow, D. Cook, E. Sawade, G. A. Hewa, Review of chloramine decay models in drinking water system. *Environ. Sci. Water Res. Technol.* **8**, 926–948 (2022).

105. P. P. Fehér, M. Purgel, A. Lengyel, A. Stirling, I. Fábián, The mechanism of monochloramine disproportionation under acidic conditions. *Dalton Trans.* **48**, 16713–16721 (2019).

106. Z. Zhang, Y. H. Chuang, N. Huang, W. A. Mitch, Predicting the contribution of chloramines to contaminant decay during ultraviolet/hydrogen peroxide advanced oxidation process treatment for potable reuse. *Environ. Sci. Technol.* **53**, 4416–4425 (2019).

107. V. C. Hand, D. W. Margerum, Kinetics and mechanisms of the decomposition of dichloramine in aqueous solution. *Inorg. Chem.* **22**, 1449–1456 (1983).

108. K. Kumar, R. W. Shinness, D. W. Margerum, Kinetics and mechanisms of the base decomposition of nitrogen trichloride in aqueous solution. *Inorg. Chem.* **26**, 3430–3434 (1987).

109. S. M. McNamara, N. M. Garner, S. Wang, A. R. W. Raso, S. Thanekar, A. J. Barget, J. D. Fuentes, P. B. Shepson, K. A. Pratt, Bromine chloride in the Coastal Arctic: Diel patterns and production mechanisms. *ACS Earth Space Chem.* **4**, 620–630 (2020).

110. J. B. Pernov, R. Bossi, T. Lebourgeois, J. K. Nøjgaard, R. Holzinger, J. L. Hjorth, H. Skov, Atmospheric VOC measurements at a High Arctic site: Characteristics and source apportionment. *Atmos. Chem. Phys.* **21**, 2895–2916 (2021).

111. Y. Jiang, E. H. Hoffmann, A. Tilgner, M. B. E. Aiyuk, S. T. Andersen, L. Wen, M. van Pinxteren, H. Shen, L. Xue, W. Wang, H. Herrmann, Insights into NO and HONO chemistry in the tropical marine boundary layer at Cape Verde during the MarParCloud campaign. *JGR Atmos.* **128**, e2023JD038865 (2023).

112. Q. Chen, M. Xia, X. Peng, C. Yu, P. Sun, Y. Li, Y. Liu, Z. Xu, Z. Xu, R. Wu, W. Nie, A. Ding, Y. Zhao, T. Wang, Large daytime molecular chlorine missing source at a suburban site in East China. *JGR Atmos.* **127**, e2021JD035796 (2022).

113. Y. Qu, Y. Han, Y. Wu, P. Gao, T. Wang, Study of PBLH and its correlation with particulate matter from one-year observation over Nanjing, Southeast China. *Remote Sens.* **9**, 668 (2017).

114. G. R. Wentworth, J. G. Murphy, B. Croft, R. V. Martin, J. R. Pierce, J. S. Côté, I. Courchesne, J. É. Tremblay, J. Gagnon, J. L. Thomas, S. Sharma, D. Toom-Sauntry, A. Chivulescu, M. Levasseur, J. P. D. Abbatt, Ammonia in the summertime Arctic marine boundary layer: Sources, sinks, and implications. *Atmos. Chem. Phys.* **16**, 1937–1953 (2016).

115. R. Sander, A. A. P. Pszenny, W. C. Keene, E. Crete, B. Deegan, M. S. Long, J. R. Maben, A. H. Young, Gas phase acid, ammonia and aerosol ionic and trace element concentrations at Cape Verde during the Reactive Halogens in the Marine Boundary Layer (RHaMBLe) 2007 intensive sampling period. *Earth Syst. Sci. Data* **5**, 385–392 (2013).

116. W. L. Chang, P. V. Bhave, S. S. Brown, N. Riemer, J. Stutz, D. Dabdub, Heterogeneous atmospheric chemistry, ambient measurements, and model calculations of N_2O_5: A review. *Aerosol Sci. Tech.* **45**, 665–695 (2011).

117. X. Zhang, S. Tong, C. Jia, W. Zhang, Z. Wang, G. Tang, B. Hu, Z. Liu, L. Wang, P. Zhao, Y. Pan, M. Ge, Elucidating HONO formation mechanism and its essential contribution to OH during haze events. *npj Clim. Atmos. Sci.* **6**, 55 (2023).
